# Deubiquitination of Vangl by USP6 and USP32 Regulates Planar Cell Polarity Signaling

**DOI:** 10.1002/advs.77496

**Published:** 2026-08-30

**Authors:** Fangzi Zha, Di Feng, Ziru Xue, Zhiying Liu, Xiaochen Lin, Zhe Wang, Ling Xu, Jasper Fuk Woo Chan, Martin Cheung, Michael Shing Yan Huen, Yusong Guo, Kui Ming Chan, Yun Liu, Bo Gao

**Affiliations:** ^1^ School of Biomedical Sciences Faculty of Medicine The Chinese University of Hong Kong Hong Kong SAR China; ^2^ Shenzhen Research Institute The Chinese University of Hong Kong Shenzhen China; ^3^ School of Biomedical Sciences Li Ka Shing Faculty of Medicine The University of Hong Kong Hong Kong SAR China; ^4^ Shanghai Key Laboratory of Cancer Systems Regulation and Clinical Translation Shanghai Cancer Institute Renji Hospital School of Medicine Shanghai Jiao Tong University Shanghai China; ^5^ School of Health Science and Engineering University of Shanghai for Science and Technology Shanghai China; ^6^ Department of Microbiology School of Clinical Medicine Li Ka Shing Faculty of Medicine The University of Hong Kong Hong Kong SAR China; ^7^ Division of Life Science Hong Kong University of Science and Technology Hong Kong SAR China; ^8^ Department of Biomedical Sciences College of Biomedicine City University of Hong Kong Hong Kong SAR China; ^9^ Key Laboratory of Regenerative Medicine Ministry of Education School of Biomedical Sciences Faculty of Medicine The Chinese University of Hong Kong Hong Kong SAR China; ^10^ Kunming Institute of Zoology – The Chinese University of Hong Kong (KIZ‐CUHK) Joint Laboratory of Bioresources and Molecular Research of Common Diseases The Chinese University of Hong Kong Hong Kong SAR China; ^11^ Centre for Translational Stem Cell Biology Hong Kong SAR China

**Keywords:** cell biology, deubiquitinating enzyme, deubiquitination, pancreatic cancer, planar cell polarity, ubiquitin

## Abstract

Planar cell polarity (PCP) signaling is an evolutionarily conserved mechanism regulating polarized cellular and tissue behaviors in diverse morphogenetic and physiological processes. Disruption or aberrant activation of PCP signaling can cause developmental defects or promote cancer malignancy. Vangl1 and Vangl2 are redundant core PCP components, and their protein levels are tightly controlled to maintain appropriate PCP signaling. Here, we identify two deubiquitinases, USP6 and USP32, that stabilize Vangl proteins through distinct ubiquitin linkage‐specific mechanisms. USP6 primarily removes K33‐linked and multi‐monoubiquitin signals from plasma membrane‐associated Vangl2, whereas USP32 removes K48‐linked ubiquitin chains from Golgi/ER‐localized Vangl2, thereby regulating distinct subcellular pools of Vangl2. Given that *USP6* was previously reported to be a hominoid‐specific gene originating from *USP32*, our findings suggest evolutionary refinement in PCP regulation. Consistent with these biochemical functions, genetic interaction studies demonstrate that *Usp32* cooperates with *Vangl* genes to regulate PCP signaling during mouse embryogenesis. In pancreatic ductal adenocarcinoma, USP32 and VANGL1 are markedly upregulated and functionally promote cancer cell migration and metastasis. USP32 enhances metastatic behavior by stabilizing VANGL1 proteins. Together, our findings uncover a previously unrecognized regulatory mechanism of PCP signaling through USP6/USP32‐mediated deubiquitination of Vangl proteins and highlight its roles in both developmental morphogenesis and cancer progression.

## Graphical abstract

Compartment‐specific deubiquitination controls Vangl dosage and planar cell polarity signaling. USP6 and USP32 regulate distinct subcellular pools of Vangl by removing distinct ubiquitin modifications from Vangl proteins. This regulatory mechanism safeguards PCP‐dependent embryonic morphogenesis, while aberrant USP32‐dependent stabilization of VANGL promotes pancreatic ductal adenocarcinoma cell migration and metastasis.

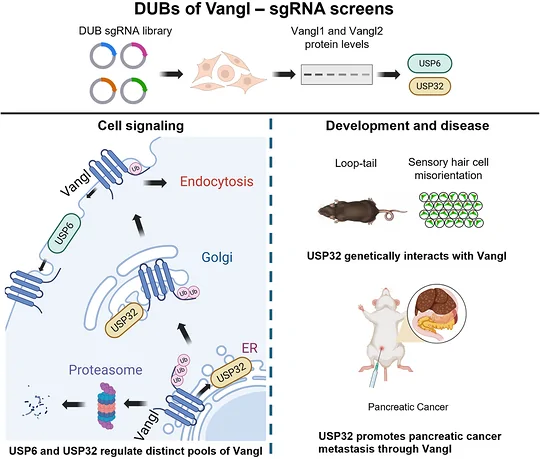

## 1 Introduction

Planar cell polarity (PCP) represents a fundamental organizing principle in developmental biology, governing the coordinated alignment of cellular structures and behaviors across the tissue plane [1, 2, 3, 4, 5]. This evolutionarily conserved process, first elucidated in *Drosophila* models, is indispensable for a variety of morphogenetic events in vertebrates, including convergent extension, neural tube closure, left‐right symmetry breaking, and the orientation of sensory hair cells [6, 7, 8, 9, 10, 11]. PCP signaling is mediated by a set of core proteins, classically including Frizzled (Fz), Van Gogh (Vang), Flamingo (Fmi), Dishevelled (Dsh), Prickle (Pk), and Diego (Dgo), which become asymmetrically distributed at the cell cortex to translate directional information into polarized cell behaviors [1, 2, 3, 4, 5]. Not surprisingly, perturbation of PCP signaling causes diverse developmental defects and is increasingly linked to tumor progression [5, 12, 13]. Among these core PCP proteins, Vangl1 and Vangl2 are particularly important because they are dedicated four‐pass transmembrane PCP proteins and their activity in mammals is highly dosage‐sensitive in a functionally redundant manner. Genetic studies in mice established *Vangl2* as the gene mutated in the classic *loop‐tail* model and further showed strong genetic interaction between *Vangl1* and *Vangl2* in neural tube closure and other PCP‐dependent processes [9, 10, 14, 15]. Human genetic studies further reinforced the clinical importance of *VANGL* genes. *VANGL1* and *VANGL2* rare variants have been identified in patients with neural tube defects and linked to congenital vertebral malformations [16, 17, 18].

PCP signaling relies on precise stoichiometry and stability of core PCP proteins, because polarized signaling emerges from their dynamic assembly, trafficking, and turnover. Multiple core PCP components are tightly regulated by post‐translational modifications (PTMs), which influence their localization, trafficking, stability, and signaling activity [19, 20, 21, 22, 23, 24, 25, 26, 27, 28, 29, 30, 31, 32, 33, 34, 35]. These studies support a general principle that PCP output is controlled not only by gene expression, but also by PTM‐dependent tuning of core protein abundance and subcellular distribution. Our previous work showed that Wnt5a induces phosphorylation of Vangl proteins through CK1ε/δ, and Vangl phosphorylation is dose‐dependently required for mammalian PCP [20, 27, 36]. Ubiquitination provides a second major layer of Vangl regulation. In our previous study, we found that the C‐terminus of Vangl2 directly binds the ATPase p97/VCP, a key component in the endoplasmic reticulum‐associated degradation (ERAD) pathway, through a conserved VCP‐interacting motif. This interaction recruits the E3 ligase CUL3‐KBTBD7 to promote Vangl ubiquitination and ERAD. Importantly, Wnt5a/CK1‐dependent phosphorylation opposes this process by facilitating Vangl export from the ER to the plasma membrane, revealing direct crosstalk between phosphorylation and ubiquitination in controlling Vangl [31, 37]. Vangl ubiquitination is also regulated by RNF43, a cell surface E3 ligase, which suppresses Wnt5a‐driven non‐canonical signaling and promotes Vangl2 ubiquitination [38], suggesting that distinct E3 ligases may target different subcellular pools of Vangl.

Because ubiquitination is reversible, a full understanding of Vangl regulation requires identifying the deubiquitinating enzymes (DUBs) that counterbalance these E3 ligases. DUBs comprise a large enzyme superfamily that removes or edits ubiquitin chains, thereby rescuing substrates from degradation, changing ubiquitin linkage architecture, or redirecting cargoes between trafficking pathways. More than 90 DUBs are encoded in the human genome and can be classified into several families, among which ubiquitin‐specific proteases (USPs) form the largest group [39, 40]. Functionally, DUBs are not merely passive antagonists of E3 ligases. They are also active determinants of protein fate, controlling protein stability, membrane trafficking, endosomal sorting, receptor recycling, and signaling duration. Consistent with these broad functions, DUB dysregulation has been implicated in developmental abnormalities, inflammatory disorders, and cancer, and DUBs are increasingly recognized as therapeutically actionable regulators of signaling pathways [41, 42, 43, 44]. Thus, identifying the DUBs that oppose Vangl ubiquitination is important for understanding how PCP signaling is precisely maintained within a physiological range.

**FIGURE 1:**
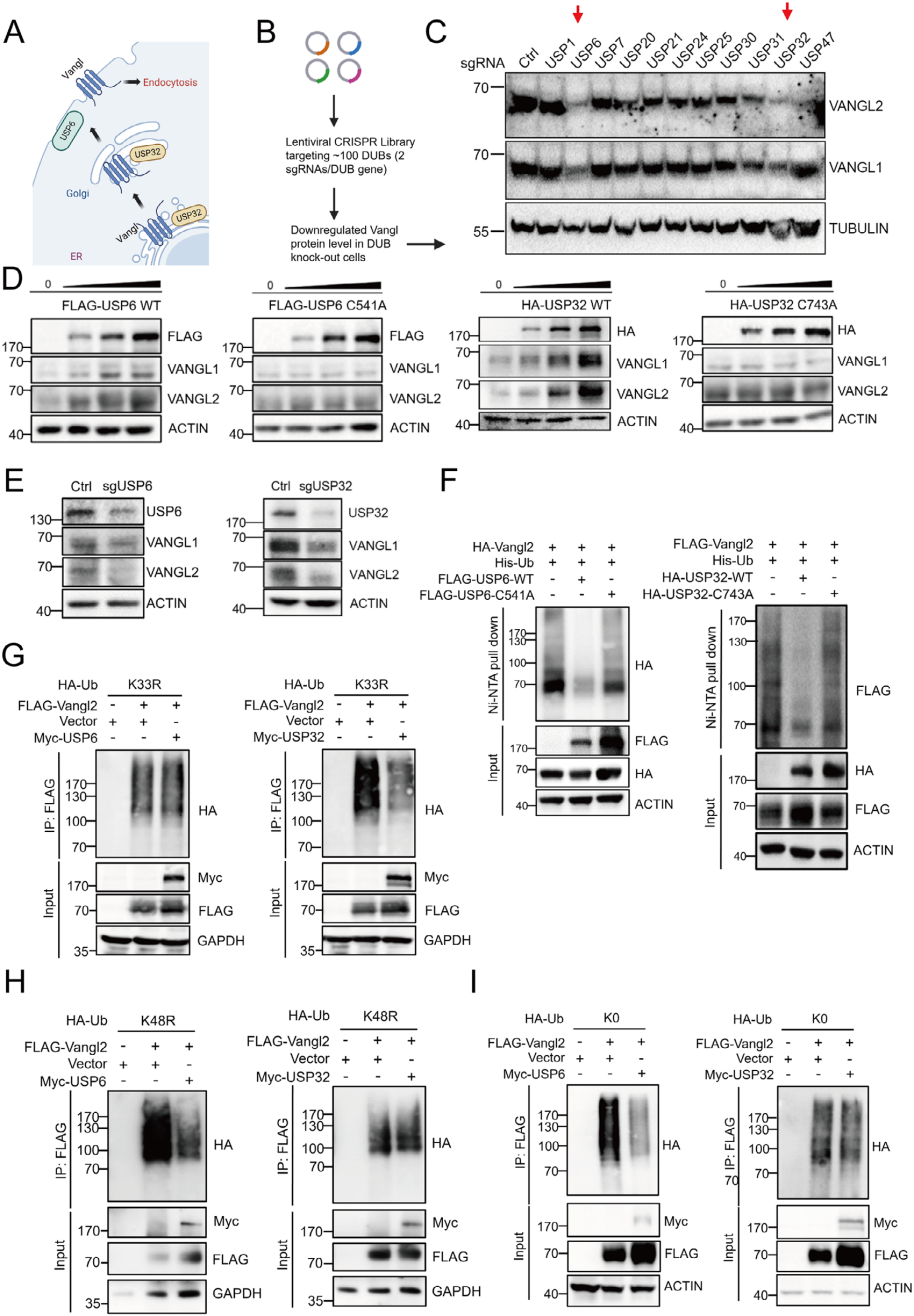
USP6 and USP32 stabilize Vangl proteins through deubiquitination. (A) Schematic localization of USP6, USP32, and Vangl proteins in cells. (B) Schematic of the CRISPR/Cas9‐based DUB screen used to identify deubiquitinases regulating Vangl protein stability. A lentiviral sgRNA library targeting DUB genes was introduced into HEK293T cells, followed by analysis of endogenous Vangl1 and Vangl2 protein levels. (C) Depletion of USP6 or USP32 decreased endogenous Vangl1 and Vangl2 protein levels. (D) Overexpression of wild‐type FLAG‐tagged USP6 or HA‐tagged USP32, but not the catalytic mutants USP6‐C541A or USP32‐C743A, increased Vangl1 and Vangl2 protein levels. (E) Knockdown of USP6 and USP32 decreased endogenous Vangl1 and Vangl2 protein levels. (F) Wild‐type USP6 or USP32, but not their catalytic mutants, reduced ubiquitination of Vangl2 in HEK293T cells. (G–I) Ubiquitin linkage analysis of Vangl2 deubiquitination by USP6 and USP32. The original blot can be found in Figure S16.

Here, we identify USP6 and USP32 as two deubiquitinases that stabilize Vangl proteins through distinct subcellular mechanisms. We show that USP6 primarily acts on plasma membrane‐accessible Vangl, whereas USP32 regulates a Golgi/ER‐associated Vangl pool, thereby revealing compartment‐specific control of Vangl stability (Figure 1A). Interestingly, *USP6* is a hominoid‐specific gene with the chimeric fusion of *TBC1D3* and *USP32* [45]. Functionally, we demonstrate that *Usp32* genetically interacts with *Vangl1 and Vangl2* in mouse PCP signaling, that *USP32‐VANGL1* signaling promotes human pancreatic cancer cell migration and metastasis, and that loss of *Usp32* disrupts pancreatic duct morphogenesis during mouse development. Together, our findings uncover a previously unrecognized deubiquitination layer in the control of Vangl dosage and provide a mechanistic framework for how PCP signaling is tuned into both normal morphogenesis and pancreatic cancer.

## 2 Results

### 2.1 USP6 and USP32 Stabilize Vangl Proteins Through Deubiquitination

To identify deubiquitinases (DUBs) that counteract Vangl ubiquitination, we performed a CRISPR/Cas9‐mediated DUB knockout screen in HEK293T cells. A lentiviral library comprising 188 sgRNAs targeting 94 DUBs (2 sgRNAs for each DUB) was used to generate pooled stable cell lines, and endogenous Vangl1 and Vangl2 protein levels were monitored (Figure 1B,C). We found that depletion of either USP6 or USP32 significantly reduced endogenous Vangl1 and Vangl2 compared to the mock control (Figure 1C). Conversely, overexpression of wild‐type USP6 or USP32 increased endogenous Vangl1 and Vangl2 protein levels in a dose‐dependent manner, whereas catalytically inactive mutants (USP6‐C541A and USP32‐C743A) failed to do so (Figure 1D). Consistently, knockdown of USP6 or USP32 decreased Vangl1 and Vangl2 protein levels (Figure 1E) and accelerated Vangl degradation in cycloheximide chase assays (Figure S1A). Because Wnt5a has been shown to regulate Vangl stability [31], we asked whether it also affects USP6 or USP32 abundance. Overexpression of Wnt5a did not alter USP6 or USP32 protein levels (Figure S1B), suggesting that Wnt5a does not regulate these DUBs to control Vangl levels.

We next tested whether USP6 and USP32 directly regulate Vangl ubiquitination. Indeed, wild‐type USP6 or USP32, but not their catalytically inactive mutants (USP6‐C541A and USP32‐C743A), markedly reduced polyubiquitination of Vangl2 (Figure 1F). To determine which ubiquitin chain linkage of Vangl is targeted by USP6 and USP32, we used ubiquitin lysine (K)‐to‐arginine (R) mutants (Ub‐KR), in which only one of the seven active lysine sites of ubiquitin is mutated to arginine (K6R, K11R, K27R, K29R, K33R, K48R, K63R). We found that Ub‐K33R, but not other Ub‐KR mutants, abrogated USP6‐mediated deubiquitination of Vangl2 (Figure 1G; Figure S2), indicating that USP6 preferentially removed K33‐linked chains from Vangl2. In contrast, Ub‐K48R selectively blocked USP32‐mediated deubiquitination of Vangl2 (Figure 1H; Figure S2), supporting the role of USP32 in removing K48‐linked ubiquitin chains. To examine whether USP6 and USP32 also regulate mono‐ or multi‐mono‐ubiquitination of Vangl2, we performed ubiquitination assays using lysine‐less ubiquitin (Ub‐K0), which allows mono‐ or multi‐mono‐ubiquitination but prevents polyubiquitin chain extension. USP6, but not USP32, reduced Ub‐K0‐modified Vangl2 (Figure 1I), indicating that USP6 can also remove multi‐monoubiquitin signals from Vangl2. Together, these data identify USP6 and USP32 as DUBs that stabilize Vangl proteins by removing distinct ubiquitin linkages.

### 2.2 USP6 and USP32 Interact with Vangl2 and Localize to Distinct Subcellular Compartments

**FIGURE 2:**
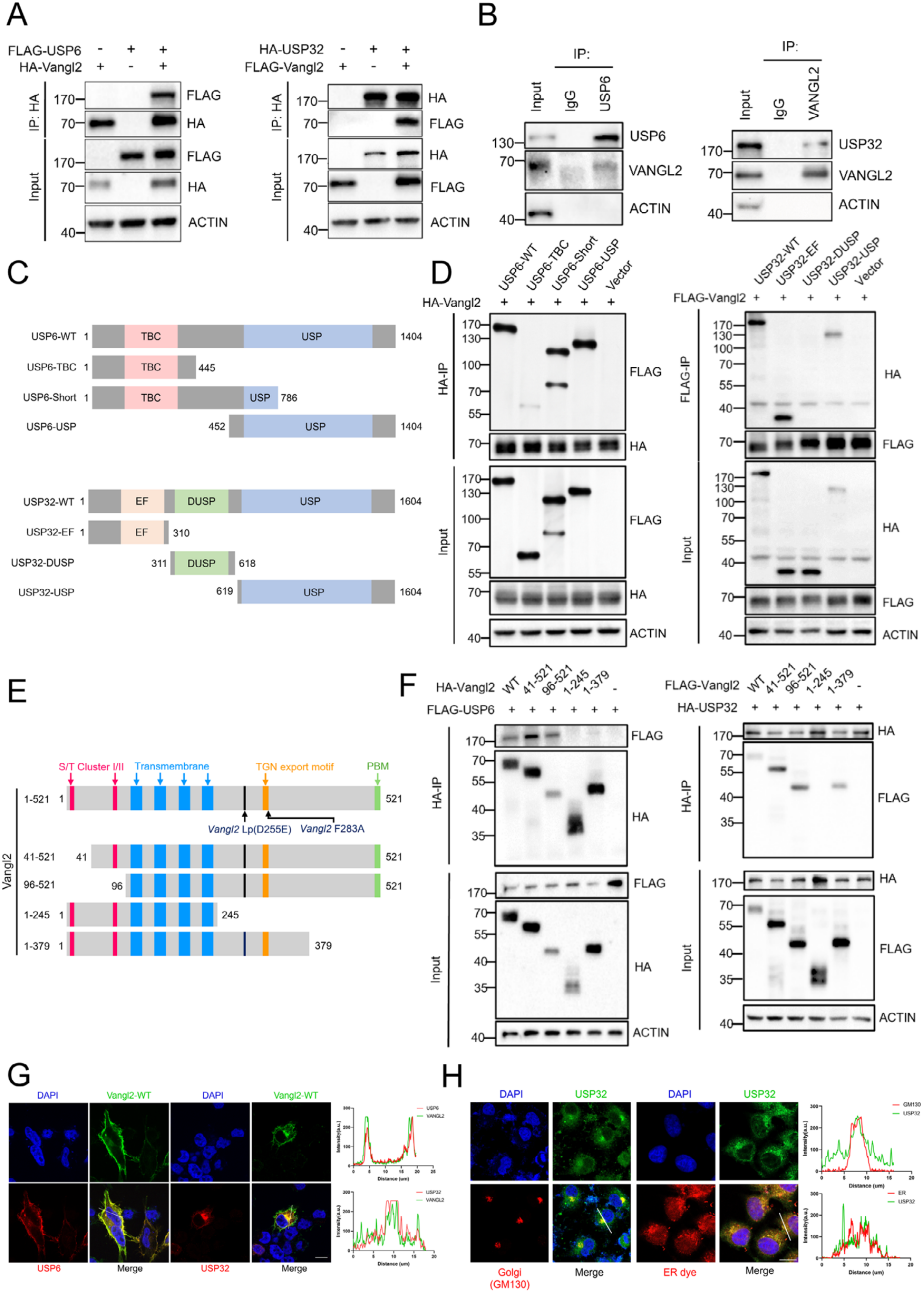
USP6 and USP32 interact with Vangl and localize to distinct subcellular compartments. (A) Co‐immunoprecipitation of overexpressed Vangl2 with USP6 or USP32 in HEK293T cells. (B) Endogenous Vangl2 co‐immunoprecipitated with USP6 or USP32 in HEK293T cells. (C) Schematic diagram of USP6 and USP32 protein domains. (D) Mapping of the Vangl2‐binding regions with USP6 and USP32. (E) Schematic diagram of Vangl2 domains. (F) The C‐terminal region of Vangl2 is required for binding to USP6 and USP32. (G) Subcellular localization of overexpressed USP6 and USP32 with Vangl2 in HeLa cells. USP6 co‐localized with Vangl2 at the plasma membrane, whereas USP32 was enriched at the Golgi (GM130). (H) Subcellular localization of endogenous USP32 in HeLa cells. USP32 was localized to both the Golgi (GM130) and ER (ER dye). G, H) Co‐localization analyses are shown in the right panels. Scale bar = 10 um. The original blot can be found in Figure S16.

Because their catalytic activities on Vangl imply physical proximity, we examined whether USP6 and USP32 are associated with Vangl. Co‐immunoprecipitation assays confirmed that FLAG‐tagged USP6 was enriched in HA‐Vangl2 precipitates and that Vangl2 co‐precipitated with HA‐USP32 (Figure 2A). Interactions between endogenous USP6 or USP32 and Vangl2 were also detected (Figure 2B). To define the structural basis of these interactions, we performed domain‐mapping analyses. We found Vangl2 mainly interacts with the N‐terminal portion of the USP domain of USP6 and binds the EF‐hand region and the USP domain of USP32 (Figure 2C,D). Consistent with the C‐terminus of Vangl serving as a major interaction hub, the Vangl2 C‐terminus was required for binding to both USP6 and USP32 (Figure 2E,F). We next examined the subcellular distribution of these complexes. Immunofluorescence analysis revealed that USP6 co‐localizes with Vangl2 at the plasma membrane, whereas USP32 is enriched at the Golgi (GM130), where it overlaps with a pool of Vangl2 (Figure 2G). Further analysis showed that USP32 is distributed in both the Golgi and the ER (Figure 2H).

### 2.3 USP6 and USP32 Regulate Vangl2 Through Compartment‐Specific Mechanisms

The distinct localization of USP6 and USP32 suggested that they might act on different Vangl pools. Newly synthesized Vangl proteins enter the secretory pathway in the ER and are transported to the plasma membrane via the Golgi, where they are subsequently maintained at the cell surface and can undergo endocytosis and recycling (Figure 1A) [31]. To leverage these trafficking steps to dissect compartment‐specific regulation, we analyzed Vangl2‐wild type (WT) and a set of Vangl2 variants that preferentially localize to distinct subcellular compartments.

**FIGURE 3:**
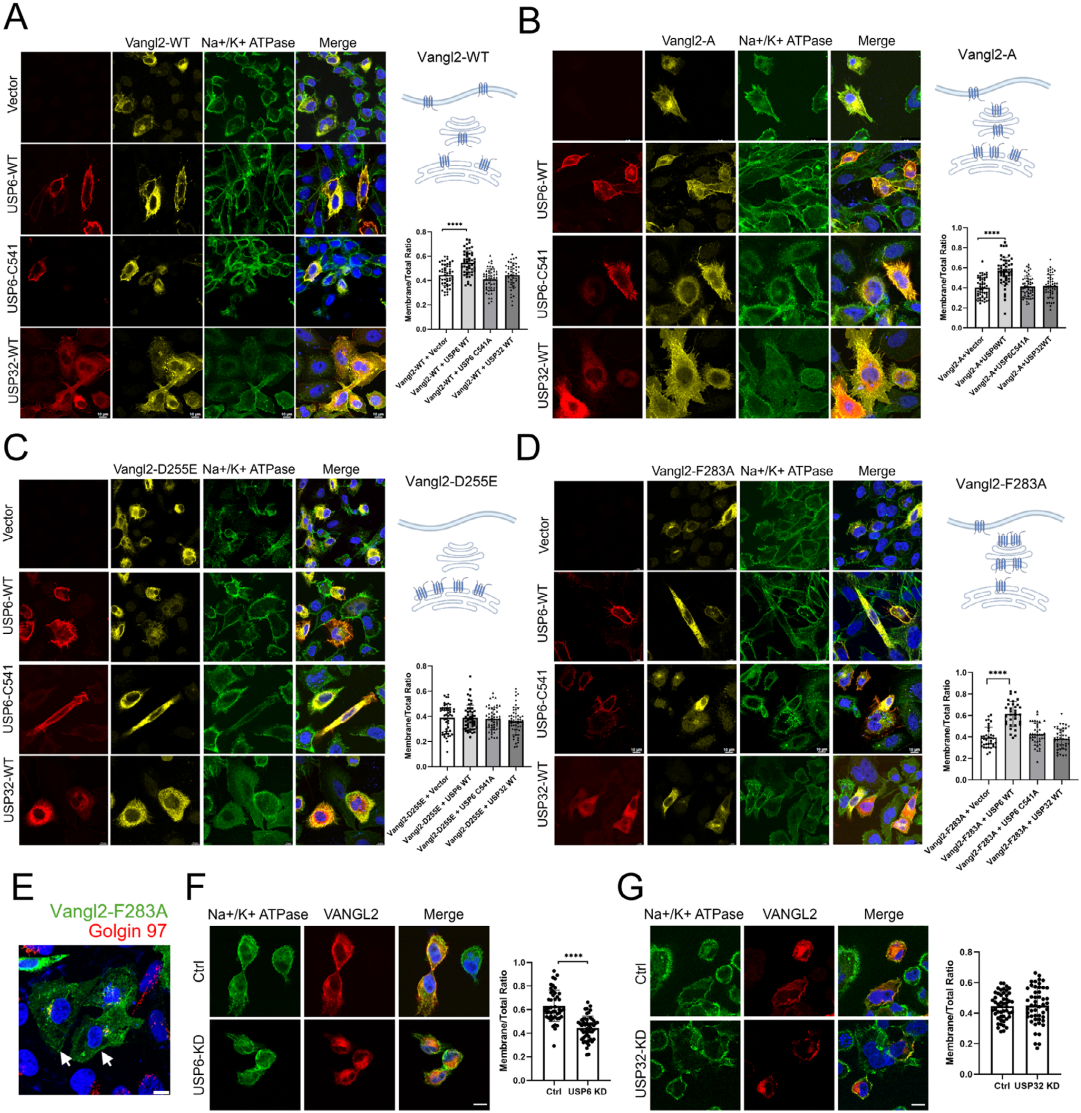
USP6 and USP32 differentially regulate the subcellular localization of Vangl. (A–D) Subcellular localization of Vangl2 variants in CHO cells co‐expressing USP6 wild‐type, USP6‐C541A, or USP32. Vangl2‐WT (A); phosphorylation‐deficient Vangl2‐A (B); ER‐trapped loop‐tail mutant Vangl2‐D255E (C); Golgi‐retained Vangl2‐F283A (D) USP6 increased the plasma membrane abundance of Vangl2‐WT, Vangl2‐A, and Vangl2‐F283A, but had little effect on Vangl2‐D255E. USP32 did not measurably alter the subcellular localization of these Vangl2 variants. Schematic distribution of Vangl2 variants and quantification of plasma membrane ratio under different co‐transfection conditions in more than 50 cells per group are shown in the right panels. (E) Subcellular localization of Vangl2‐F283A in CHO cells, showing a small but detectable fraction at the cell surface (arrows). (F) Subcellular localization of Vangl2‐WT in control and USP6‐depleted (USP6‐KD) HeLa cells, with quantification of plasma membrane ratio in the right panel. (G) Subcellular localization of Vangl2‐WT in control and USP32‐depleted (USP32‐KD) CHO cells, with quantification of plasma membrane ratio in the right panel. One‐way ANOVA test, ^****^ *p* <0.0001; Student's *t*‐test, ^****^ *p* < 0.0001. Scale bar = 10 um.

USP6 has been reported to stabilize Fzd5, a receptor in canonical Wnt/β‐catenin signaling, by increasing its membrane abundance [46]. We therefore asked whether USP6 similarly regulates the membrane abundance of Vangl. Na^+^/K^+^ ATPase was used as a plasma membrane marker for quantitative analysis (Figure S4A). We found that USP6 increased the plasma membrane localization of Vangl2‐WT and a phosphorylation‐deficient Vangl2 (Vangl2‐A, trafficking‐inefficient mutant) [20, 27, 31], whereas the catalytic mutant USP6‐C541A had no effect (Figure 3A,B). As expected, plasma membrane‐localized USP6 did not alter the ER‐trapped *loop tail (Lp)* mutant Vangl2‐D255E [47] (Figure 3C). Unexpectedly, although Vangl2‐F283A has been reported to be retained in the Golgi apparatus [48], USP6 markedly increased its plasma membrane abundance (Figure 3D). Careful examination of Vangl2‐F283A localization revealed a small but detectable fraction at the cell surface (Figure 3E), indicating partial leakage to the plasma membrane. These data suggest that this membrane‐accessible fraction of VANGL2‐F283A can be stabilized by USP6. In contrast, USP32 did not alter the plasma membrane localization of Vangl2‐WT or any of these variants (Figure 3A–D), suggesting that USP32‐mediated removal of K48‐linked ubiquitin chains at the Golgi/ER does not promote Vangl trafficking. We further knocked down USP6 or USP32 and examined Vangl2 localization. Depletion of USP6 reduced the plasma membrane localization of Vangl2, consistent with a role for USP6 in stabilizing the membrane‐associated Vangl2 pool (Figure 3F). In contrast, USP32 depletion reduced the overall Vangl2 protein level but did not significantly change the membrane localization ratio, supporting the idea that USP32 mainly stabilizes an intracellular Vangl pool rather than directly promoting membrane delivery (Figure 3G).

**FIGURE 4:**
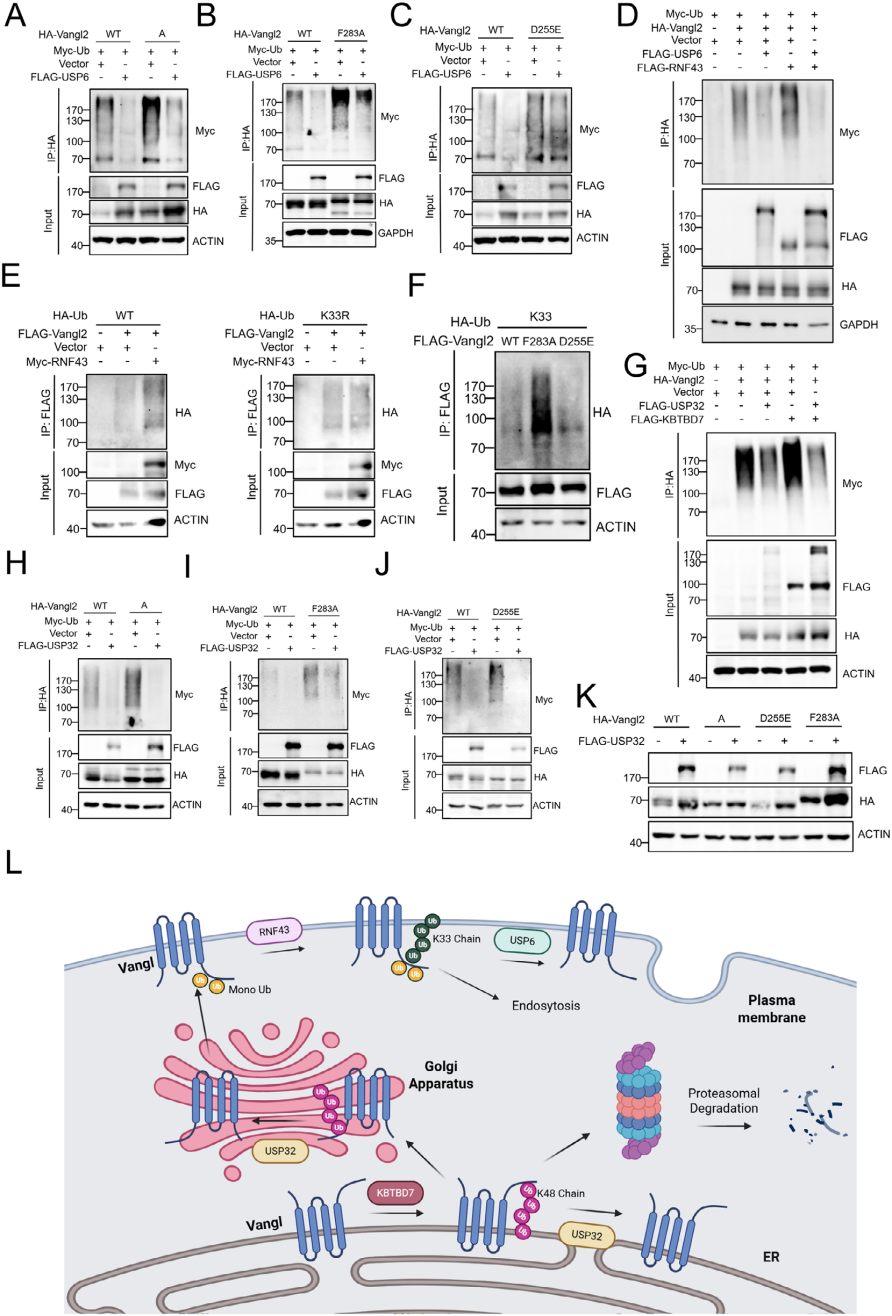
USP6 and USP32 antagonize distinct E3 ligases to deubiquitinate different Vangl pools. (A–C) Ubiquitination analysis of Vangl2 variants in the presence or absence of USP6 in HEK293T cells. (D) USP6 antagonized RNF43‐mediated ubiquitination of Vangl2. (E) RNF43‐mediated ubiquitination of Vangl2 predominantly involved K33‐linked ubiquitin chains. (F) Vangl2‐F283A mutant ubiquitinated by K33‐linked ubiquitin chains. (G) USP32 antagonized KBTBD7‐mediated ubiquitination of Vangl2. (H–J) Ubiquitination analysis of Vangl2 variants in the presence or absence of USP32. (K) Protein levels of Vangl2 variants in the presence or absence of USP32. (L) Working model: Vangl is ubiquitinated by CUL3‐KBTBD7 with K48‐linked ubiquitin chains in the ER and counteracted by USP32 at the Golgi/ER. A membrane‐accessible pool of Vangl is poly‐ubiquitinated by RNF43 through K33‐linked chains and multi‐mono‐ubiquitinated at the cell surface and deubiquitinated by USP6, thereby stabilizing plasma membrane‐localized Vangl. The original blot can be found in Figure S16.

These observations prompted us to examine whether the effects of USP6 on Vangl localization correlate with changes in Vangl ubiquitination. Expression of USP6 markedly reduced the polyubiquitination of Vangl2‐A and Vangl2‐F283A (Figure 4A,B). In contrast, the ER‐trapped Vangl2‐D255E mutant was largely unaffected by USP6 (Figure 4C). Notably, all of these Vangl2 variants exhibited substantially higher levels of ubiquitination than Vangl2‐WT (Figure 4A–C). The elevated ubiquitination of Vangl2‐F283A was only partially reduced by USP6 (Figure 4B), consistent with the limited accessibility of USP6 to the Golgi‐confined pool of Vangl2‐F283A.

Vangl proteins have been reported to be regulated by E3 ubiquitin ligases CUL3‐KBTBD7 and RNF43, which act at the ER and plasma membrane, respectively [31, 38]. Interestingly, USP6 antagonized the activity of RNF43 by significantly reducing RNF43‐induced ubiquitination of Vangl2 (Figure 4D). We further verified that RNF43‐mediated ubiquitination of Vangl2 predominantly involves K33‐linked ubiquitin chains, which can be efficiently removed by USP6 (Figure 4D,E). When we examined compartment‐biased Vangl2 variants, the ER‐retained Vangl2‐D255E mutant showed little K33‐linked ubiquitination, whereas the Golgi‐enriched Vangl2‐F283A mutant showed strong K33‐linked ubiquitination (Figure 4F). These data suggest that K33‐linked ubiquitination of Vangl2 mainly occurs in the Golgi or post‐Golgi compartment. Because USP6 is predominantly localized at the plasma membrane, it likely removes K33‐linked ubiquitin chains after Vangl2 reaches the cell surface. In contrast, USP32 antagonized KBTBD7‐mediated ubiquitination (Figure 4G), which is associated mainly with K48‐linked ubiquitin chains in the ER [31]. Consistent with this model, Golgi/ER‐localized USP32 reduced the ubiquitination of all Vangl2 variants and concomitantly increased their protein levels (Figure 4H–K). We further examined degradation routes associated with USP6 and USP32 depletion. Inhibition of proteasomal degradation by MG132 or ER‐associated degradation (ERAD) by NMS873 rescued the reduction of Vangl2 caused by USP32 depletion, whereas lysosome inhibition by chloroquine (CQ) partially rescued the reduction of Vangl2 caused by USP6 depletion (Figure S3). Together, these results indicate that USP6 and USP32 regulate distinct Vangl pools through linkage‐ and compartment‐specific deubiquitination (Figure 4L).

### 2.4 USP32 Genetically Interacts with Vangl1 and Vangl2 to Regulate PCP Signaling

To determine whether deubiquitination‐mediated regulation of Vangl contributes to PCP signaling in vivo, we next examined genetic interactions between these DUBs and Vangl in mice. Although both USP6 and USP32 regulate Vangl stability in cultured cells, USP6 is not present in rodents, whereas USP32 is evolutionarily conserved. We therefore focused on USP32 for in vivo genetic analysis. We generated *Usp32* mutant mice and found that *Usp32* null embryos were early embryonically lethal, indicating an essential developmental role for *Usp32*. Because PCP signaling is highly sensitive to gene dosage, we therefore examined whether partial reduction of *Usp32* genetically interacts with Vangl mutations.

**FIGURE 5:**
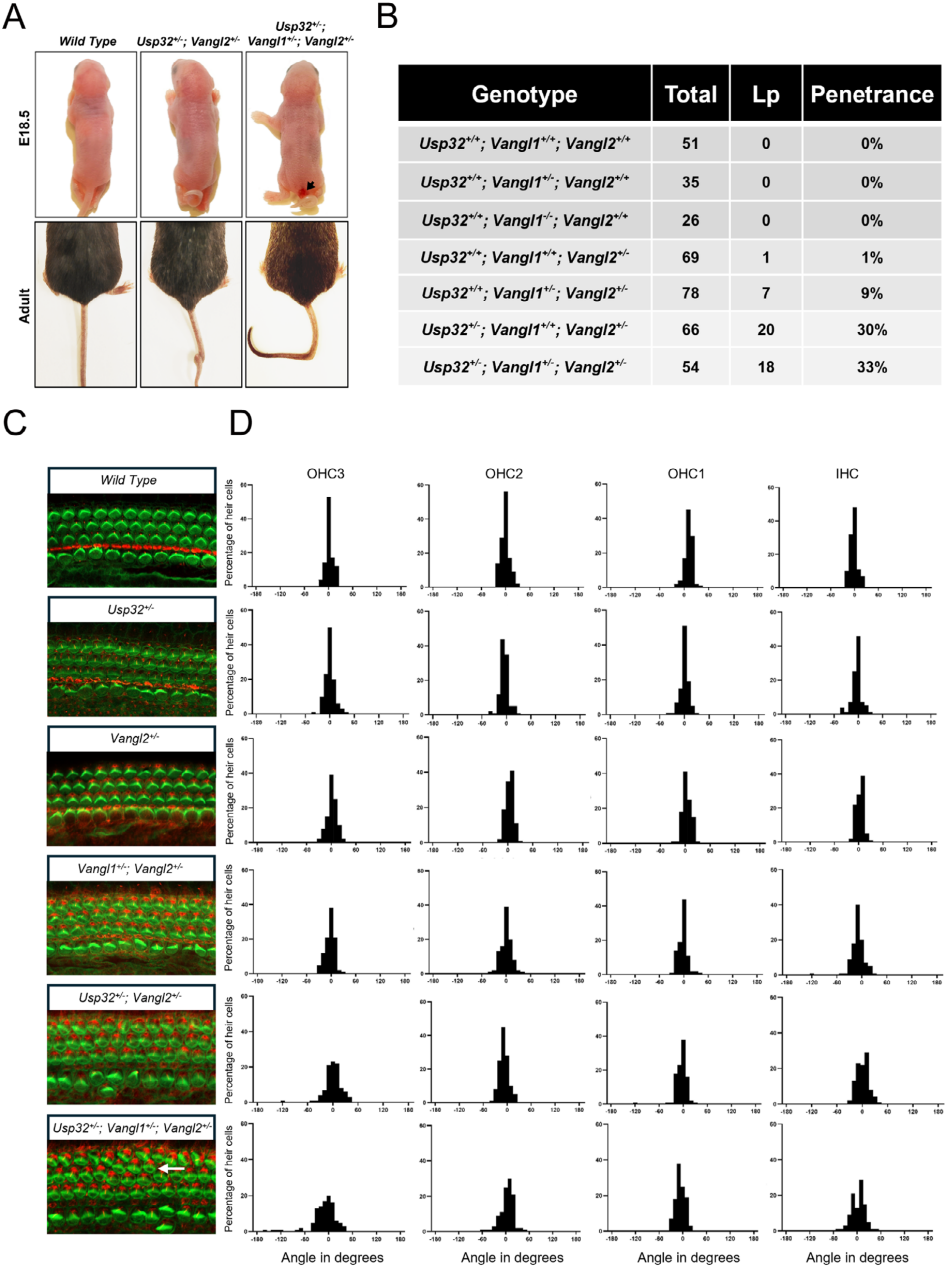
USP32 genetically interacts with Vangl to regulate PCP signaling. (A) Representative images of mice with the indicated genotypes. Upper panel, E18.5 embryos; lower panel, adult mice. The black arrow points to spina bifida. (B) The loop tail penetrance in the indicated genotypes. (C,D) PCP defects in the cochlea of compound mutant mice. (C) Confocal images of cochlear hair cells from E18.5 mouse embryos with the indicated genotypes. Hair bundles were labeled with phalloidin (green) and acetylated tubulin (red). Images were collected from the basal region of the cochlea. A white arrow points to an ectopic layer of hair cells. (D) Quantification of stereociliary bundle orientation angles in inner hair cells (IHC) and outer hair cells (OHC1, OHC2, and OHC3).

Consistent with previous reports, *Vangl2^+/−^ and Vangl1^+/−^;Vangl2^+/−^* mice displayed very low penetrance of PCP defects, with loop‐tail phenotypes observed in only ∼1% and ∼9% of mice, respectively (Figure 5A,B). This phenotype was not present in *Vangl1^+/−^* or *Vangl1^−/−^* mice, whereas *Vangl2^−/−^* embryos were lethal and showed complete open neural tube defects [10, 27]. Loss of a single *Usp32* allele markedly increased the penetrance of PCP defects, raising the frequency of loop‐tail to ∼30% in *Usp32^+/−^;Vangl2^+/−^* and ∼33% in *Usp32^+/−^;Vangl1^+/−^; Vangl2^+/−^* mice (Figure 5A,B). Moreover, while *Usp32^+/−^;Vangl2^+/−^* primarily exhibited loop‐tail, the triple heterozygous *Usp32^+/−^;Vangl1^+/−^;Vangl2^+/−^* frequently developed spina bifida (arrow in Figure 5A), indicating a more severe disruption of PCP signaling.

To further assess PCP signaling, we analyzed stereociliary bundle orientation in cochlear hair cells, a well‐established readout of PCP signaling. In control cochleae, stereociliary bundles of both inner hair cells (IHCs) and outer hair cells (OHCs) were uniformly aligned along the mediolateral axis of the organ of Corti. While *Usp32^+/−^*, *Vangl2^+/−^*, and *Vangl1^+/−^;Vangl2^+/−^* cochleae displayed normal hair bundle orientation, compound *Usp32^+/−^*;*Vangl* mutant cochleae showed pronounced polarity defects, including misoriented stereociliary bundles and disrupted alignment across the sensory epithelium (Figure 5C). Quantification confirmed a significant increase in hair bundle misorientation in the compound mutants compared with single mutants or controls (Figure 5D; Figure S4B). Together, these results demonstrate that *Usp32* genetically interacts with Vangl genes in a dosage‐dependent manner to regulate PCP signaling.

### 2.5 USP32 and VANGL1 Are Strongly Upregulated in Pancreatic Cancer

**FIGURE 6:**
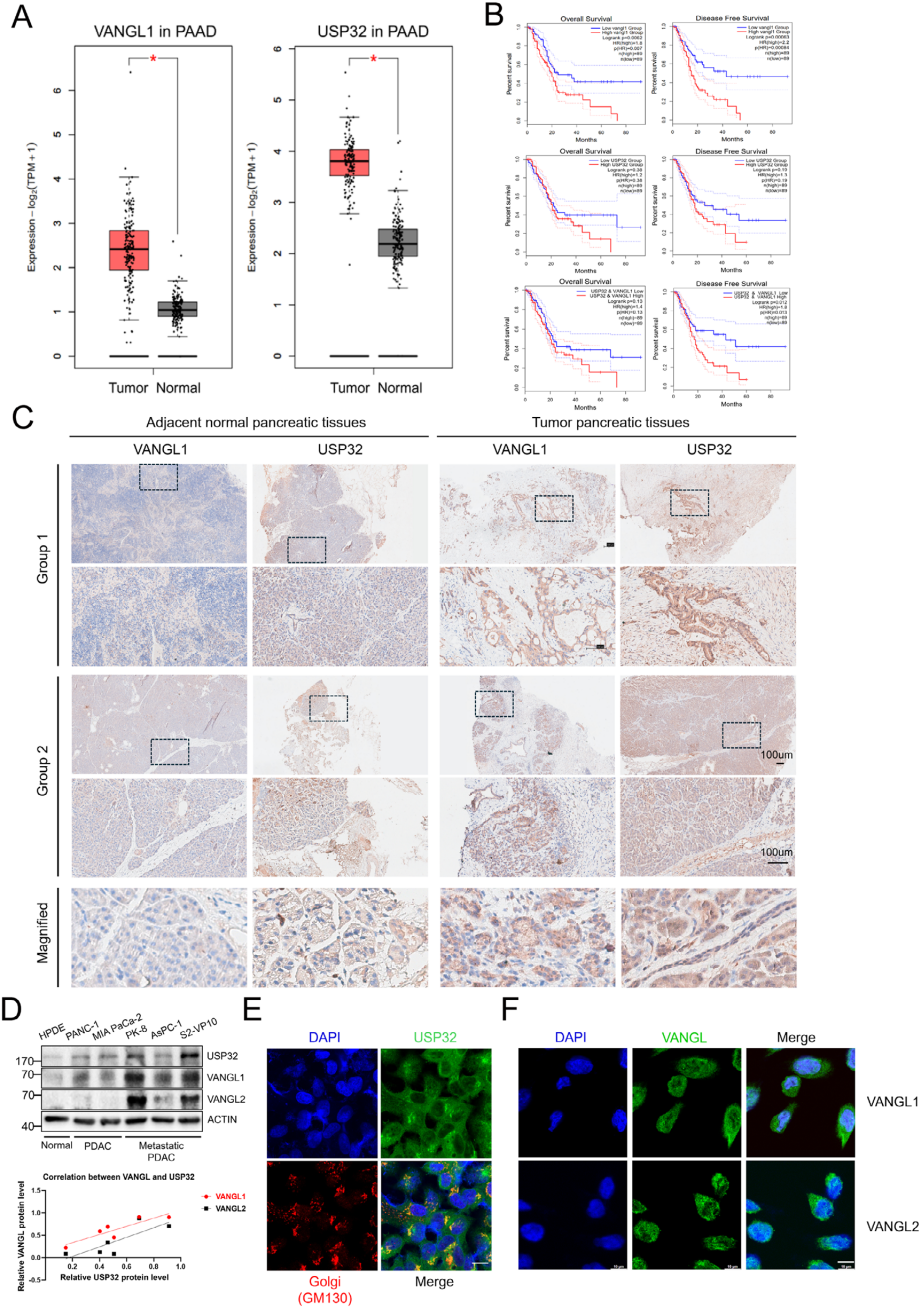
VANGL1 and USP32 are strongly upregulated in pancreatic cancer. (A) Transcript levels of *VANGL1* and *USP32* in pancreatic adenocarcinoma (PAAD) and normal pancreas, analyzed by GEPIA2 using TCGA and GTEx datasets. (B) Kaplan‐Meier analysis of overall survival and disease‐free survival in PAAD patients stratified by *USP32*, *VANGL1*, or combined expression. (C) Immunohistochemical staining of VANGL1 and USP32 in human pancreatic tumor samples and adjacent normal pancreatic tissues. Scale bar = 100 um. (D) Protein expression of USP32, VANGL1, and VANGL2 in a panel of pancreatic ductal adenocarcinoma (PDAC) cell lines and in non‐transformed HPDE cells. Lower panel, relative quantification and correlation analysis. (E) Subcellular localization of USP32 in S2‐VP10 PDAC cells. USP32 was enriched in the Golgi (GM130). (F) Subcellular localization of VANGL1 and VANGL2 in S2‐VP10 PDAC cells. Student's *t*‐test, ^*^ *p* < 0.05. Scale bar = 10 um. The original blot can be found in Figure S16.

Because PCP components have been implicated in cancer progression, we next investigated whether the USP32‐VANGL regulatory axis is altered in cancer. Analysis of publicly available transcriptomic datasets (TCGA and GTEx) revealed that *USP32* and *VANGL1* expressions are significantly elevated in pancreatic adenocarcinoma (PAAD) compared with normal pancreatic tissues (Figure 6A; Figure S5). Kaplan‐Meier survival analysis further showed that higher expression of *USP32* or *VANGL1* correlates with poorer overall and disease‐free survival in PAAD patients (Figure 6B), suggesting a potential role for this pathway in pancreatic cancer progression. We further analyzed combined expression of *USP32* and *VANGL1*, and high combined expression was also significantly associated with poorer patient survival (Figure 6B). Univariate and multivariate Cox proportional hazards regression analyses using available clinicopathological variables showed that high *VANGL1* expression remained significantly associated with poor survival (Figure S6).

To validate these observations experimentally, we examined USP32 and VANGL1 protein expression in pancreatic cancer tissues and cell lines. Immunohistochemical staining of human pancreatic tumor samples and adjacent normal pancreatic tissues revealed strong enrichment of USP32 and VANGL1 in tumor cells, particularly in pancreatic ducts of PAAD patients, whereas their expression was relatively low in normal pancreatic ducts (Figure 6C). We also performed VANGL2 staining in pancreatic tumor samples. Interestingly, although transcriptomic datasets did not show a significant increase in VANGL2 mRNA expression, VANGL2 protein levels were elevated in tumor tissues compared with adjacent normal tissues (Figure S7). To further examine USP32 and VANGL1/2 expression in pancreatic cancer cells, we analyzed a panel of pancreatic ductal adenocarcinoma (PDAC) cell lines and compared them with a non‐transformed human pancreatic duct epithelial (HPDE) cell line. Among these, PANC‐1 and MIA PaCa‐2 are primary tumor‐derived PDAC cell lines, while PK‐8, AsPC‐1, and S2‐VP10 are metastatic PDAC cell lines derived from the liver or ascites of pancreatic cancer patients. Immunoblot analysis revealed that USP32 and VANGL1/2 protein levels were markedly increased in multiple PDAC cell lines compared with HPDE cells (Figure 6D). Notably, all metastatic PDAC cell lines exhibited particularly higher levels of USP32 and VANGL1/2 than primary tumor‐derived PDAC cell lines. Interestingly, USP32 and VANGL1/2 protein levels are highly correlated across these cell lines (Figure 6D). Consistent with our earlier observations, USP32 is also enriched in the Golgi apparatus in S2‐VP10 cells (Figure 6E). Notably, VANGL1 and VANGL2 were mainly localized in the cytoplasm of tumor cells in both human samples and the S2‐VP10 cell line (Figure 6C,F), in line with our earlier result that USP32 primarily regulates the stability of an intracellular Vangl pool rather than enhancing membrane trafficking (Figure 3A–D). Together, these findings demonstrate that USP32 and VANGL protein levels are upregulated in PDAC, supporting a potential role for USP32‐mediated stabilization of VANGL proteins in pancreatic cancer.

### 2.6 VANGL1 Promotes PDAC Cell Migration and Metastasis

**FIGURE 7:**
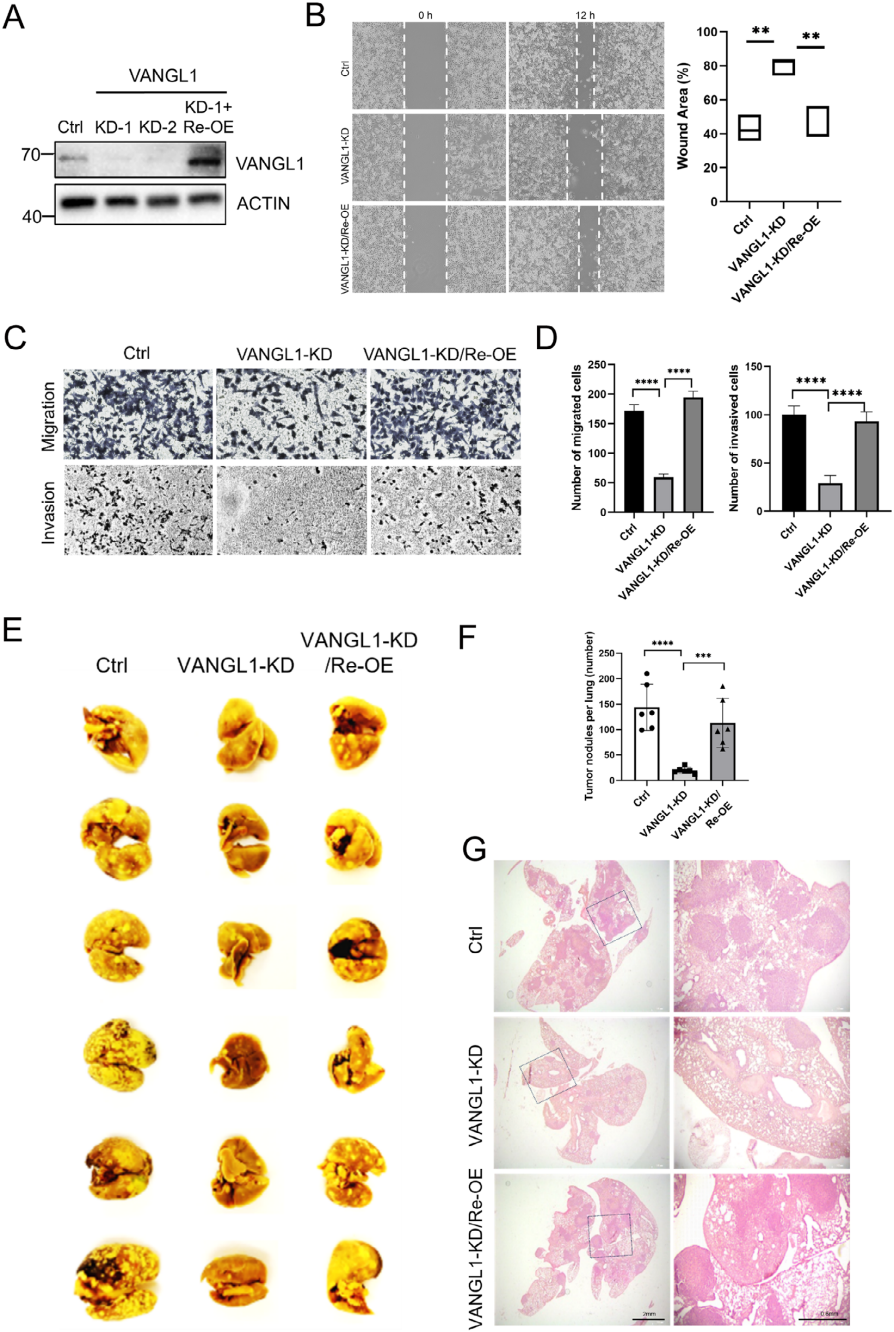
VANGL1 promotes PDAC cell migration and metastasis. (A) Generation of stable S2‐VP10 cell lines with VANGL1 knockdown (KD) and VANGL1 re‐expression (Re‐OE). (B) Wound healing assays in control, VANGL1‐knockdown (KD), and VANGL1 re‐expression (Re‐OE) S2‐VP10 cell lines. (C,D) Transwell migration and Matrigel invasion assays of the indicated S2‐VP10 cell lines and their quantification. (E) Tail vein injection lung metastasis assay using the indicated S2‐VP10 cell lines in NOD‐SCID mice. (F) Quantification of metastatic lung nodules in the tail vein injection assay. (G) Representative H&E staining of lung sections from mice injected with the indicated S2‐VP10 cell lines. Student's *t*‐test, ^**^ *p* < 0.01, ^***^ *p* < 0.001, ^****^ *p* < 0.0001. Scale bar = 2 mm. The original blot can be found in Figure S16.

Given the elevated expression of USP32 and VANGL1 in pancreatic cancer, we first examined the functional role of VANGL1 in PDAC cells. Using the metastatic PDAC cell line S2‐VP10, which expresses high levels of VANGL1, we generated stable VANGL1 knockdown cells using two independent shRNAs, and efficient depletion was confirmed by immunoblotting (Figure 7A). VANGL1 knockdown had minimal effects on cell proliferation as assessed by growth and colony formation assays (Figure S8), indicating that VANGL1 is not required for PDAC cell growth. In contrast, depletion of VANGL1 markedly impaired PDAC cell motility. In wound‐healing assays, VANGL1 knockdown significantly delayed gap closure compared with control cells (Figure 7B). Consistently, transwell migration and Matrigel invasion assays revealed a substantial reduction in migrating and invading cells following VANGL1 depletion (Figure 7C,D). Importantly, re‐expression of VANGL1 in knockdown cells restored their migratory and invasive capacities, confirming that the observed defects were specifically caused by loss of VANGL1 (Figure 7B–D). These effects of VANGL1 were further validated in PK‐8 cells, an additional PDAC cell line with high VANGL1 expression (Figure 6D; Figure S9). Moreover, depletion of VANGL2 in S2‐VP10 cells similarly had minimal effects on cell proliferation but significantly reduced cell migration and invasion (Figure S10), suggesting that both VANGL proteins contribute to the motile and invasive properties of PDAC cells.

To evaluate whether VANGL1 also contributes to metastatic potential in vivo, S2‐VP10 cells with VANGL1 knockdown or VANGL1 re‐expression were inoculated into NOD‐SCID mice via tail vein injection. Consistent with the in vitro results, VANGL1 depletion significantly reduced lung metastatic colonization compared with control cells, whereas re‐expression of VANGL1 restored metastatic capacity (Figure 7E‐G). These results demonstrate that VANGL1 promotes PDAC cell migration and metastasis.

### 2.7 USP32 Promotes PDAC Migration and Metastasis Through Stabilization of VANGL1

Having established that VANGL1 promotes PDAC cell migration and metastasis, we next investigated whether USP32 regulates these processes through stabilization of VANGL1. To this end, we manipulated USP32 expression in two PDAC cell lines with different endogenous USP32 levels. The metastatic PDAC cell line S2‐VP10, which expresses relatively high levels of USP32, was used for USP32 knockdown, whereas MIA PaCa‐2, which expresses lower levels of USP32, was used for USP32 overexpression.

**FIGURE 8:**
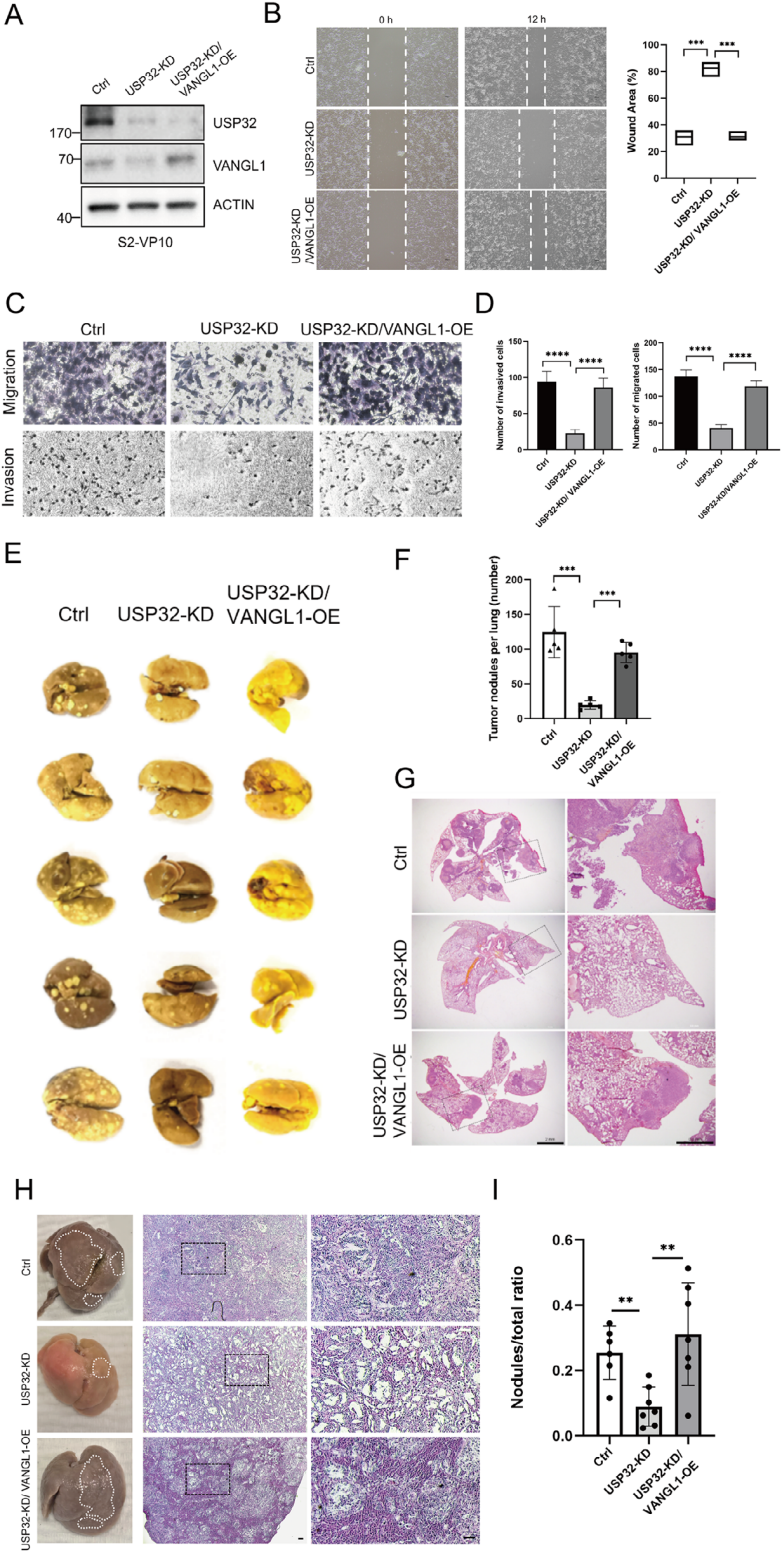
USP32 promotes PDAC migration and metastasis through stabilization of VANGL1. (A) Generation and validation of stable S2‐VP10 cell lines with USP32 knockdown (USP32‐KD) and VANGL1 re‐expression (VANGL1‐OE). (B) Wound healing assay of control, USP32‐KD, and USP32‐KD plus VANGL1‐OE S2‐VP10 cells, with quantification. (C,D) Transwell migration and Matrigel invasion assays of the indicated S2‐VP10 cell lines and their quantification. (E) Tail vein injection lung metastasis assay using control, USP32‐KD, and USP32‐KD plus VANGL1‐OE S2‐VP10 cells in NOD‐SCID mice. (F) Quantification of lung metastatic burden in the tail vein injection assay. (G) Representative H&E staining of lung metastatic lesions from the indicated groups. Scale bar = 2 mm (H) Splenic injection assay to evaluate liver metastasis of the indicated S2‐VP10 cell lines. Representative gross liver images and histological analysis are shown. Scale bar = 100 um. I) Quantification of liver metastatic burden in the indicated groups. Student's *t*‐test, ^**^ *p* < 0.01, ^***^ *p* < 0.001, ^****^ *p* < 0.0001. The original blot can be found in Figure S16.

Efficient depletion of USP32 in S2‐VP10 cells reduced VANGL1 protein level, consistent with the role of USP32 in stabilizing VANGL proteins (Figure 8A). Conversely, ectopic expression of USP32 in MIA PaCa‐2 cells increased VANGL1 protein level (Figure S11A). Importantly, manipulation of USP32 expression in either cell line had minimal effects on cell proliferation, as assessed by cell growth and colony formation assays (Figures S11B,C and S12), indicating that USP32 does not influence PDAC cell growth. In contrast, USP32 strongly affected PDAC cell motility. In S2‐VP10 cells, USP32 knockdown significantly impaired migration and invasion, as demonstrated by delayed wound closure in wound‐healing assays and reduced migration and invasion in transwell and Matrigel invasion assays (Figure 8B–D). Importantly, re‐expression of VANGL1 largely restored the migratory and invasive capacities of USP32‐depleted cells, indicating that the motility defects were primarily caused by reduced VANGL1 protein levels (Figure 8B–D). Conversely, overexpression of USP32 in MIA PaCa‐2 cells enhanced PDAC cell migration and invasion, whereas knockdown of VANGL1 in USP32‐overexpressing cells largely suppressed these effects, demonstrating that USP32 promotes PDAC cell motility primarily through stabilization of VAGNL1 (Figure S13). Consistent with the similar role of VANGL1 and VANGL2, USP32 knockdown also reduced VANGL2 protein levels in S2‐VP10 cells, and VANGL2 re‐expression partially rescued the migration and invasion defects caused by USP32 depletion (Figure S14A–E). In contrast, VANGL2 expression was undetectable in MIA PaCa‐2 cells with or without USP32 overexpression, consistent with the low endogenous VANGL2 level in this cell line (Figure 6D; Figure S14F).

We next examined whether USP32 regulates metastatic potential in vivo. Control or USP32‐depleted S2‐VP10 cells were injected into NOD‐SCID mice via tail vein injection to evaluate lung colonization. USP32 knockdown significantly reduced lung metastatic burden compared with control cells. Importantly, re‐expression of VANGL1 restored the metastatic capacity of USP32‐depleted cells, consistent with the in vitro rescue results (Figure 8E–G). To further assess metastatic dissemination, we employed a splenic injection model, which allows tumor cells to enter the portal circulation and efficiently seed the liver, thereby providing a physiologically relevant assay for hepatic metastasis. Control or USP32‐depleted S2‐VP10 cells were injected into the spleen of NOD‐SCID mice, and liver metastases were examined 27 days after injection. Consistent with the lung colonization results, USP32 depletion markedly reduced liver metastatic burden, as evidenced by a substantial decrease in the number of metastatic nodules observed on the liver surface compared with control mice (Figure 8H,I). Histological examination of liver sections further confirmed a pronounced reduction in metastatic lesions in mice injected with USP32‐depleted cells (Figure 8H). Importantly, re‐expression of VANGL1 in USP32‐depleted cells restored liver metastatic colonization (Figure 8H,I), indicating that the metastatic defects caused by USP32 depletion are largely mediated through reduced VANGL1 protein levels. Together, these findings demonstrate that USP32 promotes PDAC cell migration and metastasis primarily through stabilization of VANGL1, consistent with our biochemical results showing that USP32 stabilizes VANGL proteins through deubiquitination.

### 2.8 USP32‐Vangl Signaling Regulates Pancreatic Duct Morphogenesis During Development

Given that USP32 and VANGL promote pancreatic cancer cell migration, we next examined whether USP32‐Vangl signaling also plays a role in pancreatic morphogenesis during normal development. Although *Usp32* null embryos are largely embryonic lethal, a small number survived to E15.5, a stage when pancreatic epithelial branching and ductal remodeling are actively occurring, allowing us to analyze pancreatic morphology. Histological examination revealed a markedly altered branching pattern in the E15.5 *Usp32^−/−^* pancreas. At this stage in wild‐type embryos, pancreatic ducts normally undergo a remodeling process that transforms an initially uniform epithelial tube into a hierarchical ductal network with progressively narrowed luminal structures [49]. In contrast, *Usp32^−/−^* pancreas failed to complete this remodeling process, displaying dilated terminal ducts with abnormally wide and open lumens. Notably, *Vangl1* and *Vangl2* double knockout embryos exhibit a highly similar pancreatic branching phenotype (Figure S15A), supporting the notion that the developmental phenotype observed in *Usp32* mutants is associated with disruption of Vangl‐mediated PCP signaling. To further assess whether USP32 regulates Vangl proteins in vivo, we examined their expression in pancreatic tissues and found that Vangl2 protein levels were significantly reduced in *Usp32* mutant pancreas in a dose‐dependent manner (Figure S15B). Immunostaining further showed that Vangl proteins are predominantly localized at cell junctions of pancreatic duct epithelial cells [50], whereas USP32 displayed a primarily cytoplasmic distribution (Figure S15C). Together, these observations suggest that USP32 regulates pancreatic duct morphogenesis by stabilizing Vangl proteins during pancreatic branching development.

## 3 Discussion

Our study identifies a previously missing layer of Vangl regulation in PCP signaling by showing that the core PCP proteins Vangl1 and Vangl2 are directly regulated by two deubiquitinases, USP6 and USP32. This finding reveals a dynamic PTM balance, in which phosphorylation, ubiquitination, and deubiquitination act together to define Vangl abundance and localization. Previous work has demonstrated that Wnt5a/CK1 promotes the phosphorylation of Vangl2, facilitates its ER‐to‐plasma membrane trafficking, and opposes ERAD‐mediated turnover, thereby critically influencing its PCP activity and membrane distribution [20, 27, 31]. In contrast, the ubiquitin‐proteasome system acts as a potent negative regulator, with E3 ubiquitin ligases such as CUL3‐KBTBD7 and RNF43 targeting Vangl for degradation, thereby finely tuning its cellular abundance [31, 38]. Our data now add another layer of Vangl regulation and show that Vangl dosage is set not only by degradation, but by continuous ubiquitin editing across distinct subcellular compartments. Importantly, Wnt5a did not alter USP6 or USP32 protein levels, suggesting that these DUBs are not simply induced by Wnt5a signaling, but instead may act as constitutive quality‐control or surveillance factors that edit ubiquitin marks on distinct Vangl pools. This is important because PCP signaling is dosage sensitive, and relatively modest changes in Vangl abundance are sufficient to alter developmental outcomes.

Although USP6 is evolutionarily derived from USP32, they do not act redundantly but instead regulate distinct Vangl pools through different ubiquitin linkages. Our results support a model in which USP6 primarily acts on plasma membrane‐accessible Vangl and antagonizes RNF43‐associated K33‐linked ubiquitination, as well as multi‐monoubiquitination, whereas USP32 acts predominantly on a Golgi/ER‐associated Vangl pool and removes K48‐linked ubiquitin chains (Figure 4L). This distinction is mechanistically compelling because K48‐linked chains are the canonical signal for proteasomal degradation, while K33‐linked chains are generally regarded as non‐canonical ubiquitin marks that participate in receptor signaling and membrane trafficking rather than serving as a simple degradation signal, and monoubiquitination commonly regulates cell‐surface transmembrane proteins during endocytosis and lysosomal turnover [51, 52, 53, 54]. In this context, the inability of USP6 to influence the ER‐trapped loop‐tail D255E mutant, together with its partial effect on the “Golgi‐retained” F283A mutant, strongly argues that USP6 requires access to a plasma membrane‐trafficked Vangl pool. By contrast, the lack of a clear trafficking effect for USP32, despite its robust K48 deubiquitination activity, suggests that USP32 primarily stabilizes Vangl during biosynthetic or early secretory quality control rather than directly increasing plasma membrane delivery. We therefore propose a model in which different ubiquitin chain types encode different fates for the same PCP cargo at different steps of its intracellular trafficking route.

USP32 has been reported to regulate multiple substrates and previously linked to endosomal transport and recycling through deubiquitination of trafficking regulators such as Rab7, Rab35, and LAMTOR1, consistent with a general role in membrane trafficking and cargo sorting [55, 56, 57, 58, 59]. Our data expand its role by identifying Vangl as a signaling cargo whose stability is shaped by USP32 at the Golgi/ER. USP6, in contrast, is a hominoid‐specific chimeric gene derived from a USP32 and TBC1D3 fusion and has previously been shown to increase Fzd5 cell‐surface abundance by deubiquitination [45, 46]. The fact that USP6 similarly enhances membrane‐accessible Vangl suggests that primate evolution may have added an additional membrane‐proximal layer of PCP regulation on top of the ancestral USP32 pathway. Because rodents lack USP6, the in vivo developmental contribution of USP6 cannot yet be addressed genetically in mouse models. Nevertheless, our biochemical data suggest that USP6 is not only a duplicate of USP32, but a specialized derivative that has adopted a more cell surface‐proximal mechanism of cargo regulation.

The physiological relevance of USP32‐mediated Vangl2 regulation is supported by our mouse genetic data. Although *Usp32* null embryos are largely early lethal, partial reduction of *Usp32* sensitized *Vangl* mutant backgrounds, leading to increased loop‐tail penetrance and the emergence of spina bifida in compound mutants. Together with the stereociliary bundle disorganization observed in the cochlea, these findings establish *Usp32* as an in vivo modulator of Vangl dosage during PCP signaling. This is reinforced by the pancreatic phenotype. The *Usp32* null embryos showed defective pancreatic duct remodeling, resembling the phenotype of Vangl‐deficient pancreas. The dose‐dependent reduction of Vangl2 protein in *Usp32* mutant pancreatic tissue further supports the conclusion that USP32 regulates Vangl‐dependent morphogenesis in vivo.

USP6 and USP32 have both been implicated in tumorigenesis in multiple malignancies. USP6 is a well‐established oncogene that is recurrently translocated and overexpressed in ∼70% and 90% of patients diagnosed with aneurysmal bone cyst and nodular fasciitis, respectively [60], whereas USP32 upregulation has been linked to oncogenic behavior in many cancer types [55, 61, 62, 63, 64, 65, 66, 67, 68, 69, 70, 71]. In this context, our finding that USP32 and VANGL1 are coordinately upregulated in PDAC, and that their expression correlates with poor patient outcome, suggests that the USP32‐VANGL1 axis represents a clinically relevant branch of PCP signaling in pancreatic cancer. Importantly, our study provides a mechanistic explanation for how USP32 contributes to pancreatic malignancy. We found that USP32 promotes PDAC cell migration, invasion, and metastatic colonization both in vitro and in vivo, while having no effect on proliferation, indicating that its major function is to drive metastatic behavior rather than general tumor growth. Mechanistically, this activity is mediated largely through VANGL1, because the motility and metastatic defects caused by USP32 depletion were rescued by re‐expression of VANGL1, whereas the pro‐migratory effects of USP32 overexpression were suppressed by VANGL1 knockdown. These findings place VANGL1 downstream of USP32 in PDAC and support the conclusion that USP32 promotes pancreatic cancer progression by stabilizing this core PCP protein. This model is further supported by recent studies showing that loss of RNF43, one of the E3 ligases targeting VANGL, accelerates PDAC progression [72], and that WNT5A, an upstream regulator of VANGL‐dependent PCP signaling, promotes pancreatic cancer cell migration and tumorigenesis [73]. Together, these observations suggest that altered VANGL regulation at multiple levels converges to enhance PCP‐associated migratory behaviors in PDAC.

While our analyses initially focused on VANGL1 because of its stronger clinical association in PDAC at the transcriptome level, VANGL2 protein was also elevated in pancreatic tumor tissues and in multiple metastatic PDAC cell lines, and VANGL2 affected S2‐VP10 migration and invasion. These results are consistent with the known redundant roles between VANGL1 and VANGL2 in development. Notably, both VANGL1 and VANGL2 were mainly cytoplasmic in pancreatic tumor tissues and S2‐VP10 PDAC cells, rather than being clearly enriched at cell junctions. This cytoplasmic localization is consistent with our biochemical model that USP32 stabilizes an intracellular Vangl pool and suggests that altered intracellular accumulation of VANGL proteins may support PDAC cell motility, although the underlying mechanism remains to be elucidated.

In summary, our study establishes deubiquitination as a critical regulatory mechanism in PCP signaling by identifying USP6 and USP32 as two Vangl deubiquitinases with distinct compartmental and linkage specificities. We propose that Vangl dosage is controlled through a dynamic balance between phosphorylation, ubiquitination, and deubiquitination to maintain signaling output within a physiological range. By linking this mechanism to mouse PCP phenotypes, pancreatic duct morphogenesis, and PDAC metastasis, our study broadens the current understanding of PCP regulation and highlights USP32‐dependent stabilization of VANGL proteins in PDAC as a potential point of therapeutic intervention in pancreatic cancer.

## 4 Materials and Methods

### 4.1 CRISPR Library Screening for DUBs

Sanger CRISPR Library for DUBs was purchased from Sigma‐Aldrich, targeting ∼100 *DUB* genes of the human genome with 2 independent sgRNAs for each DUB. The Sanger lentiviral sgRNA vectors were derived from the LV04 U6‐gRNA:hPGK‐puro‐2A‐tBFP backbone. For screening of DUBs regulating Vangl proteins, HEK293T cells were co‐transfected with Sanger lentiviral sgRNA vectors, the 2nd‐generation lentiviral packaging plasmid psPAX2, and the envelope plasmid pMD2.G at a ratio of 5:4:1. The medium containing sgRNA‐expressing lentivirus was collected at 48 h and 72 h post‐transfection and combined. Newly seeded HEK293T cells were transduced with individual media for 48 h prior to selection with puromycin. DUB KO cell lines were cultured, expanded, and lysate supernatants were collected for immunoblot analysis of endogenous Vangl1 and Vangl2. Candidate DUBs were selected with the highest downregulation of endogenous Vangl protein levels.

### 4.2 Cell Culture and Plasmids

HEK293T, HeLa and CHO cells, as well as a series of human pancreatic ductal adenocarcinoma (PDAC) cell lines (PANC‐1, MIA PaCa‐2, PK‐8, AsPC‐1, S2‐VP10) were cultured in Dulbecco's Modified Eagle's Medium (DMEM; Thermo Fisher Scientific, 12‐800‐017) supplemented with 10% fetal bovine serum (FBS; Thermo Fisher Scientific, 10‐099‐141) and 1% penicillin‐streptomycin (Fisher Scientific, 15‐140‐163). Immortalized human pancreatic ductal epithelial (HPDE) cells were maintained in Keratinocyte Serum‐Free Medium (Thermo Scientific, 17005042) supplemented with epidermal growth factor (EGF) and bovine pituitary extract. All cell lines were incubated in a humidified atmosphere at 37 °C with 5% CO_2_. Plasmid construction was performed using standard recombinant DNA methodologies. Site‐directed mutagenesis was implemented by PCR amplification coupled with DpnI (NEB, R0176L) digestion. pRK5‐HA‐Ubiquitin‐K6R, 11R, 27R, 29R, 33R, 48R, 63R plasmids and pCMV‐3×FLAG‐RNF43 were purchased from MiaoLing Biology (P51117, P51090, P51094, P51093, P51111, P51109, P51110, and P57356).

### 4.3 Stable Cell Line Establishment and Transfection

Lentiviral transduction was employed to generate stable cell lines with targeted gene knockdown or overexpression. Short hairpin RNA (shRNA) cassettes were constructed and inserted into PLKO.1 or PLV2 lentiviral vectors, followed by PEI‐mediated transfection into HEK293T cells to produce infectious lentiviral particles. Target PDAC cell lines were then infected with concentrated lentiviruses, and stable transfectants were isolated via puromycin (Invitrogen, ant‐pr‐1) antibiotic selection.

### 4.4 Reagents and Antibodies

Major reagents applied in this work include various restriction endonucleases (NEB), proteasome inhibitor MG‐132 (Millipore, 474790), puromycin (Invitrogen, ant‐pr‐1), XTT detection kit (GoldBio, X‐200‐100), polyethylenimine (Sigma, 765090–1G), and ER staining kit (Abcam; ab139482). Primary antibodies were used at indicated dilutions: anti‐HA‐tag (ABclonal, AE008; Cell Signaling Technology, 3724S; 1:5000 for WB, 1:1000 for IF), anti‐FLAG‐tag (Sigma‐Aldrich, F3165‐1MG; 1:5000), anti‐MYC‐tag (Sigma‐Aldrich, M4439; 1:5000 for WB), anti‐Vangl1 (Sigma‐Aldrich, HPA025235; 1:1000), anti‐Vangl2 (R&D Systems; AF4815), anti‐USP6 (MyBioSource, MBS8519005; 1:1000), anti‐USP32 (Santa Cruz, sc‐374465; 1:1000 for WB, 1:100 for IF), anti‐GM130 (Abcam, ab52649), anti‐β‐actin (ABclonal, AC026; 1:5000 for WB), anti‐GAPDH (ABclonal, AC002; 1:5000 for WB). Horseradish peroxidase (HRP)‐conjugated secondary antibodies (Millipore; 1:5000) and Alexa Fluor‐conjugated secondary antibodies (Invitrogen; 1:1000 for IF) were utilized for protein detection.

### 4.5 Immunoblotting

Cells were lysed in RIPA buffer (150 mm NaCl, 10 mm Tris, 0.5% NP‐40, pH 7.4) supplemented with a protease inhibitor cocktail. Lysates were clarified by centrifugation at 13,000 × *g* for 15 min at 4°C. Equal amounts of protein (20‐30 µg) were resolved by 10% SDS‐PAGE and transferred onto PVDF membranes. Membranes were blocked with 5% bovine serum albumin (BSA), incubated with primary antibodies overnight at 4°C, and then probed with HRP‐conjugated secondary antibodies. Protein signals were visualized using SuperSignal West Femto Chemiluminescent Substrate (Thermo Fisher Scientific, 34096).

### 4.6 Immunoprecipitation and Ubiquitination Assays

Total cell lysates (500 µg) were incubated with 2 µg of specified antibodies overnight at 4°C and further incubated with protein A/G agarose beads (Santa Cruz Biotechnology, sc‐2003) for 2 h. Beads were washed three times with NP‐40 buffer, and immunoprecipitated proteins were eluted with Laemmli buffer for immunoblot analysis. Ubiquitination status was examined in transfected HEK293T cells. Cells were treated with 20 µm MG‐132 for 6 h before lysis in RIPA buffer containing 2% SDS, 10 mm NEM, and protease/phosphatase inhibitors. After boiling and brief sonication, lysates were centrifuged at 16,000 × *g* for 15 min at 4°C, and supernatants were diluted 10‐fold to lower SDS concentration. Immunoprecipitation was then conducted following the standard procedure. His‐ubiquitin pull‐down assays were conducted in HEK293T cells transfected with His‐ubiquitin and indicated plasmids for 48 h. Cells were lysed in ice‐cold Buffer A (6 m guanidine‐HCl, 0.1 M Na_2_HPO_4_/NaH_2_PO_4_, 10 mm imidazole, pH 8.0) with protease and phosphatase inhibitors. Lysates were incubated with pre‐equilibrated Ni‐NTA beads (QIAGEN, 30210) for 3 h at room temperature with gentle rotation. Beads were washed sequentially with Buffer A, Buffer B, and Buffer TI, and ubiquitinated proteins were detected by immunoblotting.

### 4.7 Immunofluorescence and Whole‐Mount Staining

Cells seeded on coverslips were transfected for 24 h, fixed in 4% paraformaldehyde (PFA) for 15 min, permeabilized with 0.5% Triton X‐100 for 10 min, and blocked with 3% BSA/PBS. Primary antibody incubation was carried out overnight at 4°C, followed by Alexa Fluor‐conjugated secondary antibodies for 1 h at room temperature. Nuclei were counterstained with DAPI (Fisher Scientific, P36970), and images were acquired using a Zeiss LSM 800 confocal microscope. Whole‐mount immunostaining was performed on E15.5 mouse embryonic pancreatic tissues. Freshly isolated pancreases were washed with cold PBS, fixed in 4% PFA, and dehydrated in a graded methanol series. Tissues were treated with an autofluorescence quenching solution and subjected to repeated freeze‐thaw cycles to enhance permeability. After rehydration, samples were blocked with CAS‐Block buffer (Thermo Scientific, 008120) and incubated with anti‐Muc1 antibody (Thermo Scientific, MA5‐11202;1:1000) for 48 h at 4°C, followed by secondary antibody incubation for another 48 h. Stained tissues were dehydrated, cleared in Benzyl Alcohol/Benzyl Benzoate (BABB) solution, and mounted for confocal imaging at 20× magnification.

Immunofluorescence micrographs of cells expressing Vangl2 were quantified with ImageJ software. Fluorescence intensities across the entire cell body and intracellular compartments were quantified separately. Membrane signal intensity was derived by subtracting intracellular intensity from the total cellular intensity, after which we calculated the fractional contributions of membrane relative to the overall cellular fluorescence as the membrane ratio (Figure S4A).

### 4.8 Human Clinical Specimens

Tumor tissues and adjacent non‐tumor tissues were obtained from pancreatic cancer patients undergoing surgical resection. All specimens were pathologically confirmed as pancreatic adenocarcinoma. Written informed consent was obtained from all participants prior to sample collection. Clinical and histopathological data were collected from medical records and pathological reports.

### 4.9 Animal Experiments

All animal protocols were reviewed and approved by the Centre for Comparative Medicine Research (CCMR) of the University of Hong Kong and strictly complied with ethical guidelines regulated by the Committee on the Use of Live Animals in Teaching and Research (CULATR). For in vivo tumor assays, 6‐8‐week‐old male and female NOD‐SCID mice were acquired from CCMR. *Usp32* null mice were generated via CRISPR‐Cas9‐mediated genome editing by Gempharmatech Company and imported to CCMR. The *Vangl1* and *Vangl2* mutant mouse strains have been described previously [10, 27].

### 4.10 Immunohistochemistry

Formalin‐fixed paraffin‐embedded (FFPE) tissue sections (4 µm) were processed on a BOND‐MAX Fully Automated IHC/ISH Staining System. Sections were incubated with anti‐USP32 and anti‐Vangl1 primary antibodies, followed by HRP‐conjugated secondary antibodies. Immunoreactivity was visualized with DAB chromogen, and nuclei were counterstained with hematoxylin. Images were captured using a conventional light microscope.

### 4.11 XTT Proliferation Assay

Cells were seeded into 96‐well plates at a density of 5 × 10^3^ cells per well. After 24 h, 50 µL XTT reagent (1 mg/mL XTT + 0.01 mm phenazine methosulfate) was added to each well. Following 2 h incubation at 37°C, absorbance at 450 nm was measured. Absorbance values were recorded daily for 5 consecutive days, and IC50 values were calculated using GraphPad Prism software.

### 4.12 Colony Formation Assay

Exponentially growing PDAC cells were trypsinized, counted, and plated at 200 cells per well in 6‐well plates. Cells were cultured for 14–21 days with medium replacement every 3 days. Colonies were fixed with 4% PFA, stained with 0.1% crystal violet, and air‐dried. Colonies containing ≥50 cells were counted manually under a light microscope.

### 4.13 Transwell Migration and Invasion Assays

Cell migration capacity was evaluated using Transwell inserts with 8‐µm pores (Corning, 3422). A total of 1 × 10^5^ cells resuspended in serum‐free medium were added to the upper chamber. For invasion assays, cells were pre‐seeded in the Matrigel invasion chambers (Corning, 354480). The lower chambers were filled with complete medium containing 10% FBS as a chemoattractant. After 24 h, migrated or invaded cells were fixed, stained with crystal violet, and counted in 10 random fields per insert.

### 4.14 Wound Healing Assay

Pancreatic cancer cells were seeded in 6‐well plates and cultured to 90–100% confluence. A linear scratch was created across the cell monolayer using a sterile 200 µL pipette tip. Floating cells were removed by PBS washing, and serum‐free medium was added to suppress proliferation‐dependent wound closure. Wound images were captured immediately (0 h) and at 12 h post‐scratching using an inverted microscope. The wound healing rate was calculated as: Wound healing rate (%) = [(Initial wound width – Final wound width) / Initial wound width].

### 4.15 Tail Vein Metastasis Model

Six‐ to eight‐week‐old NOD‐SCID mice were used for experimental metastasis assays. Cultured cells were trypsinized, washed with ice‐cold PBS, and resuspended in PBS for injection. Each mouse received 1.5 × 10^6^ cells via tail vein injection. Mice were euthanized for 28 days post‐injection, and lung tissues were dissected to quantify surface tumor nodules. Data were analyzed and visualized using GraphPad Prism.

### 4.16 Liver Metastasis Model

Liver metastasis was established by intrasplenic injection of PDAC cells into 6‐8‐week‐old NOD‐SCID mice. A total of 2 × 10^5^ cells in 100 µL PBS were inoculated per mouse. 27 days after injection, all mice were sacrificed by cervical dislocation, and liver tissues were harvested for histological examination.

### 4.17 Bioinformatic Analysis

Expression profiles and clinical correlations of VANGL1 and USP32 in pancreatic cancer were interrogated using the GEPIA platform, TCGA‐PAAD dataset, and GTEx database.

### 4.18 Statistical Analysis

Statistical evaluation was performed using SPSS 22.0 and GraphPad Prism 9.0 software. Comparisons between groups were conducted using Student's *t*‐test or one‐way analysis of variance (ANOVA). Data are presented as mean ± standard deviation (SD) from at least three independent experiments. A *p*‐value < 0.05 was considered statistically significant.

## Author Contributions

F.Z. performed the experiments, data analysis, and wrote the manuscript draft; D.F. performed the screening of DUBs, identified USP6 and USP32, and revised the manuscript; Z.X. and Y.L. contributed to the pathological analysis of pancreatic cancer samples; Z.L., X.L., and Z.W. assisted in the experiments; L.X. assisted in the revision of the manuscript. Y.G., K.M.C, J.F.W.C., M.C., and M.S.Y.H provided critical materials, reagents, and technical support for the study. F.Z., D.F., and B.G designed and conceptualized the study. B.G. supervised, reviewed, and edited the manuscript.

## Conflicts of Interest

The authors declare no conflicts of interest.

## Supporting information




**Supporting File**: advs77496‐sup‐0001‐S1‐S16.pdf.

## Data Availability

The data that support the findings of this study are available from the corresponding author upon reasonable request.
