# Supplementary material for: Deubiquitination of Vangl by USP6 and USP32 Regulates Planar Cell Polarity Signaling
Source: Adv Sci (Weinh). 2026 Aug 30:e77496. Online ahead of print. doi: 10.1002/advs.77496 (PMC13526532; doi:10.1002/advs.77496)
Supplement: Supplementary file 1 [file ADVS-9999-e77496-s001.pdf]

Figure S1

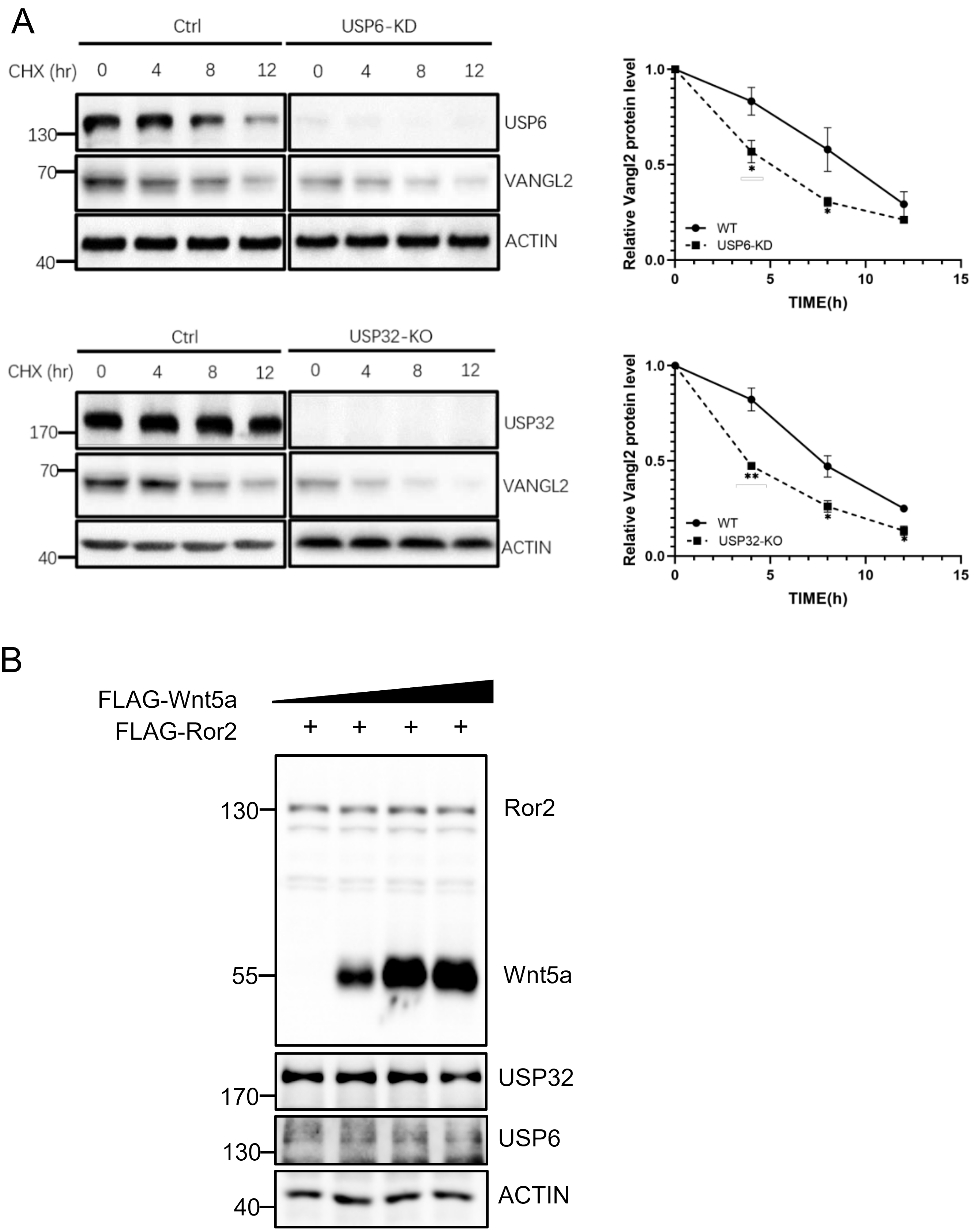

A
Ctrl
USP6-KD
CHX (hr)
0
4
8
12
0
4
8
12
USP6
VANGL2
ACTIN
130
70
40
Relative Vangl2 protein level
1.0
0.5
0.0
WT
USP6-KD
TIME(h)
0
5
10
15
Ctrl
USP32-KO
CHX (hr)
USP32
VANGL2
ACTIN
170
70
40
WT
USP32-KO
B
FLAG-Wnt5a
FLAG-Ror2
+
+
+
+
Ror2
Wnt5a
USP32
USP6
ACTIN
130
55
170
130
40

Figure S2

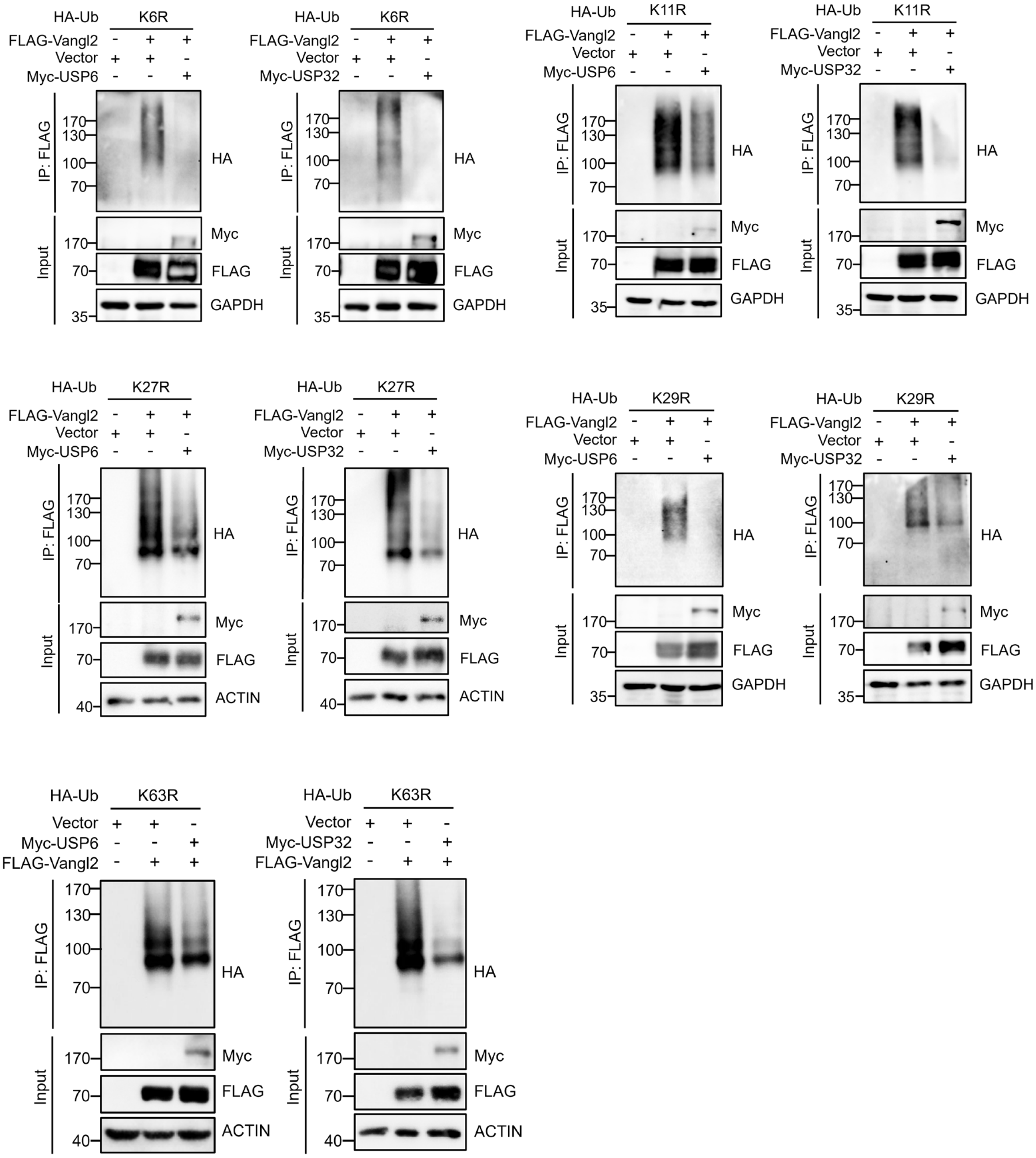

HA-Ub
K6R
FLAG-Vangl2
Vector
Myc-USP6
IP: FLAG
170
130
100
70
HA
Input
Myc
FLAG
GAPDH
35
Myc-USP32
K11R
K27R
ACTIN
40
K29R
K63R

Figure S3

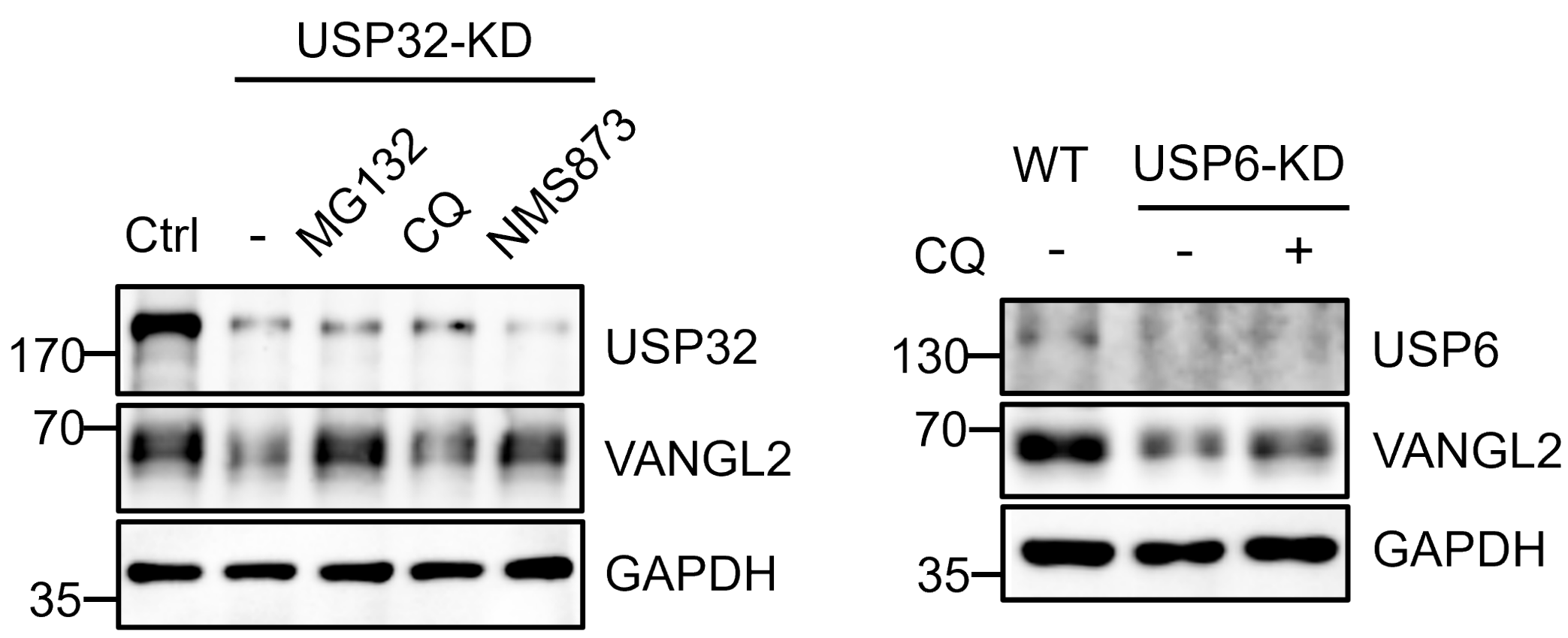

USP32-KD
Ctrl
-
MG132
CQ
NMS873
170
70
35
USP32
VANGL2
GAPDH
WT
USP6-KD
CQ
-
-
+
130
70
35
USP6
VANGL2
GAPDH

Figure S4

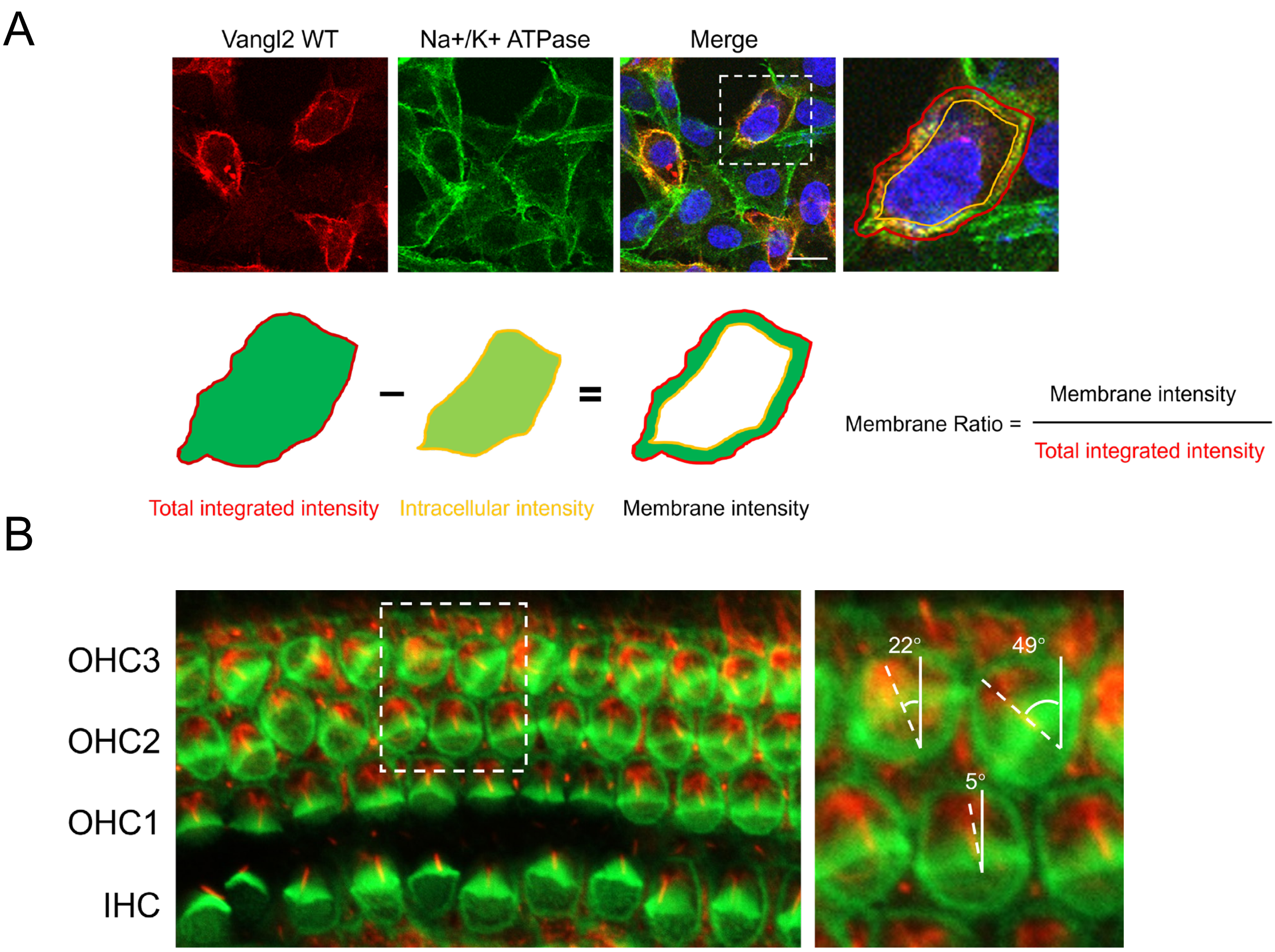
A
Vangl2 WT
Na+/K+ ATPase
Merge
−
=
Membrane Ratio = Membrane intensity / Total integrated intensity
Total integrated intensity
Intracellular intensity
Membrane intensity
B
OHC3
OHC2
OHC1
IHC
22°
49°
5°

Figure S5

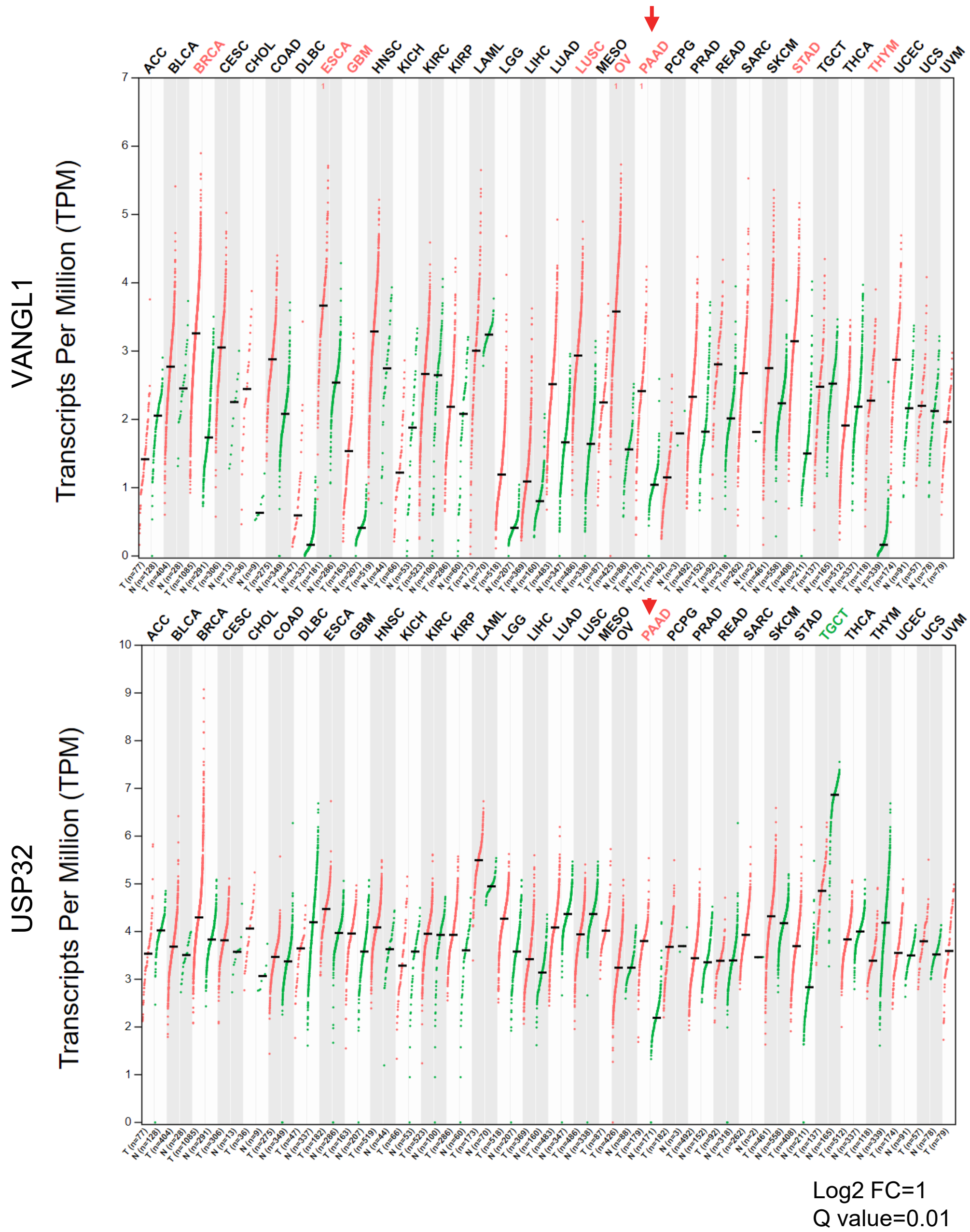
VANGL1
Transcripts Per Million (TPM)
ACC BLCA BRCA CESC CHOL COAD DLBC ESCA GBM HNSC KICH KIRC KIRP LAML LGG LIHC LUAD LUSC MESO OV PAAD PCPG PRAD READ SARC SKCM STAD TGCT THCA THYM UCEC UCS UVM
USP32
Transcripts Per Million (TPM)
ACC BLCA BRCA CESC CHOL COAD DLBC ESCA GBM HNSC KICH KIRC KIRP LAML LGG LIHC LUAD LUSC MESO OV PAAD PCPG PRAD READ SARC SKCM STAD TGCT THCA THYM UCEC UCS UVM
Log2 FC=1
Q value=0.01

Figure S6

A

Model	Method	n	HR	95% CI	P Value
USP32 High Expression	Univariate COX	177	1.20	0.80-1.81	0.385
VANGL1 High Expression	Univariate COX	177	1.89	1.23-2.89	0.004
Dual High Expression	Univariate COX	177	1.48	0.97-2.25	0.068
USP32 High Expression	Multivariate COX	174	1.25	0.82-1.90	0.310
VANGL1 High Expression	Multivariate COX	174	1.66	1.07-2.58	0.024
Dual High Expression	Multivariate COX	174	1.34	0.87-2.07	0.182

B

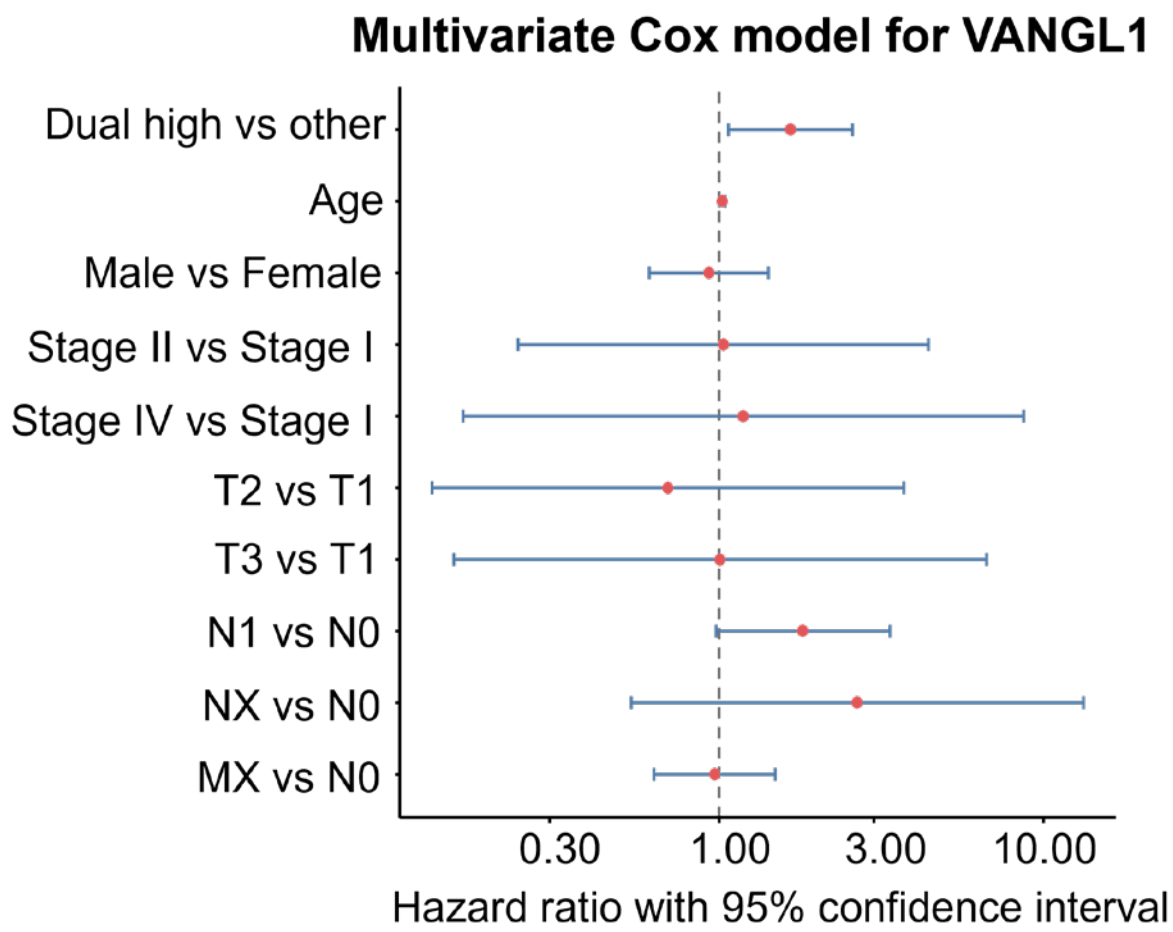

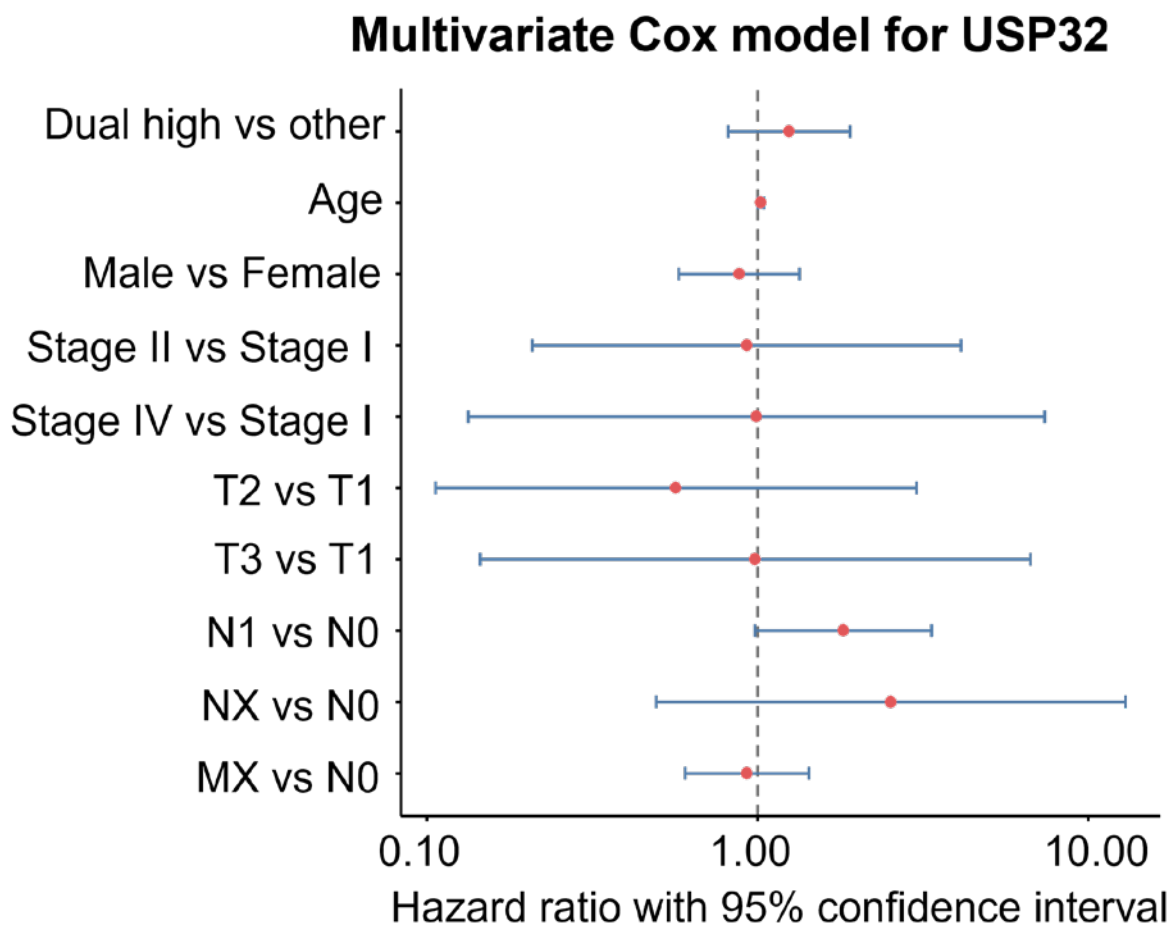

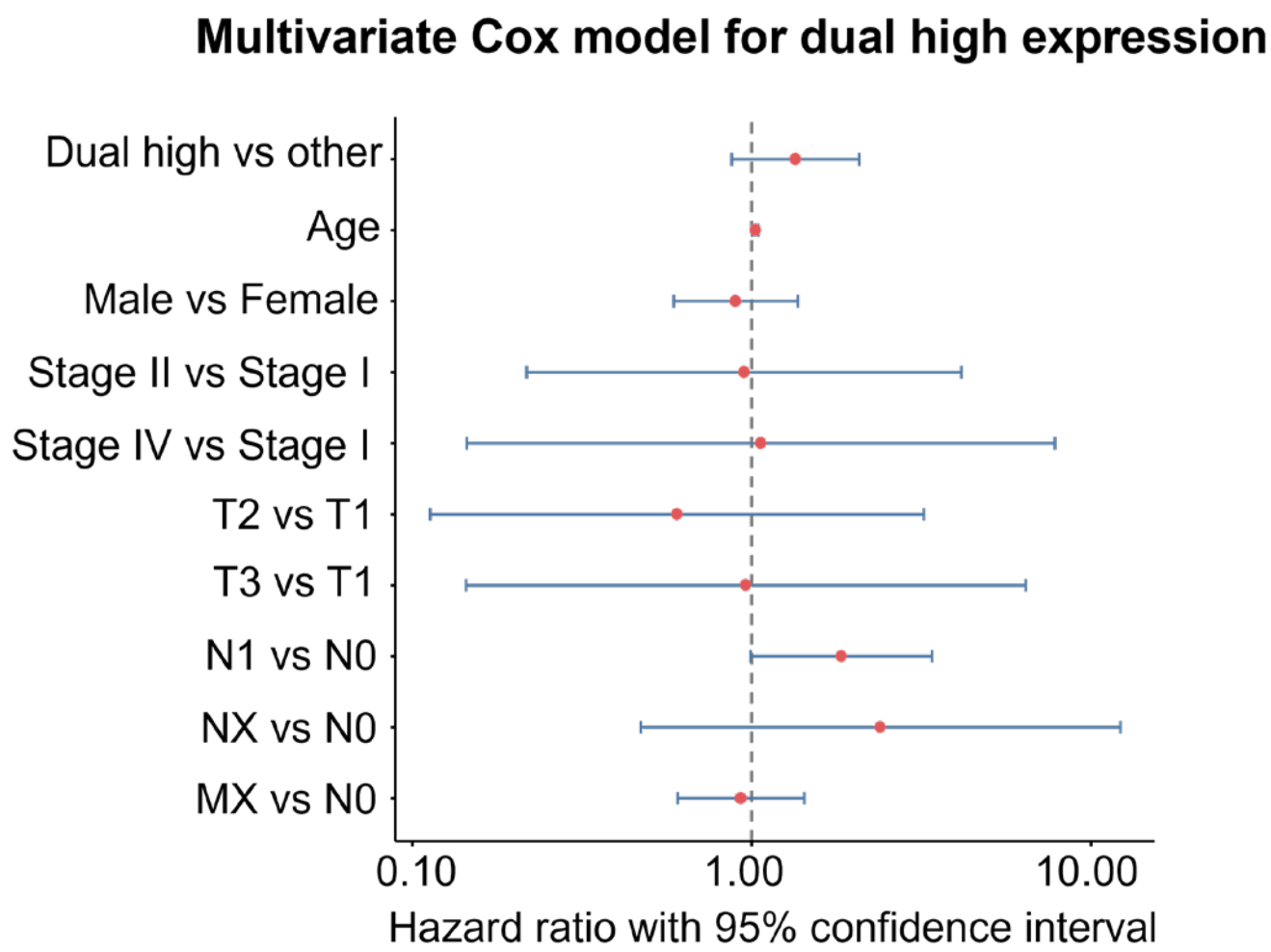

Figure S7

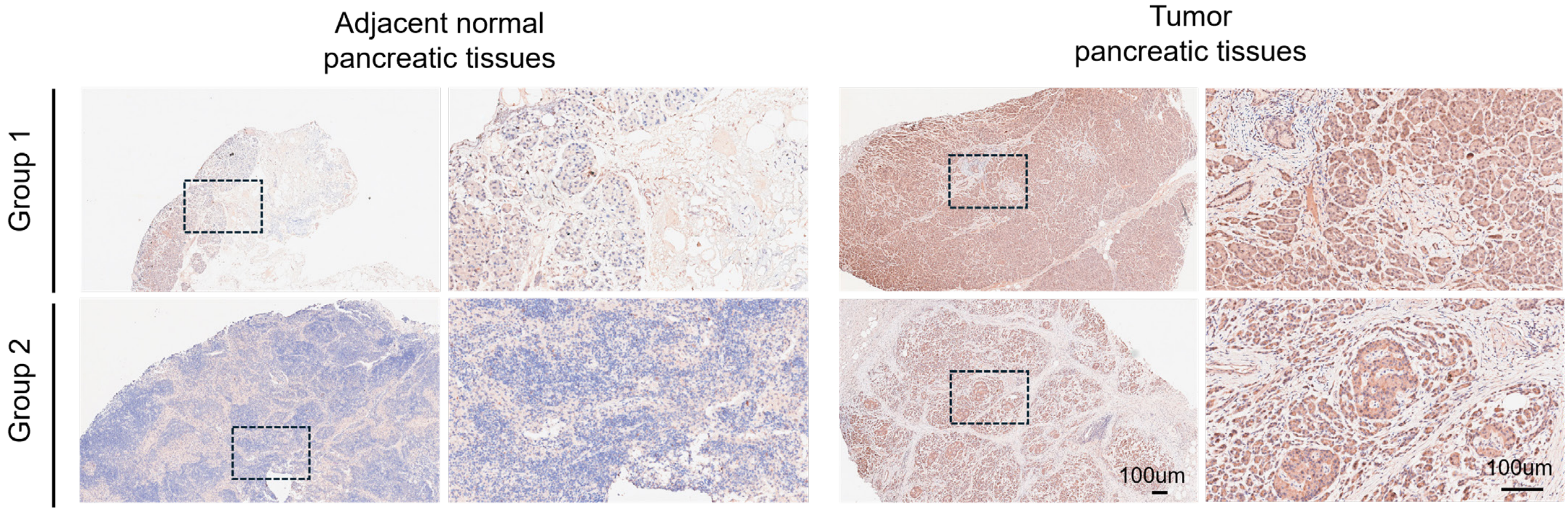

Adjacent normal pancreatic tissues
Tumor pancreatic tissues
Group 1
Group 2
100um
100um

Figure S8

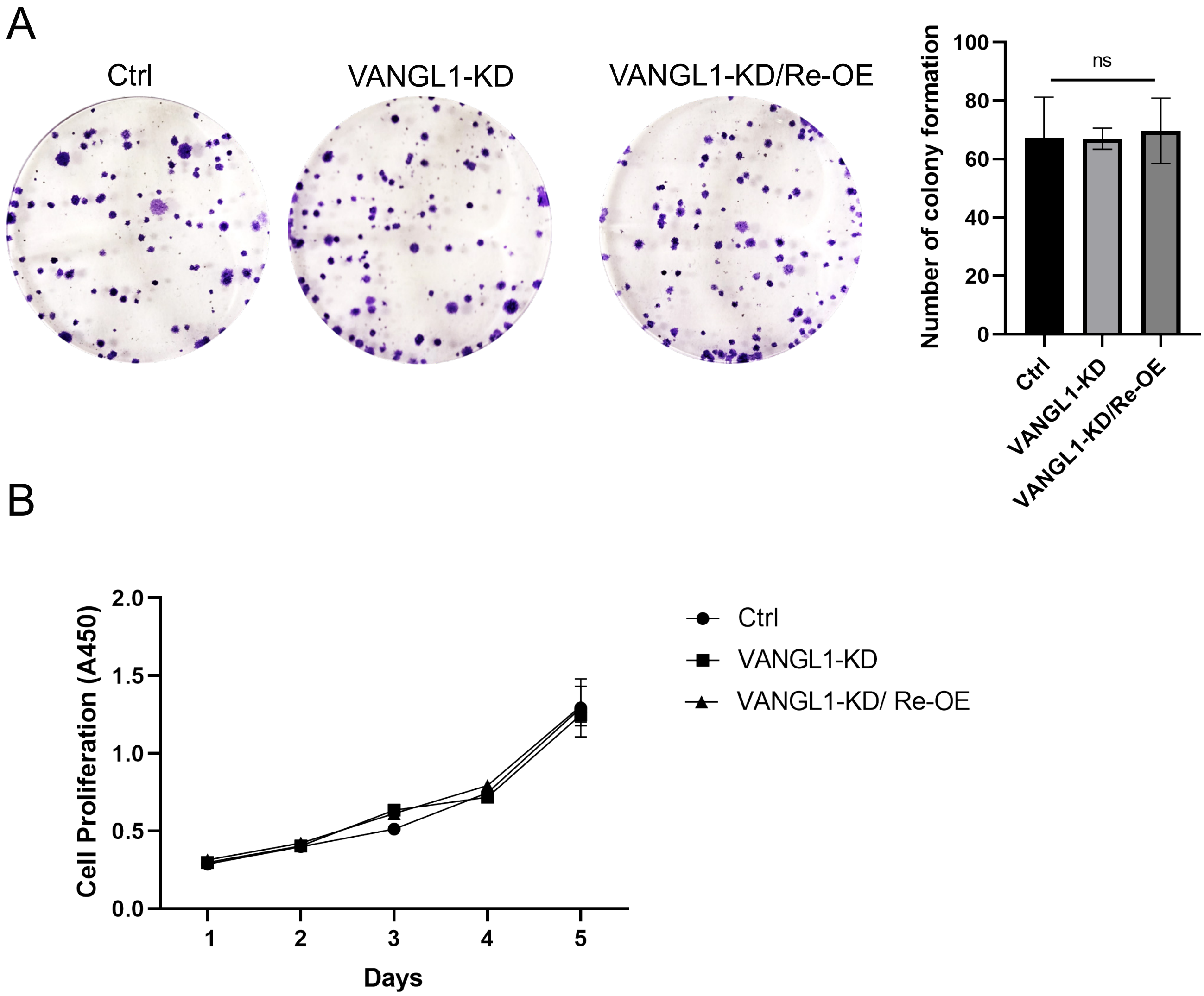

A
Ctrl
VANGL1-KD
VANGL1-KD/Re-OE
Number of colony formation
100
80
60
40
20
0
ns
Ctrl
VANGL1-KD
VANGL1-KD/Re-OE
B
Cell Proliferation (A450)
2.0
1.5
1.0
0.5
0.0
1
2
3
4
5
Days
Ctrl
VANGL1-KD
VANGL1-KD/ Re-OE

Figure S9

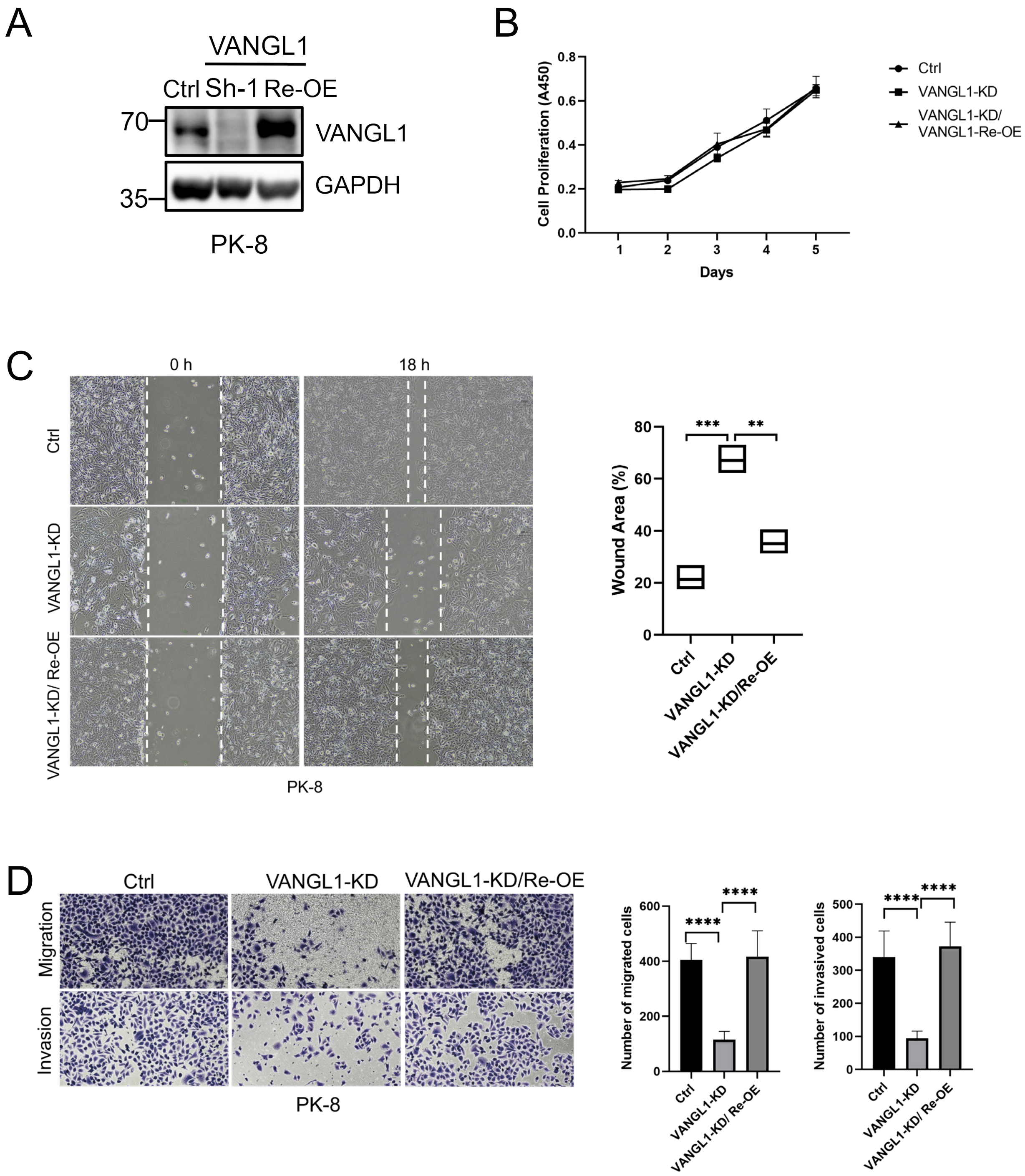
A
VANGL1
Ctrl Sh-1 Re-OE
70
35
VANGL1
GAPDH
PK-8
B
Cell Proliferation (A450)
0.8
0.6
0.4
0.2
0.0
1 2 3 4 5
Days
Ctrl
VANGL1-KD
VANGL1-KD/
VANGL1-Re-OE
C
0 h
18 h
Ctrl
VANGL1-KD
VANGL1-KD/ Re-OE
PK-8
Wound Area (%)
80
60
40
20
0
***
**
Ctrl
VANGL1-KD
VANGL1-KD/Re-OE
D
Ctrl
VANGL1-KD
VANGL1-KD/Re-OE
Migration
Invasion
PK-8
Number of migrated cells
600
400
200
0
****
****
Ctrl
VANGL1-KD
VANGL1-KD/ Re-OE
Number of invasived cells
500
400
300
200
100
0
****
****
Ctrl
VANGL1-KD
VANGL1-KD/ Re-OE

Figure S10

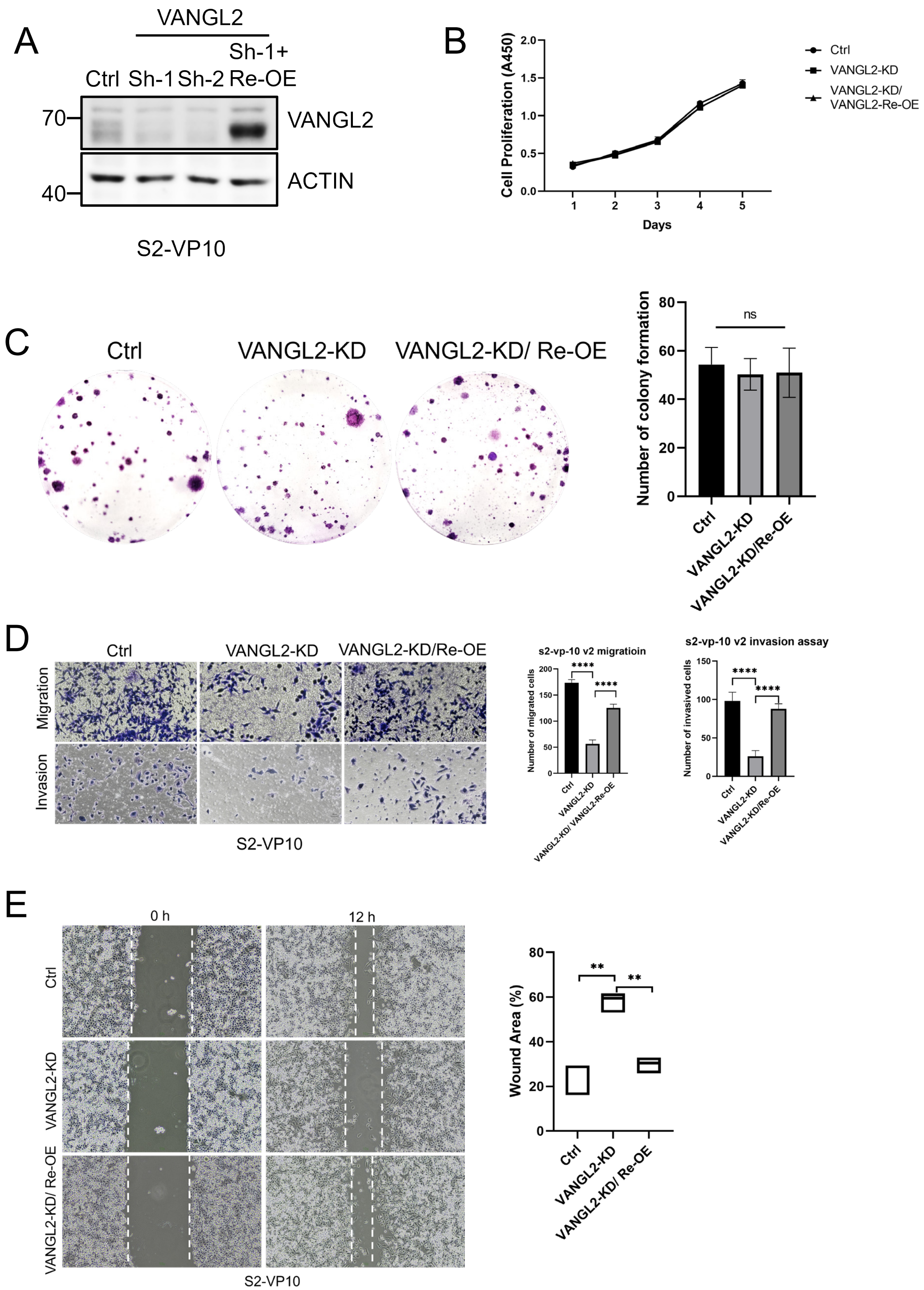

A
VANGL2
Ctrl
Sh-1
Sh-2
Sh-1+ Re-OE
70
40
VANGL2
ACTIN
S2-VP10
B
Cell Proliferation (A450)
Days
Ctrl
VANGL2-KD
VANGL2-KD/ VANGL2-Re-OE
C
Ctrl
VANGL2-KD
VANGL2-KD/ Re-OE
Number of colony formation
ns
VANGL2-KD/Re-OE
D
Migration
Invasion
VANGL2-KD/Re-OE
S2-VP10
s2-vp-10 v2 migratioin
Number of migrated cells
VANGL2-KD/ VANGL2-Re-OE
s2-vp-10 v2 invasion assay
Number of invasived cells
E
0 h
12 h
VANGL2-KD/ Re-OE
Wound Area (%)

Figure S11

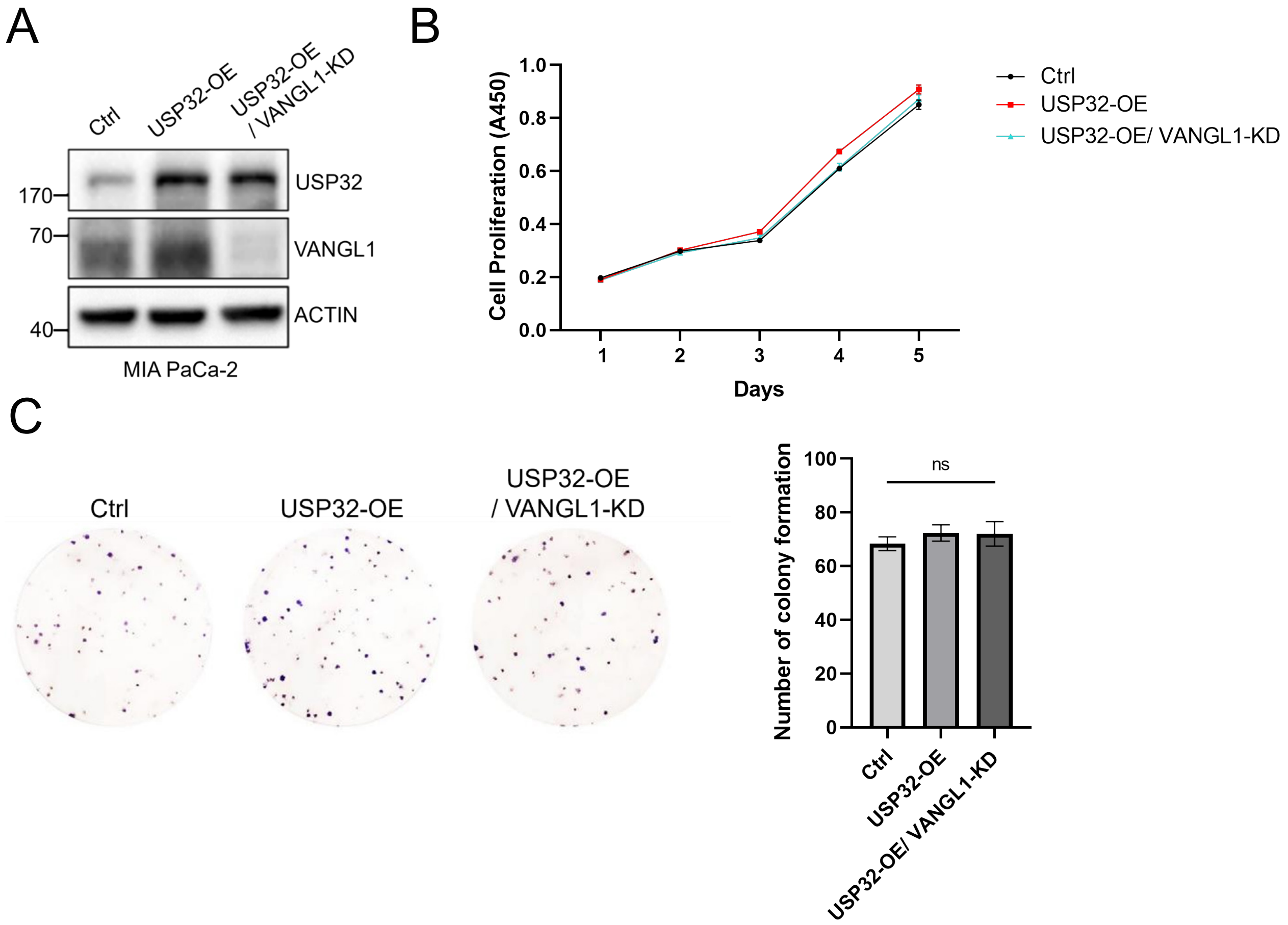
A
Ctrl
USP32-OE
USP32-OE / VANGL1-KD
USP32
VANGL1
ACTIN
170
70
40
MIA PaCa-2
B
Cell Proliferation (A450)
Days
Ctrl
USP32-OE
USP32-OE/ VANGL1-KD
C
Ctrl
USP32-OE
USP32-OE / VANGL1-KD
Number of colony formation
ns
USP32-OE/ VANGL1-KD

Figure S12

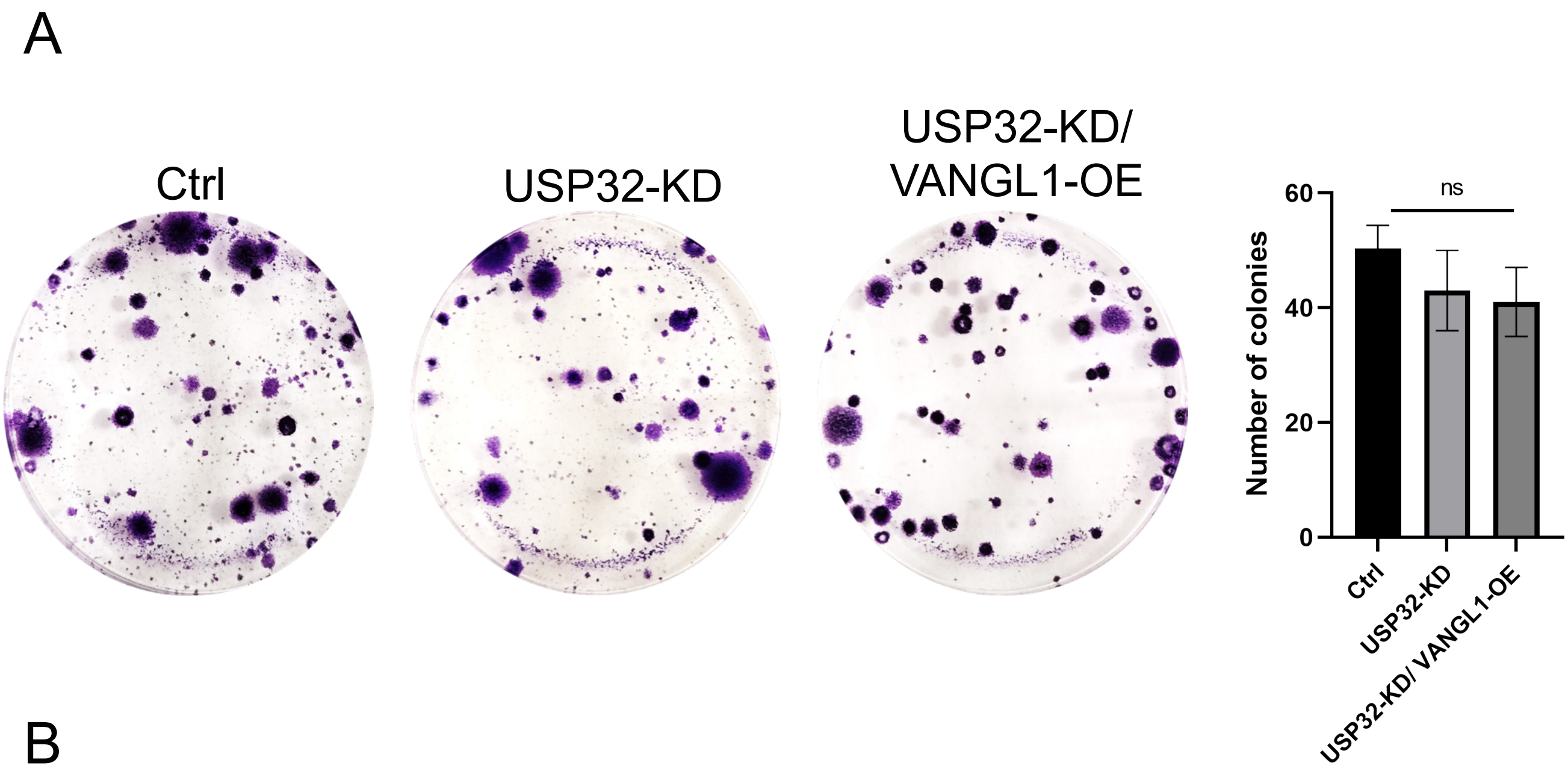

A
Ctrl
USP32-KD
USP32-KD/
VANGL1-OE
Number of colonies
60
40
20
0
ns
Ctrl
USP32-KD
USP32-KD/ VANGL1-OE

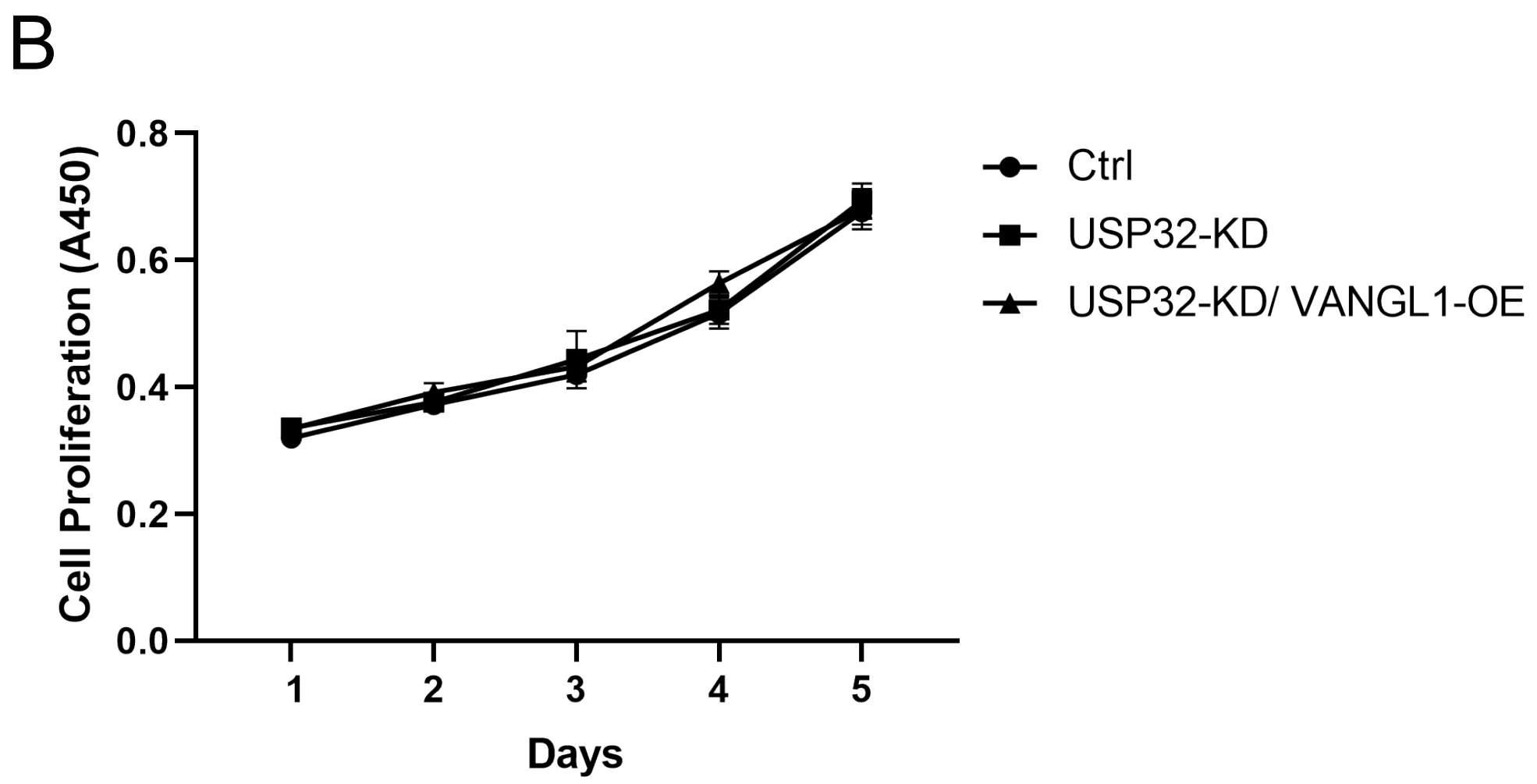

B
Cell Proliferation (A450)
0.8
0.6
0.4
0.2
0.0
1
2
3
4
5
Days
Ctrl
USP32-KD
USP32-KD/ VANGL1-OE

Figure S13

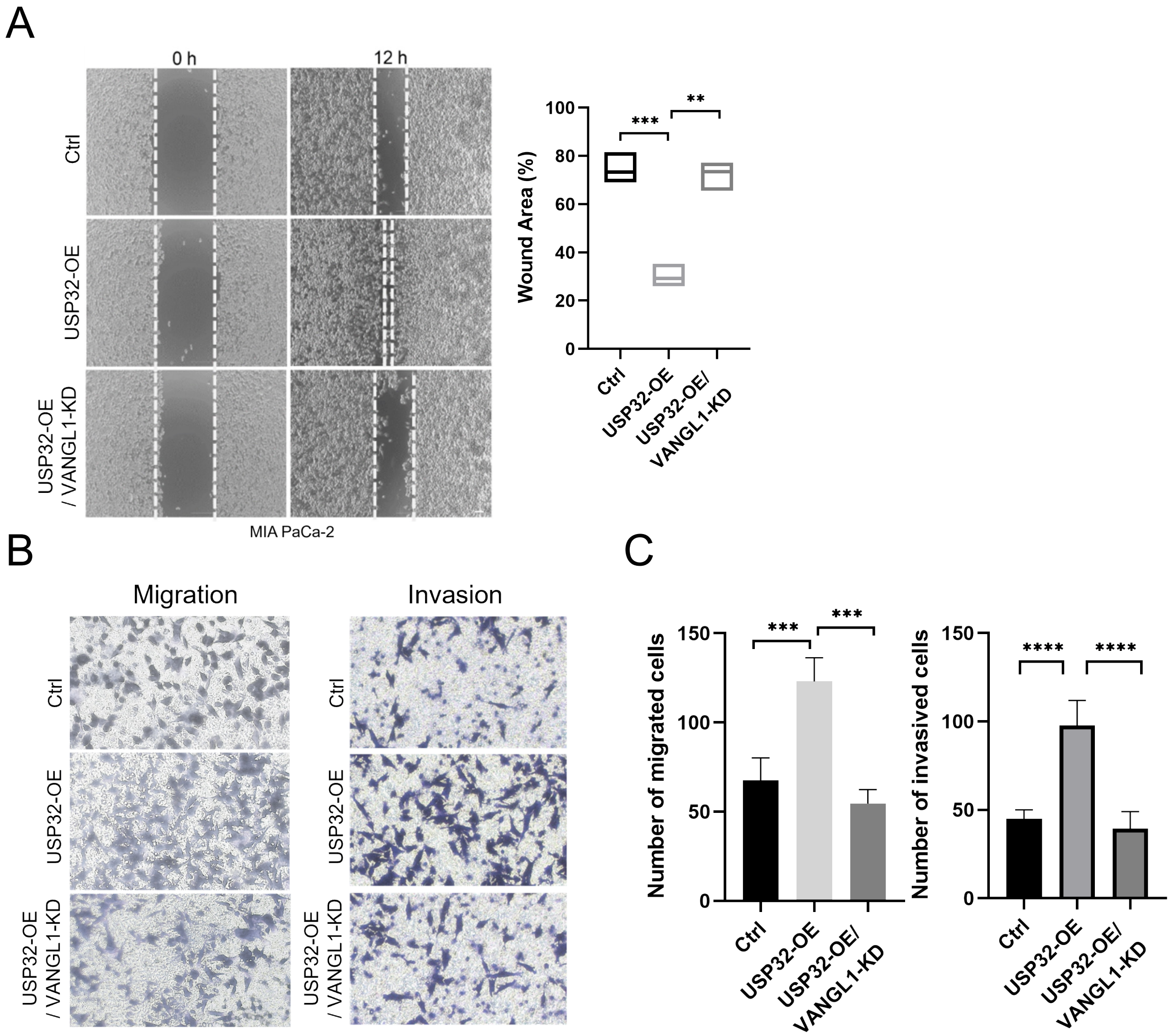
A
0 h
12 h
Ctrl
USP32-OE
USP32-OE / VANGL1-KD
MIA PaCa-2
Wound Area (%)
100
80
60
40
20
0
***
**
Ctrl
USP32-OE
USP32-OE/ VANGL1-KD
B
Migration
Invasion
Ctrl
USP32-OE
USP32-OE / VANGL1-KD
C
Number of migrated cells
150
100
50
0
***
***
Number of invasived cells
****
****
Ctrl
USP32-OE
USP32-OE/ VANGL1-KD

Figure S14

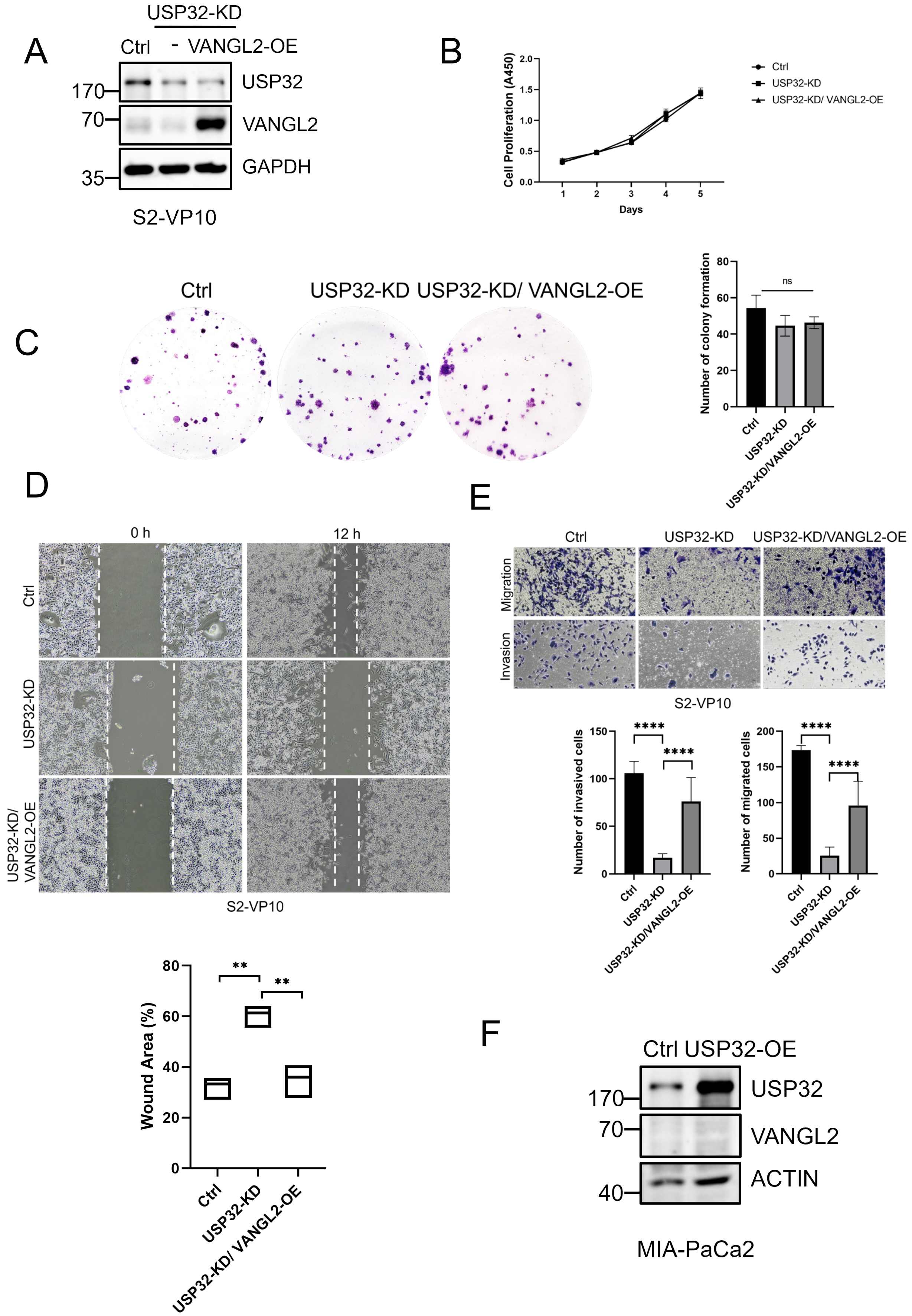

A
USP32-KD
Ctrl
-
VANGL2-OE
170
70
35
USP32
VANGL2
GAPDH
S2-VP10
B
Cell Proliferation (A450)
Days
Ctrl
USP32-KD
USP32-KD/ VANGL2-OE
C
Ctrl
USP32-KD
USP32-KD/ VANGL2-OE
Number of colony formation
ns
USP32-KD/VANGL2-OE
D
0 h
12 h
Ctrl
USP32-KD
USP32-KD/ VANGL2-OE
S2-VP10
Wound Area (%)
**
USP32-KD/ VANGL2-OE
E
Ctrl
USP32-KD
USP32-KD/VANGL2-OE
Migration
Invasion
S2-VP10
Number of invasived cells
Number of migrated cells
****
F
Ctrl
USP32-OE
170
70
40
USP32
VANGL2
ACTIN
MIA-PaCa2

Figure S15

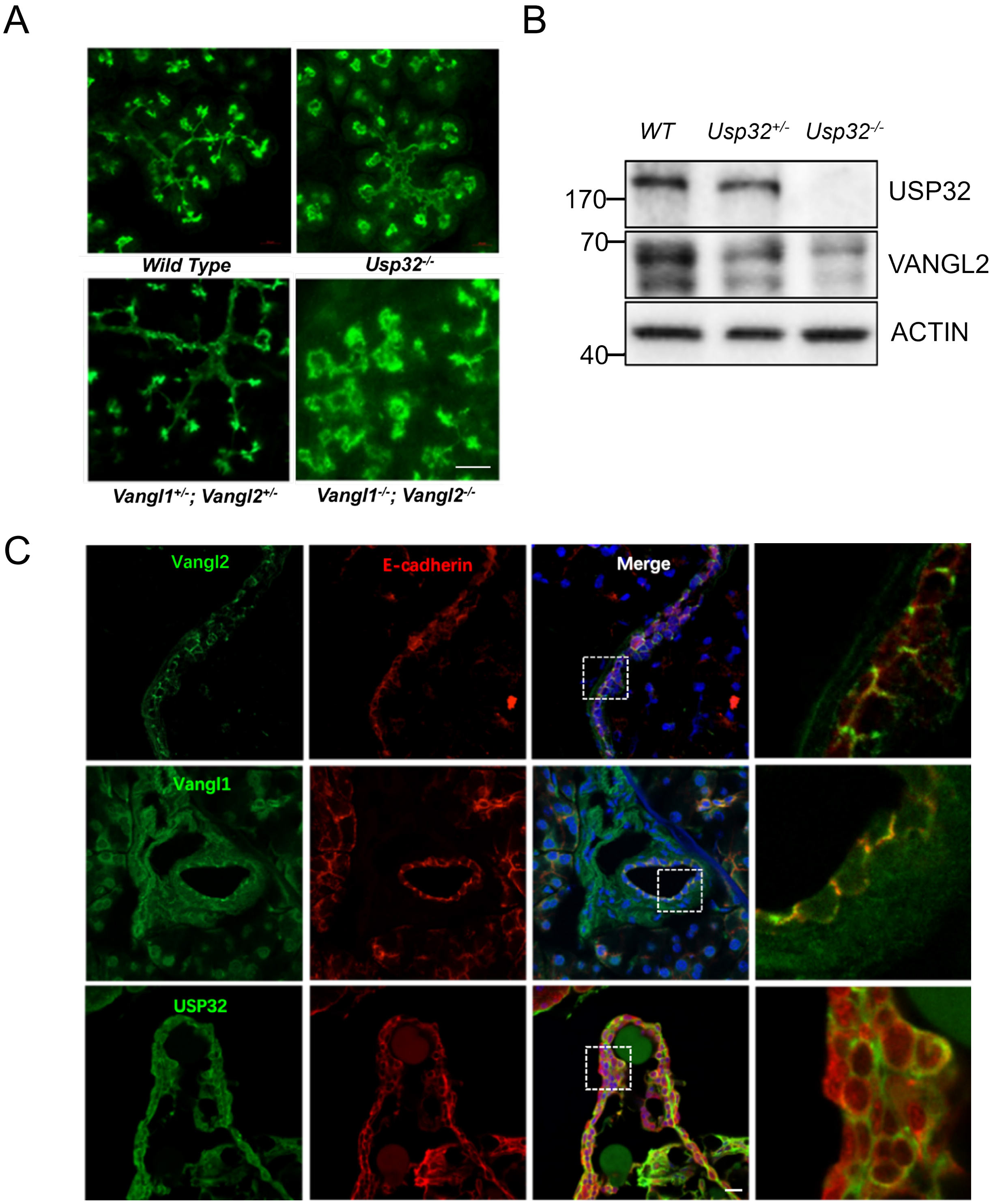
A
Wild Type
Usp32-/-
Vangl1+/-; Vangl2+/-
Vangl1-/-; Vangl2-/-
B
WT
Usp32+/-
Usp32-/-
170
70
40
USP32
VANGL2
ACTIN
C
Vangl2
E-cadherin
Merge
Vangl1
USP32

Figure S16

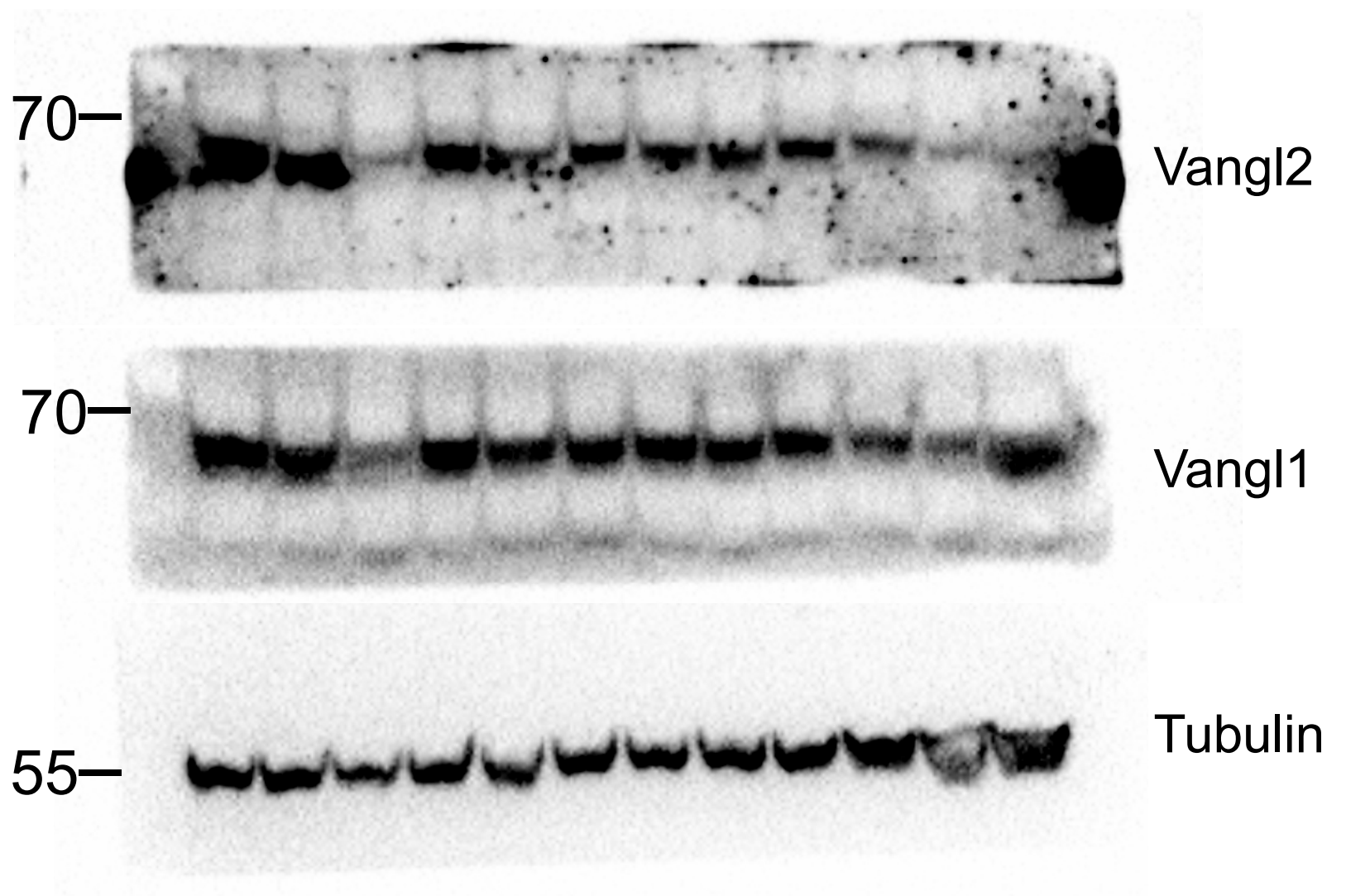

Figure 1C

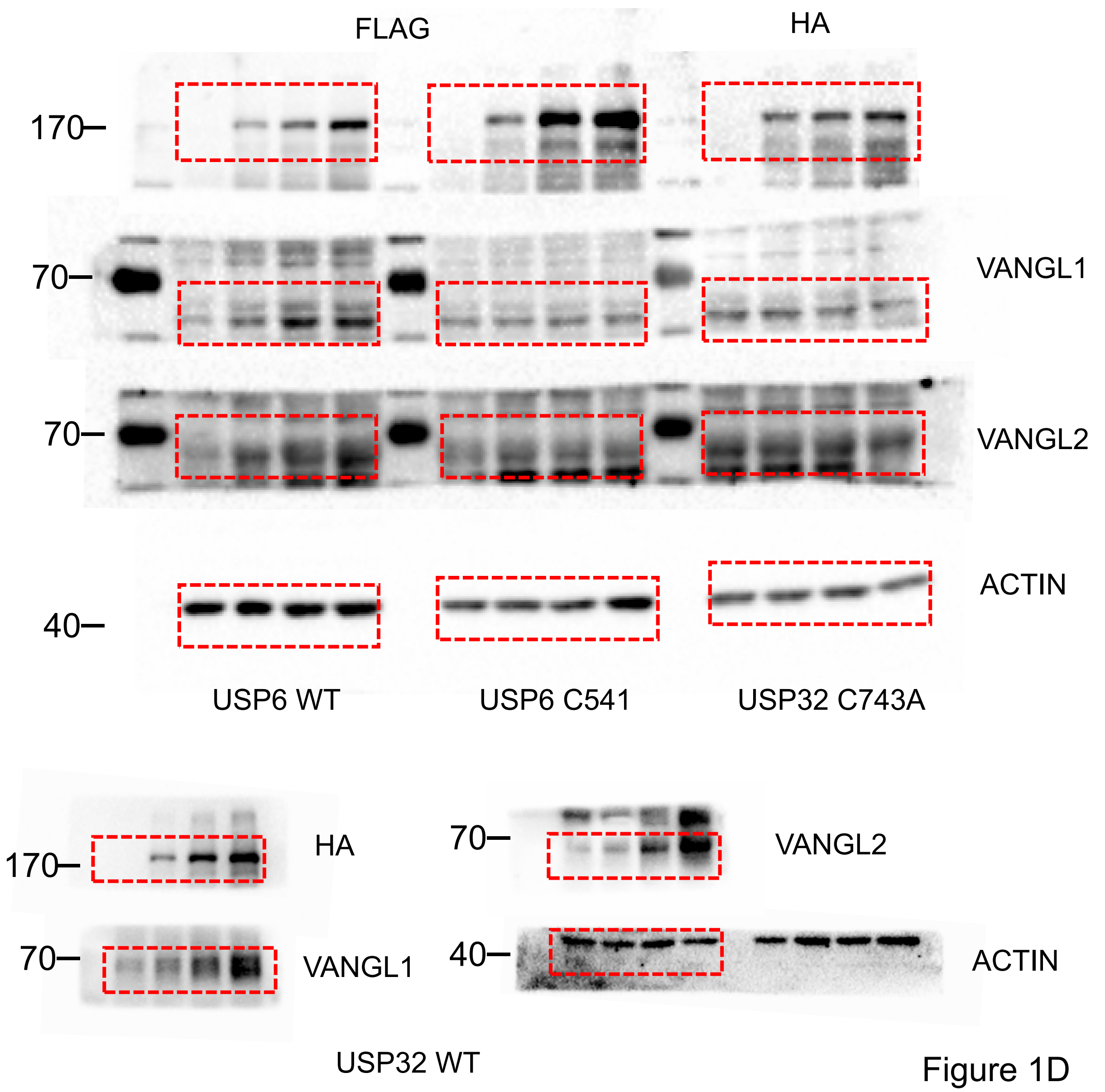

Figure 1D

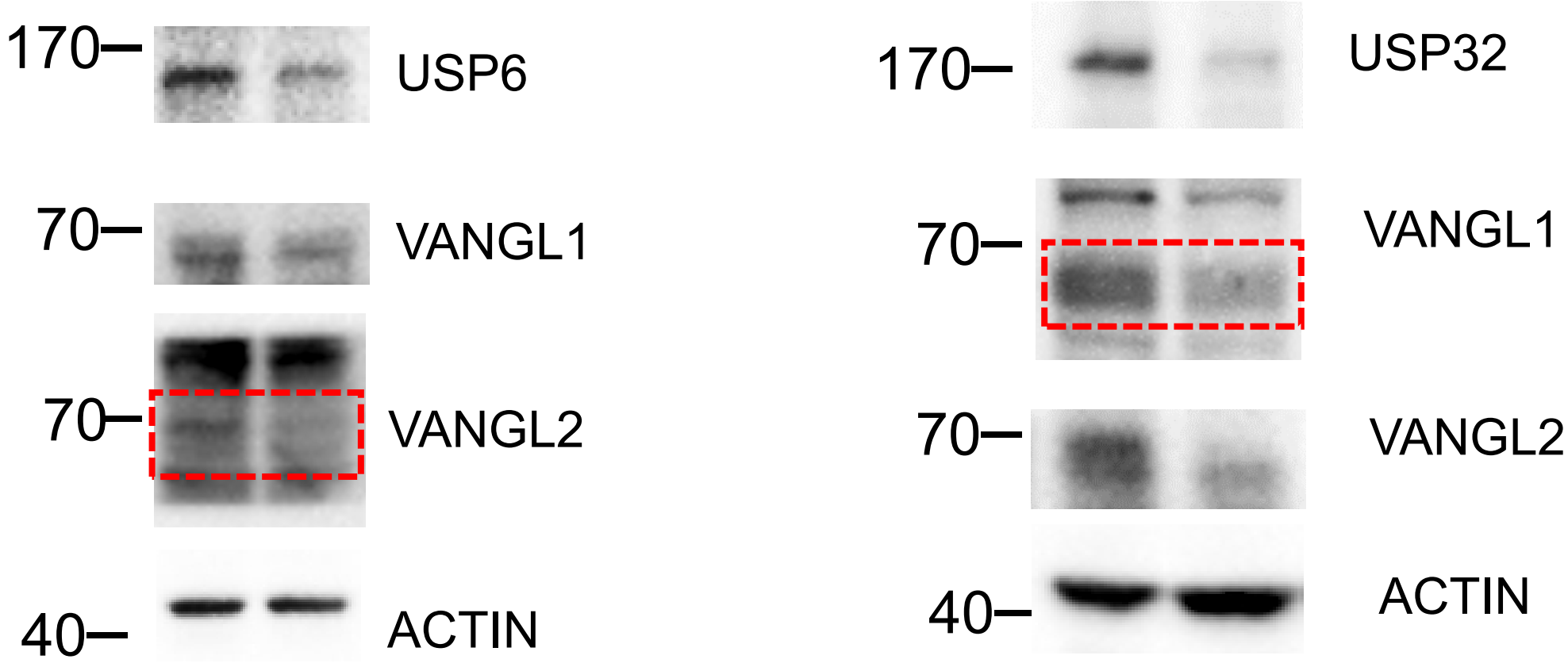

Figure 1E

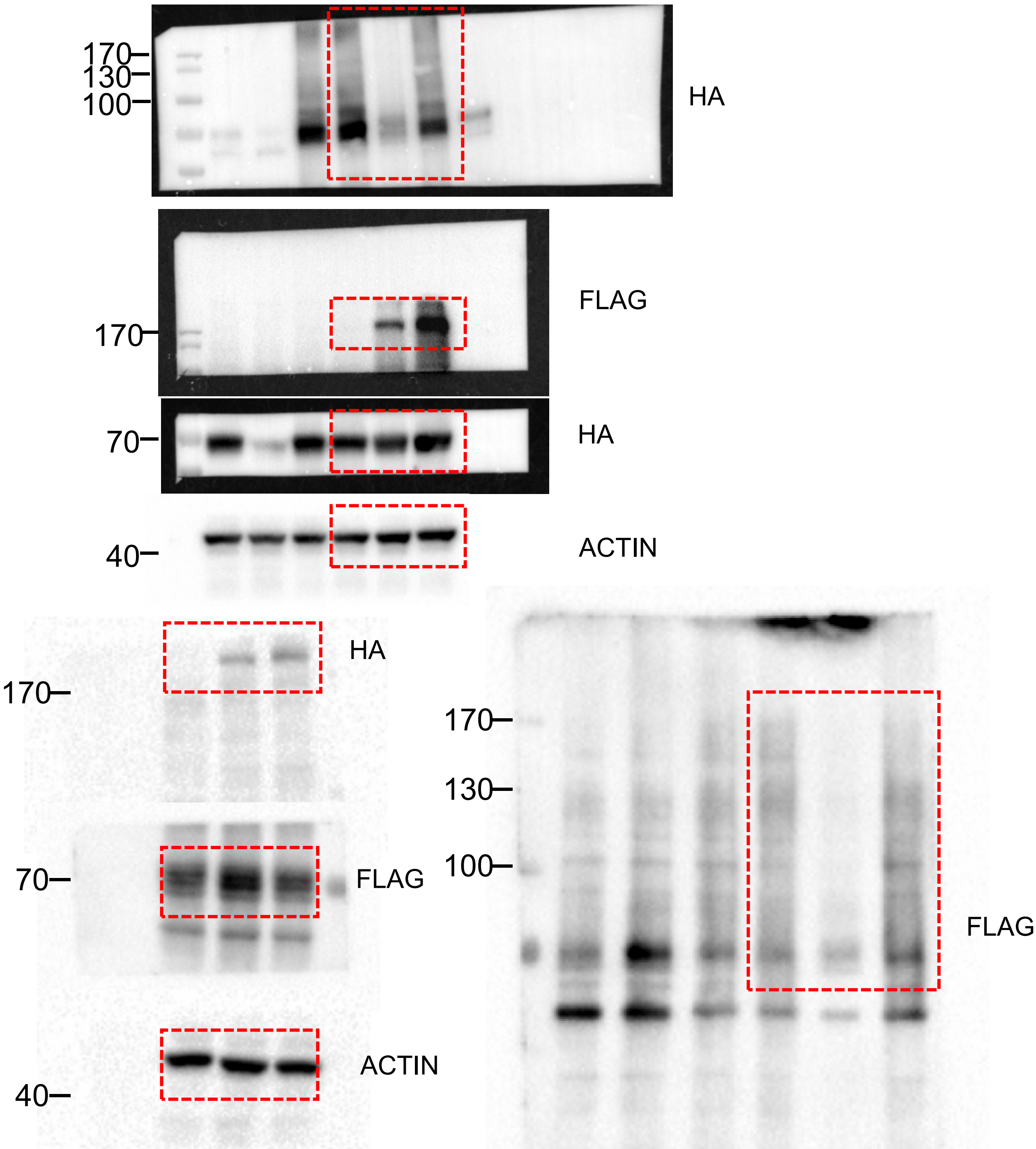

Figure 1F

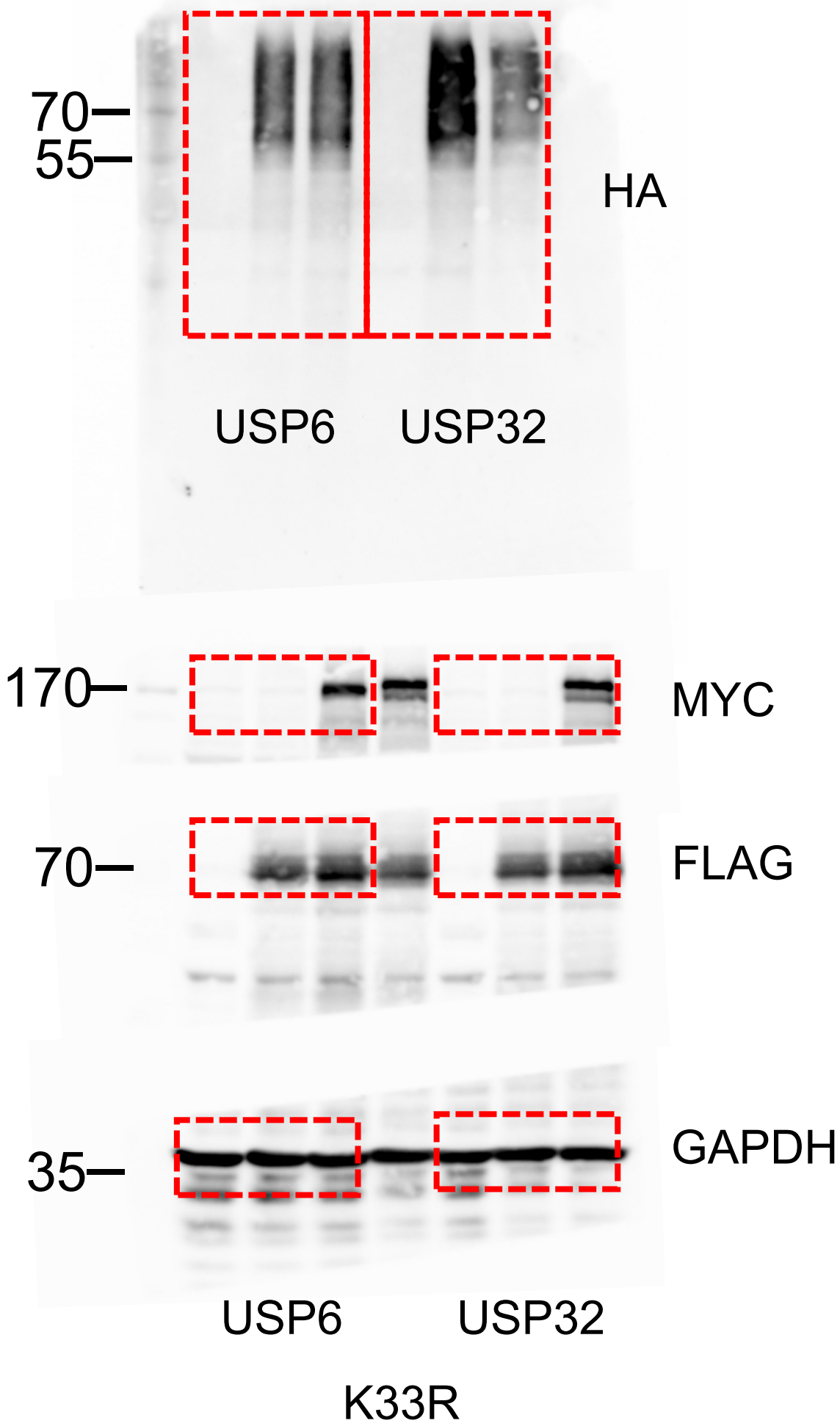

Figure 1G

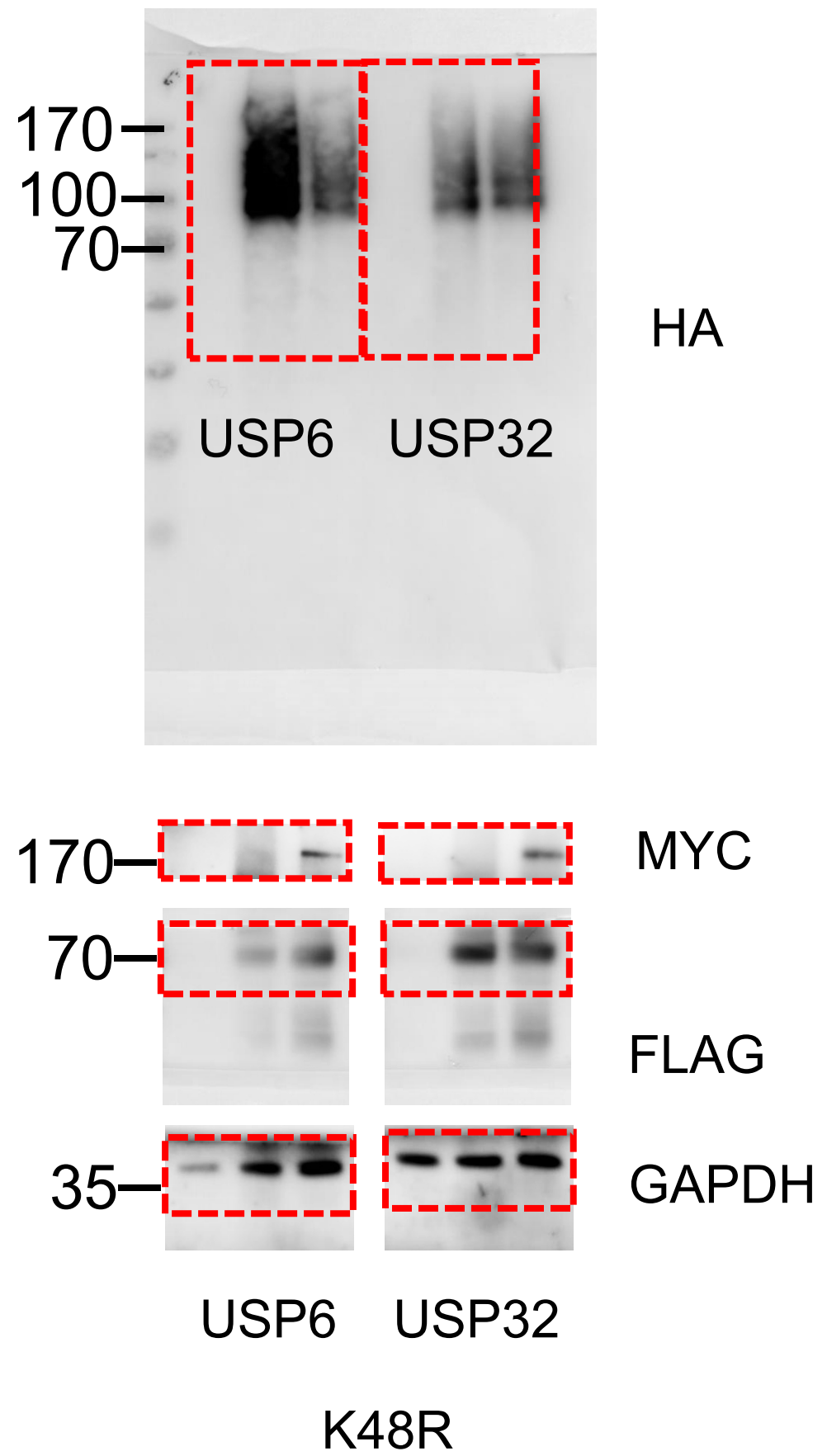

Figure 1H

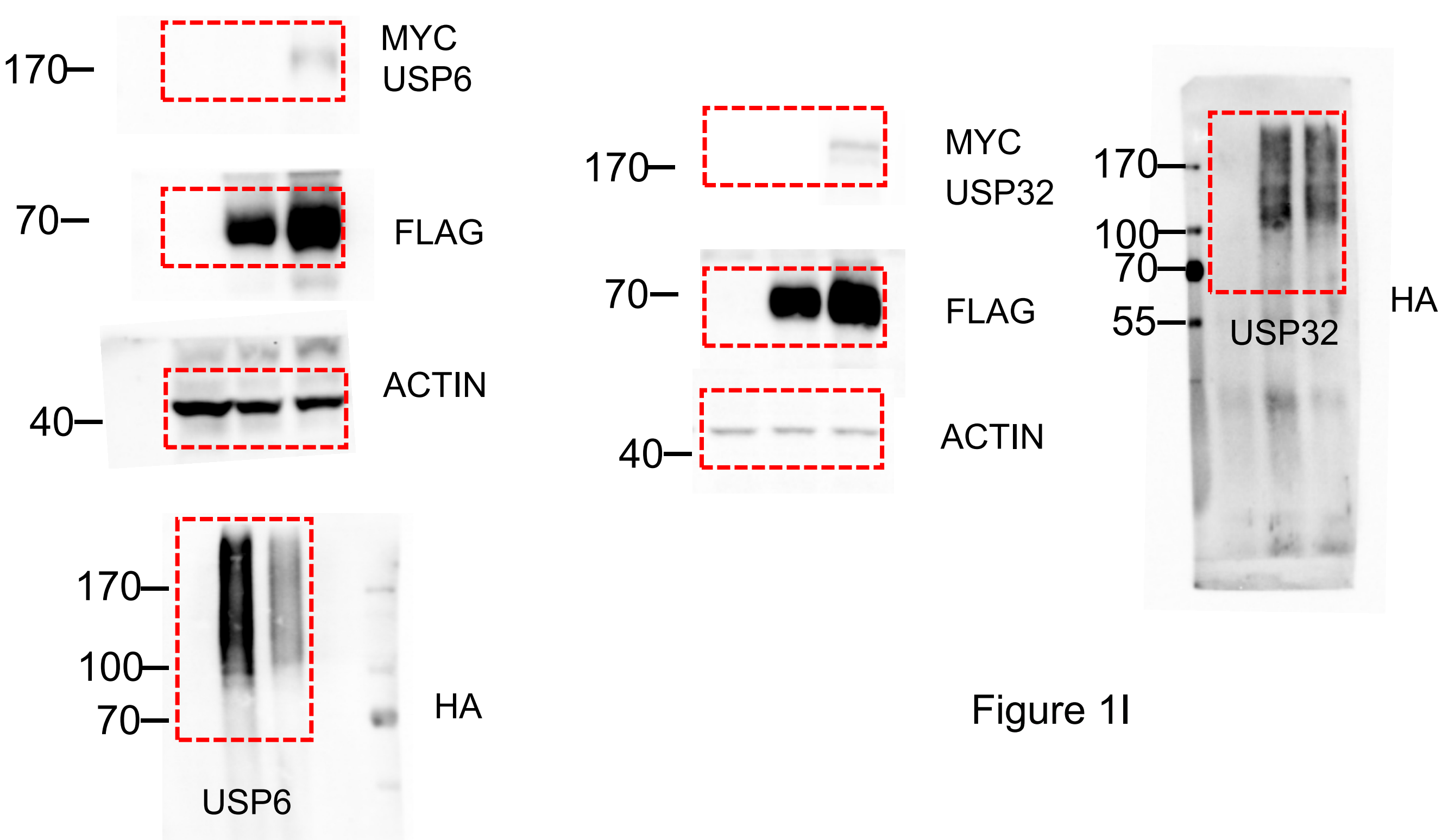

Figure 1I

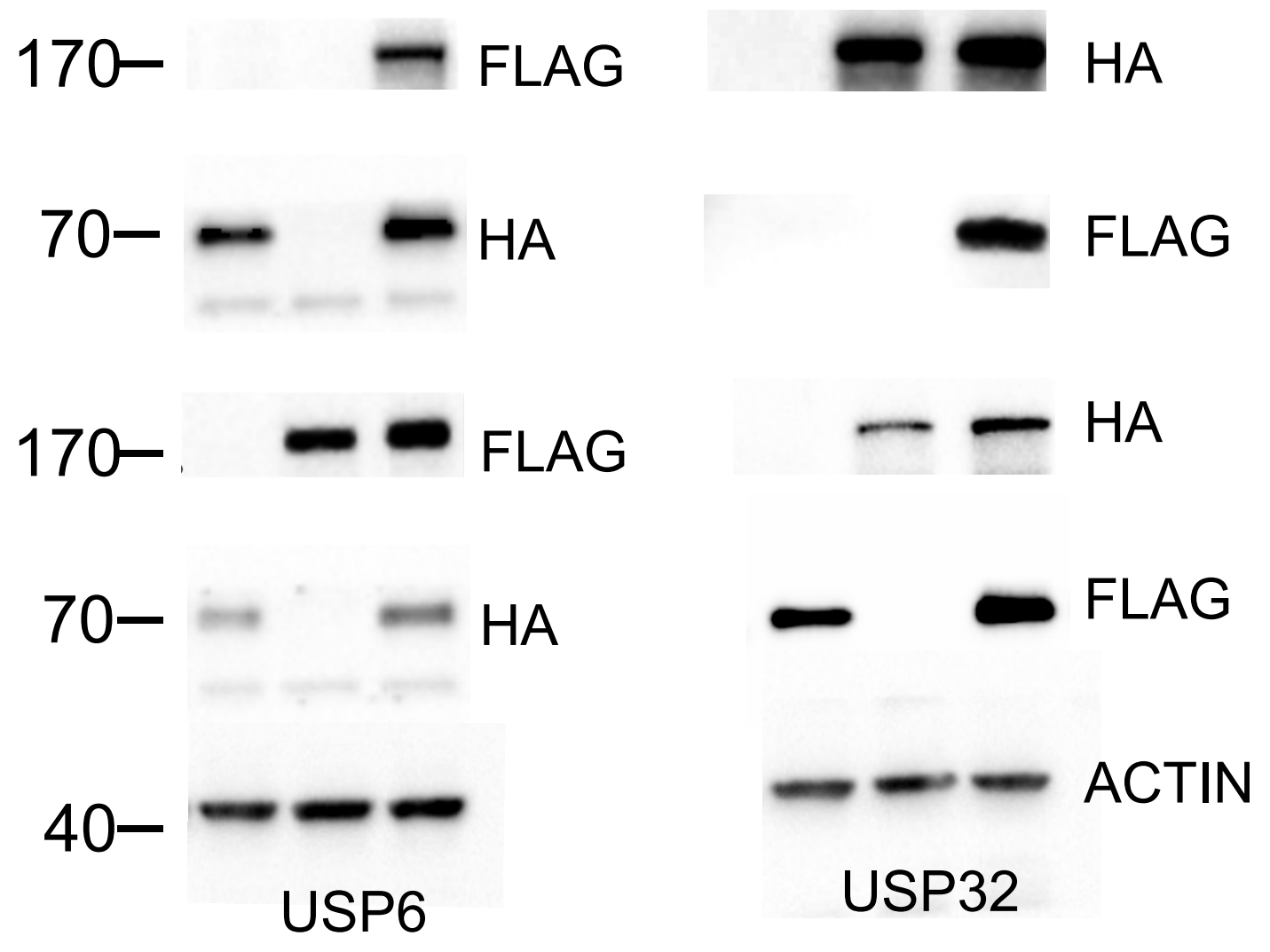

Figure 2A

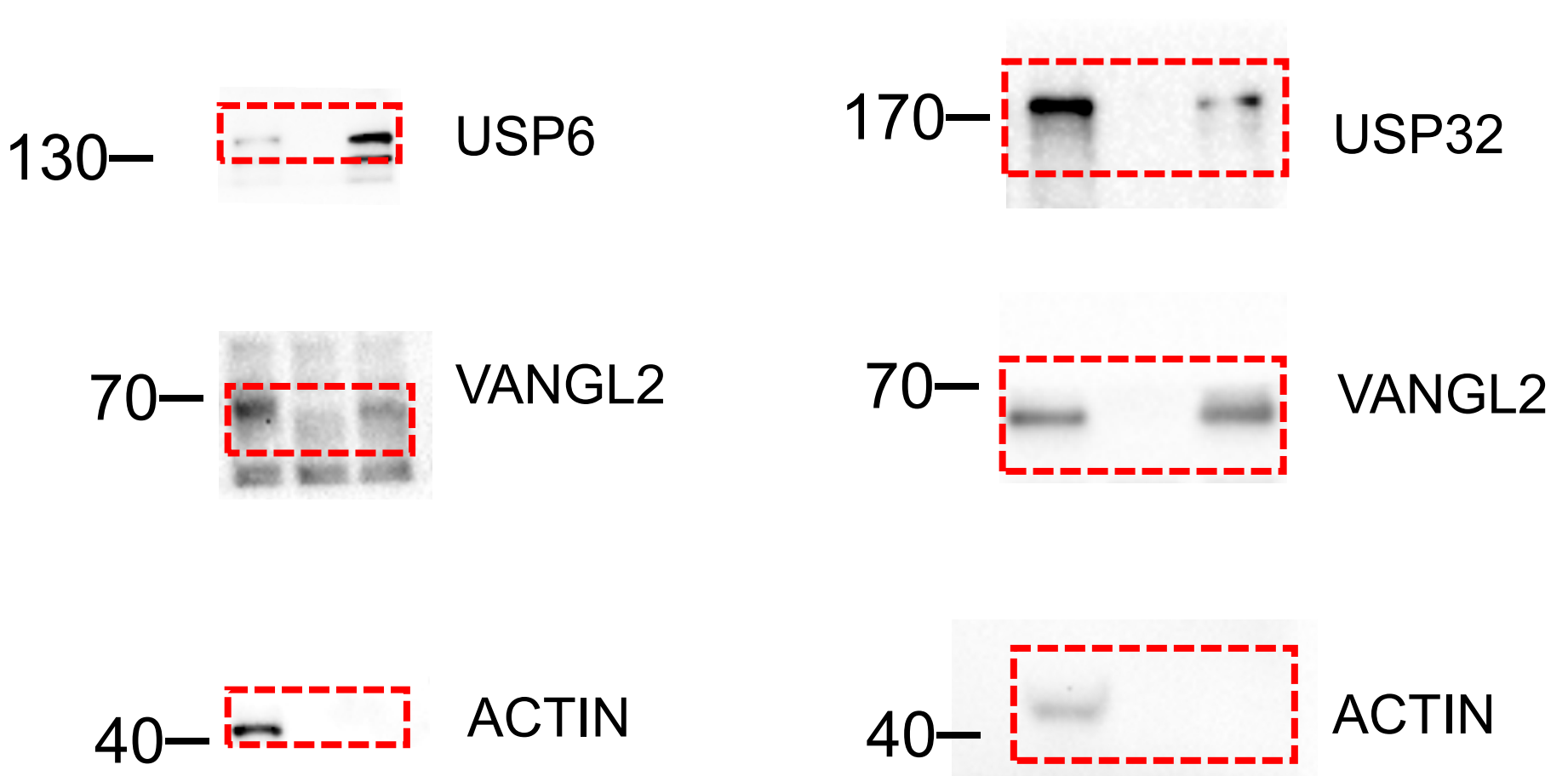

Figure 2B

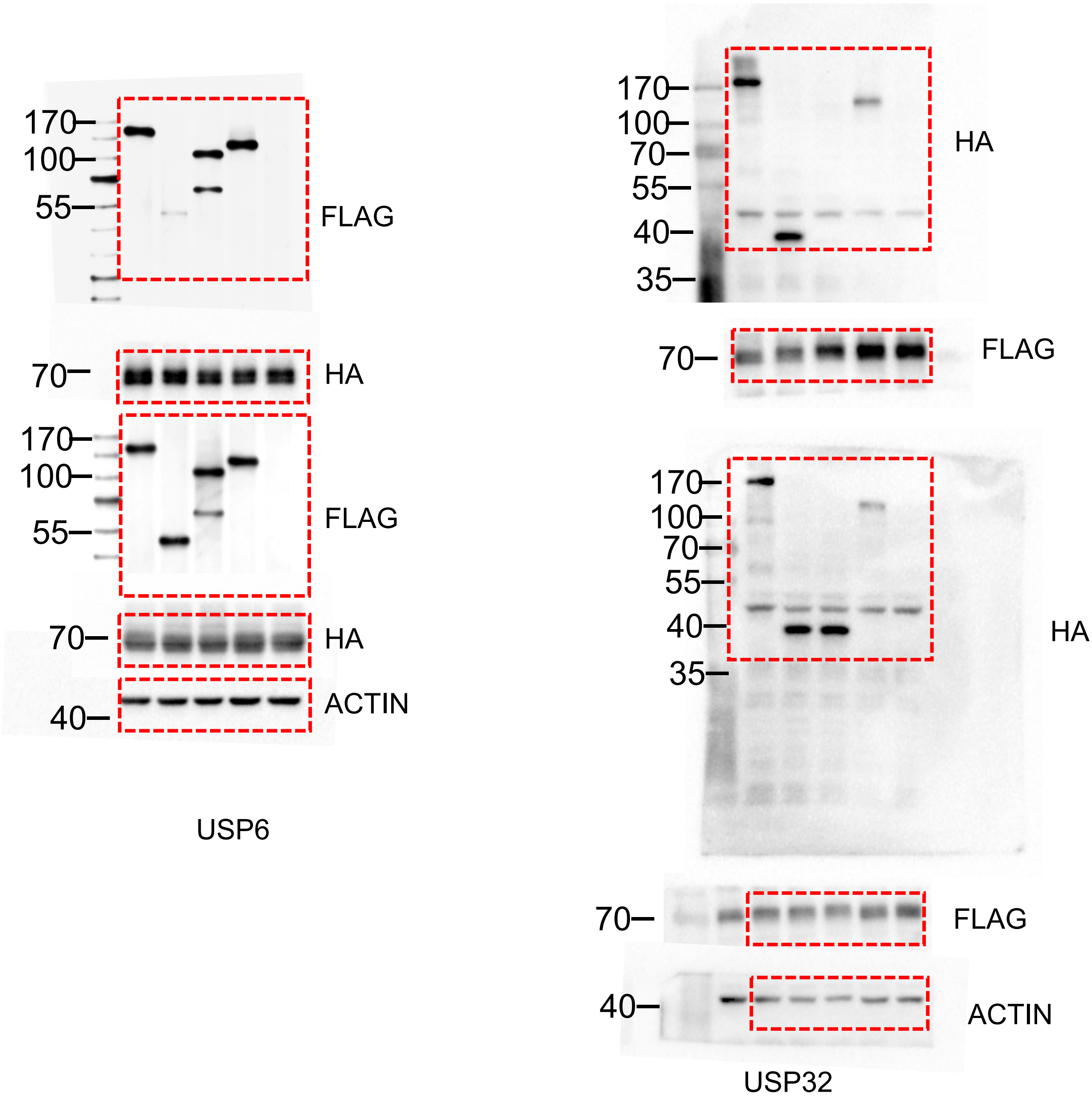
170
100
55
FLAG
70
HA
170
100
55
FLAG
70
HA
40
ACTIN
USP6
170
100
70
55
40
35
HA
70
FLAG
170
100
70
55
40
35
HA
70
FLAG
40
ACTIN
USP32

Figure 2D

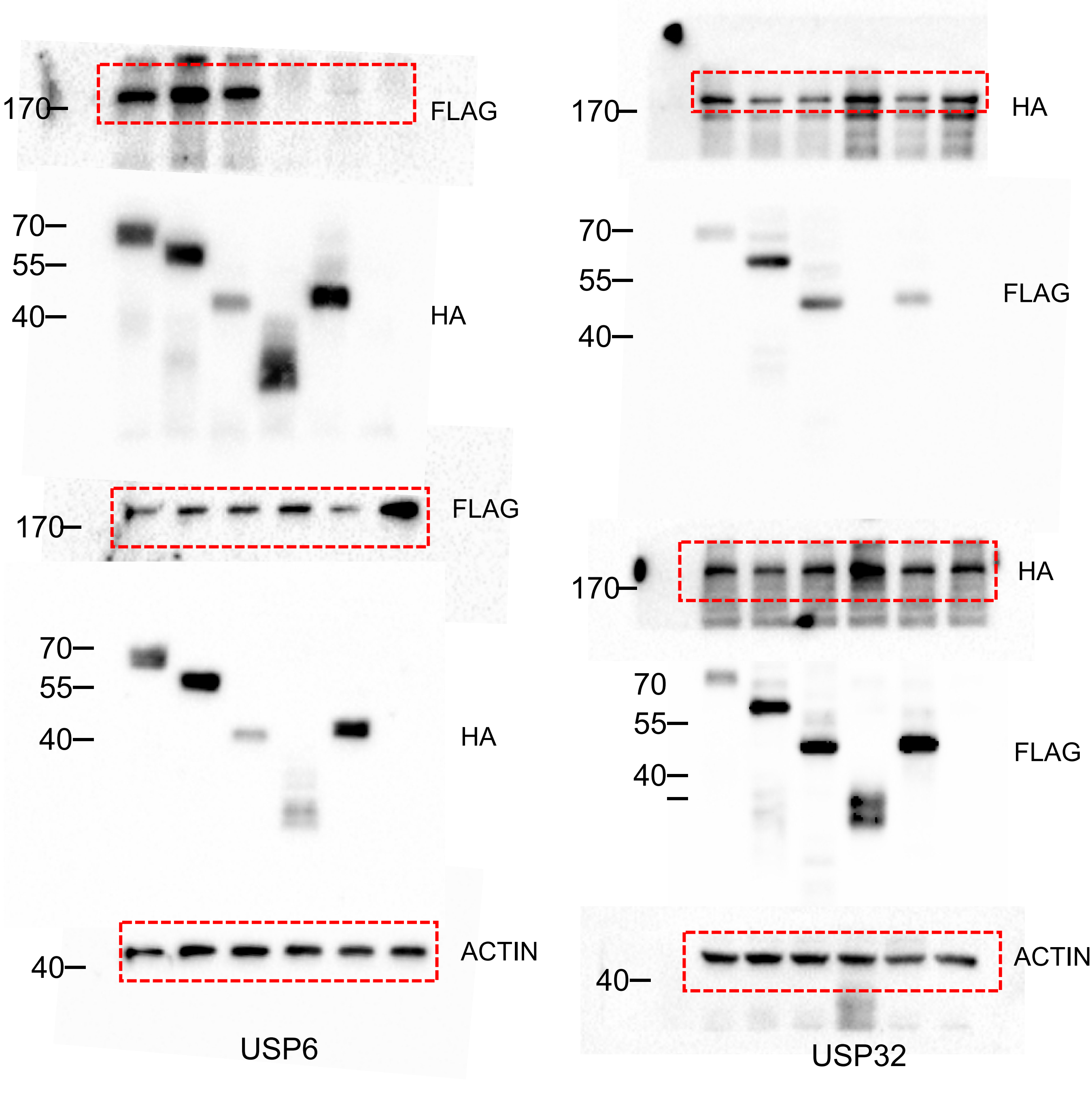
170
FLAG
70
55
40
HA
170
FLAG
70
55
40
HA
40
ACTIN
USP6
170
HA
70
55
40
FLAG
170
HA
70
55
40
FLAG
40
ACTIN
USP32

Figure 2F

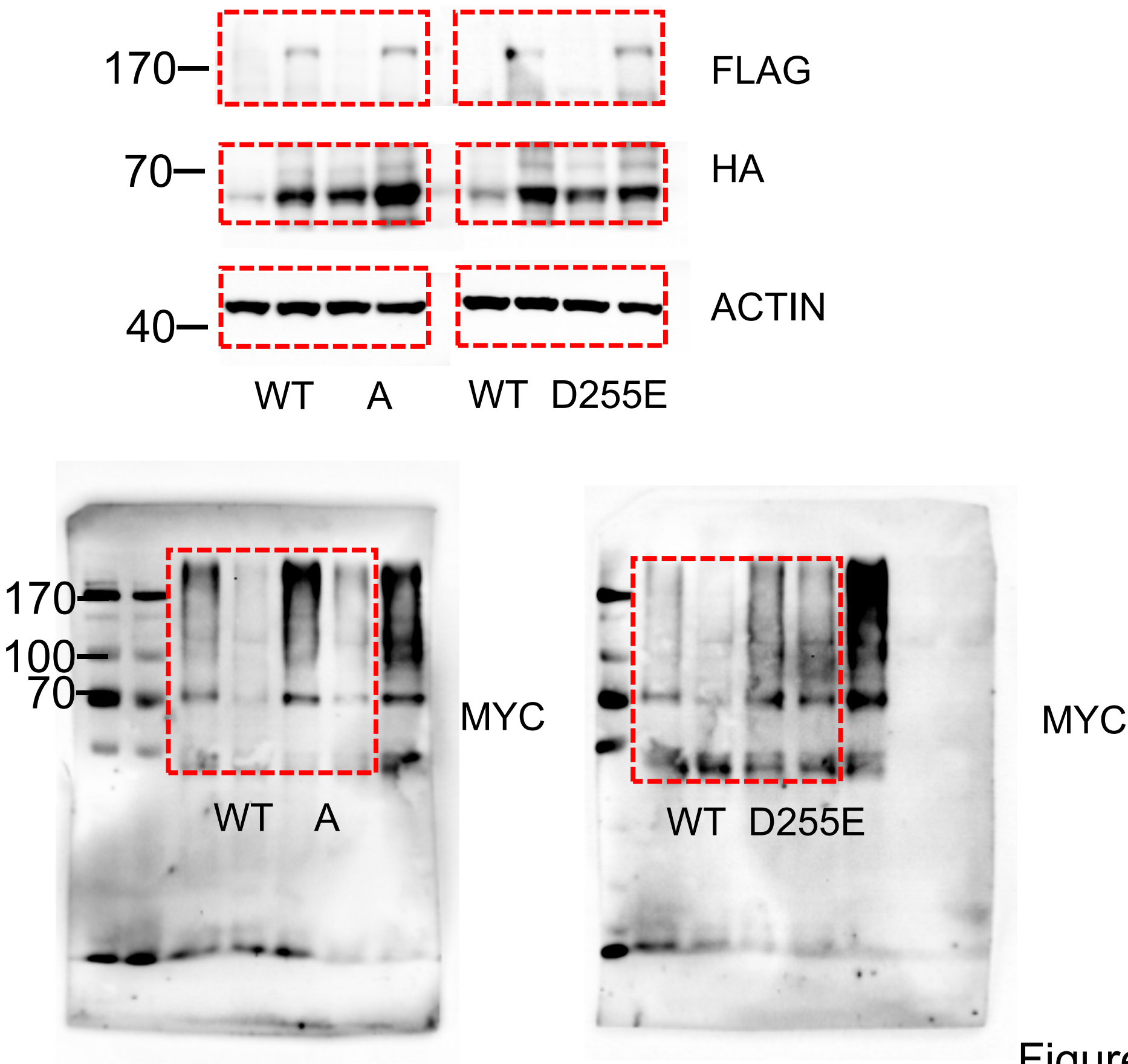

Figure 4A&C

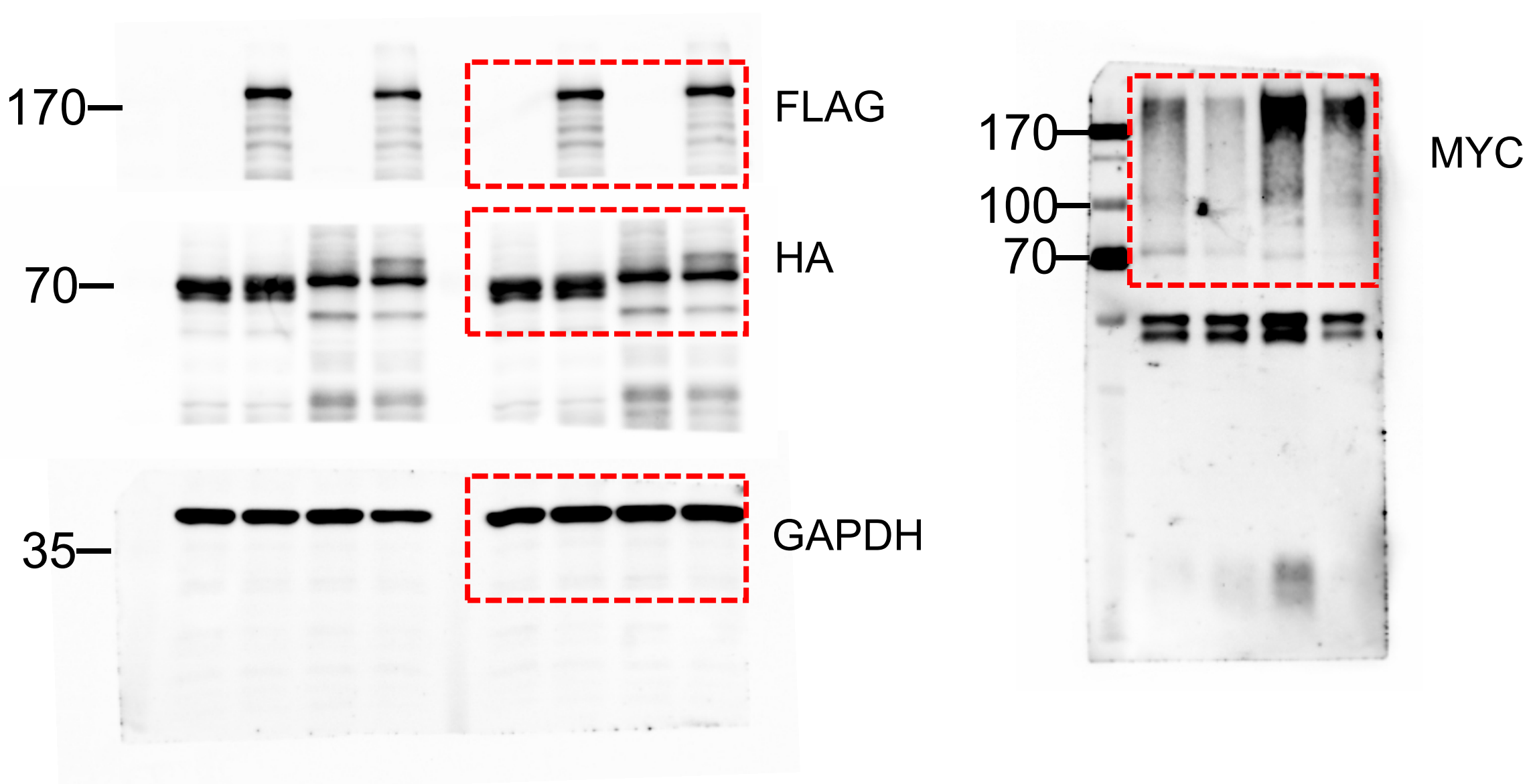

Figure 4B

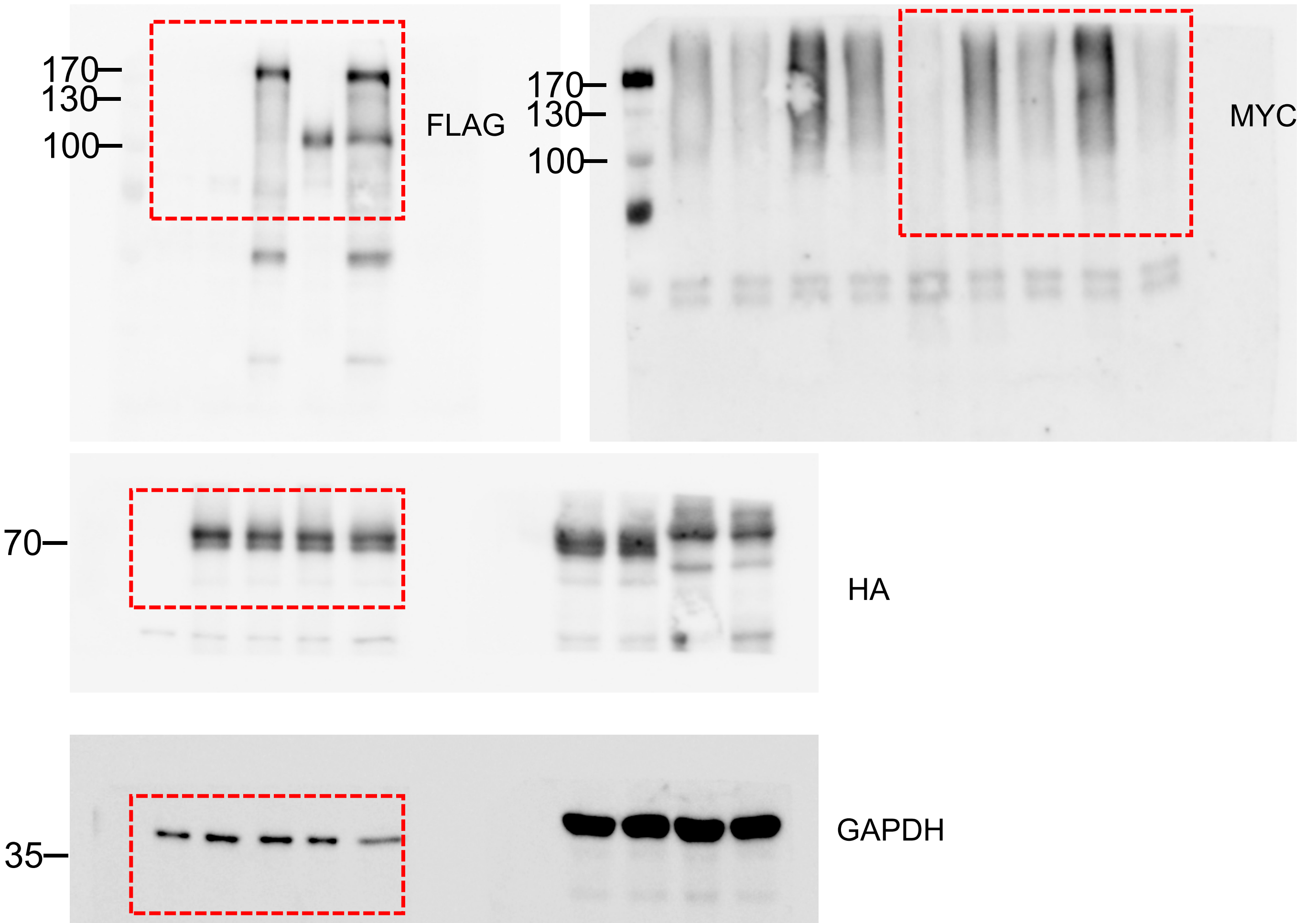

Figure 4D

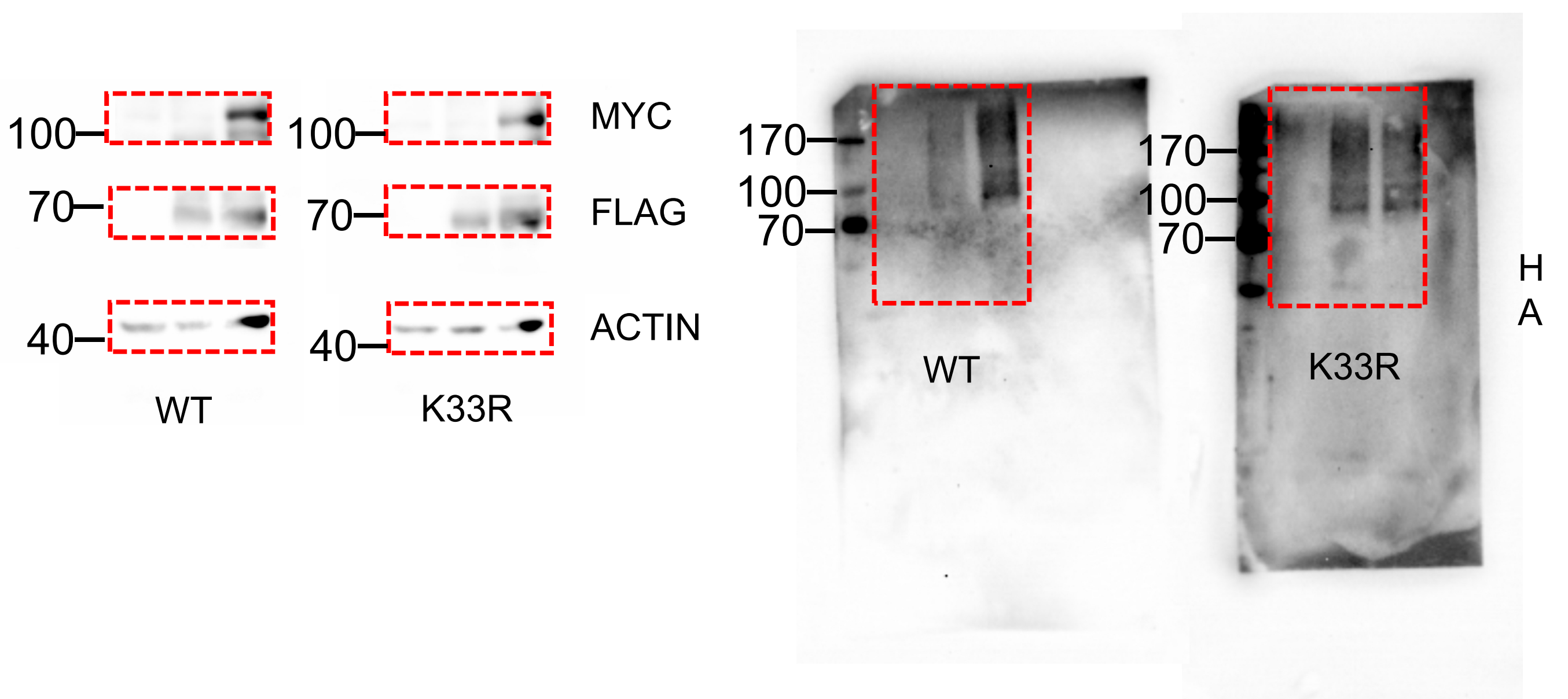

Figure 4E

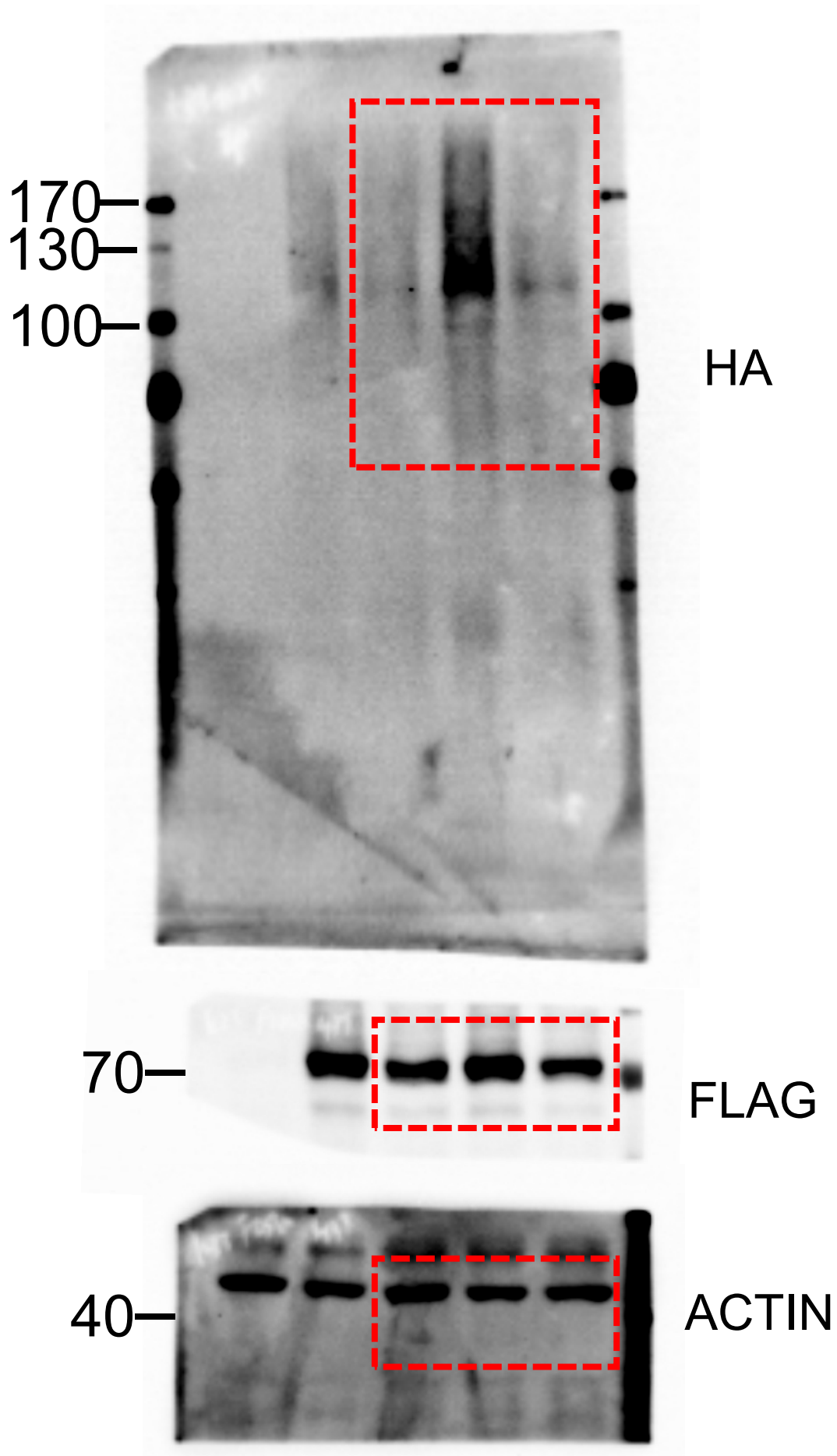

Figure 4F

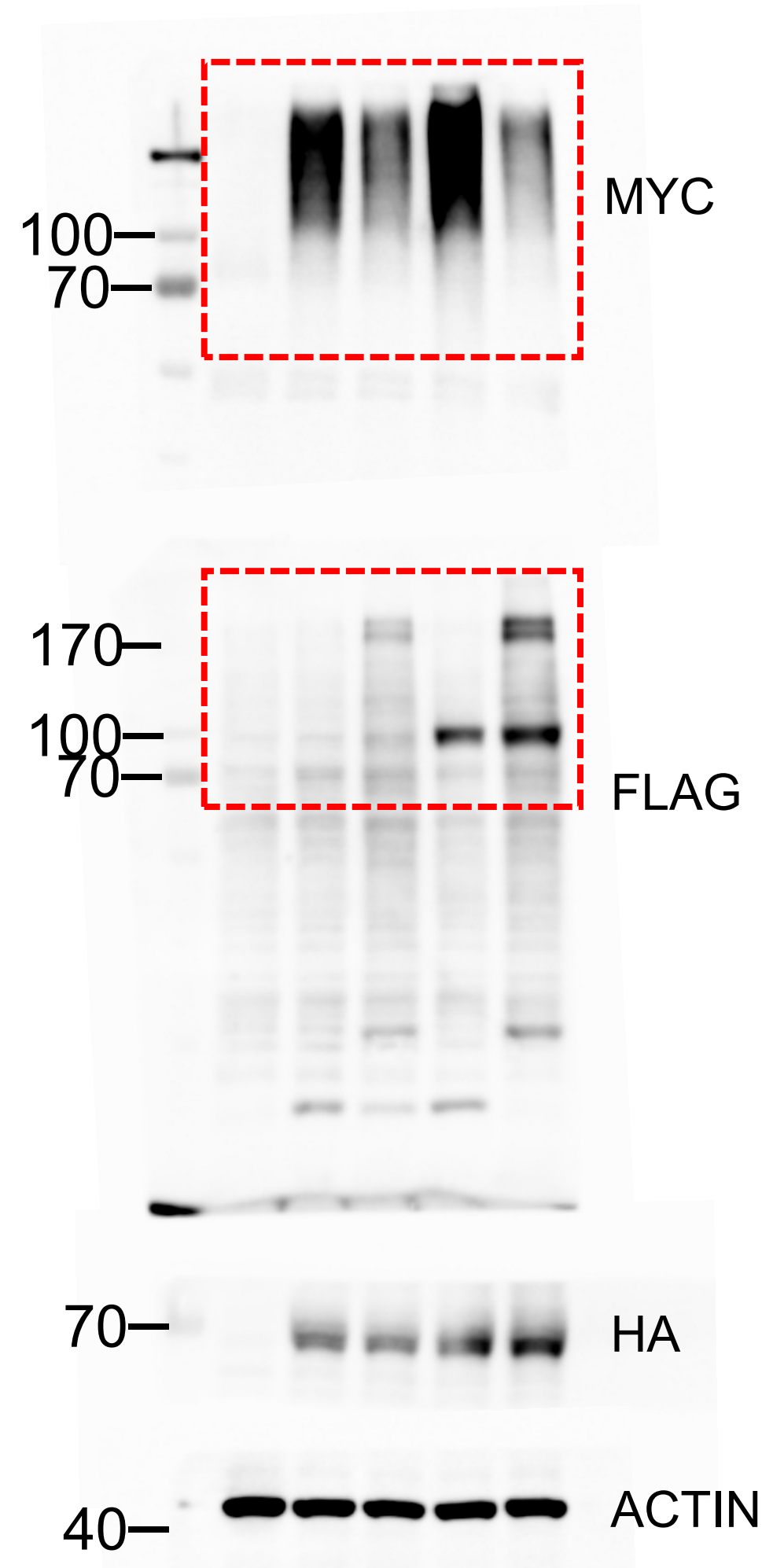

Figure 4G

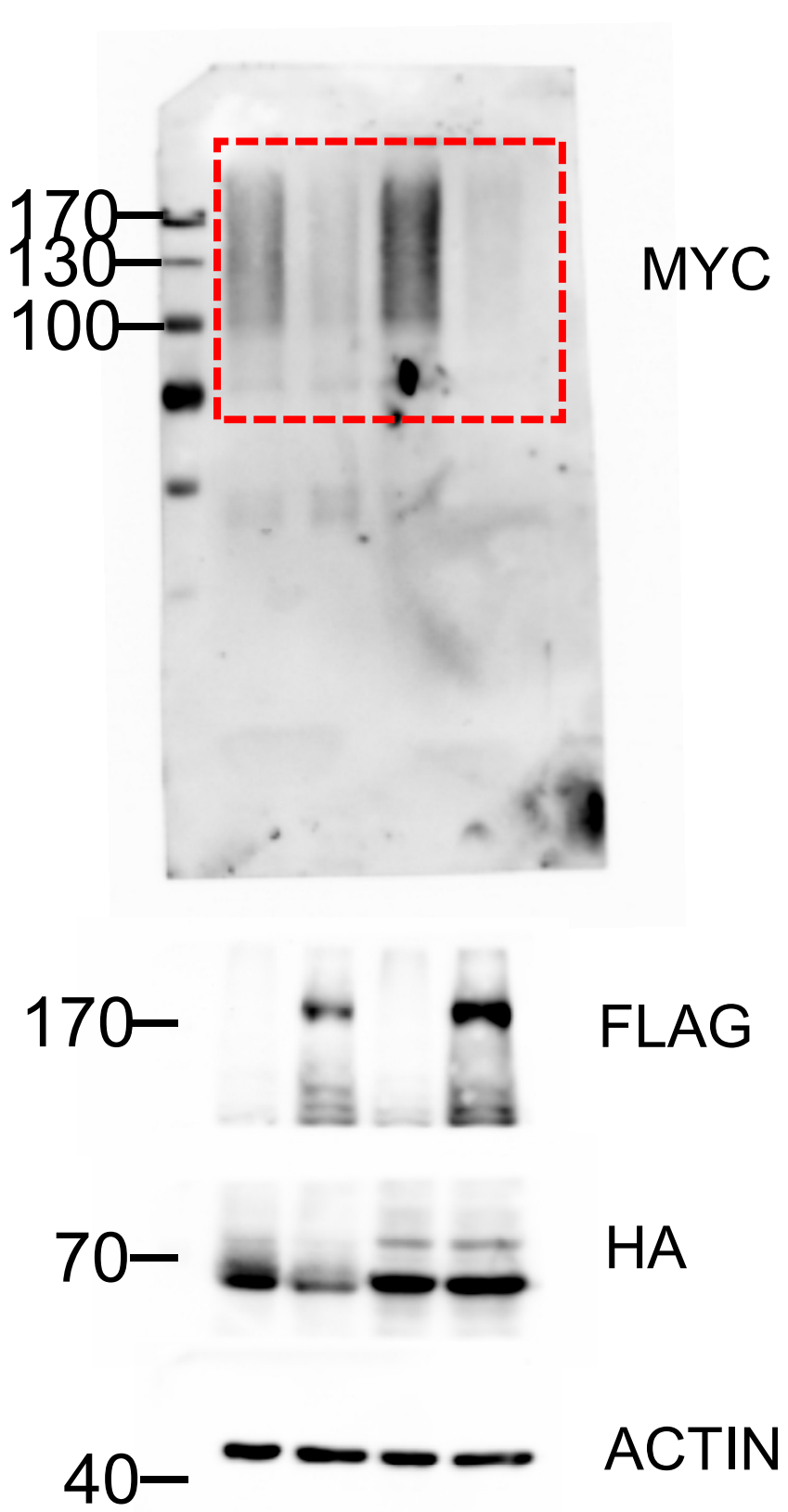

Figure 4H

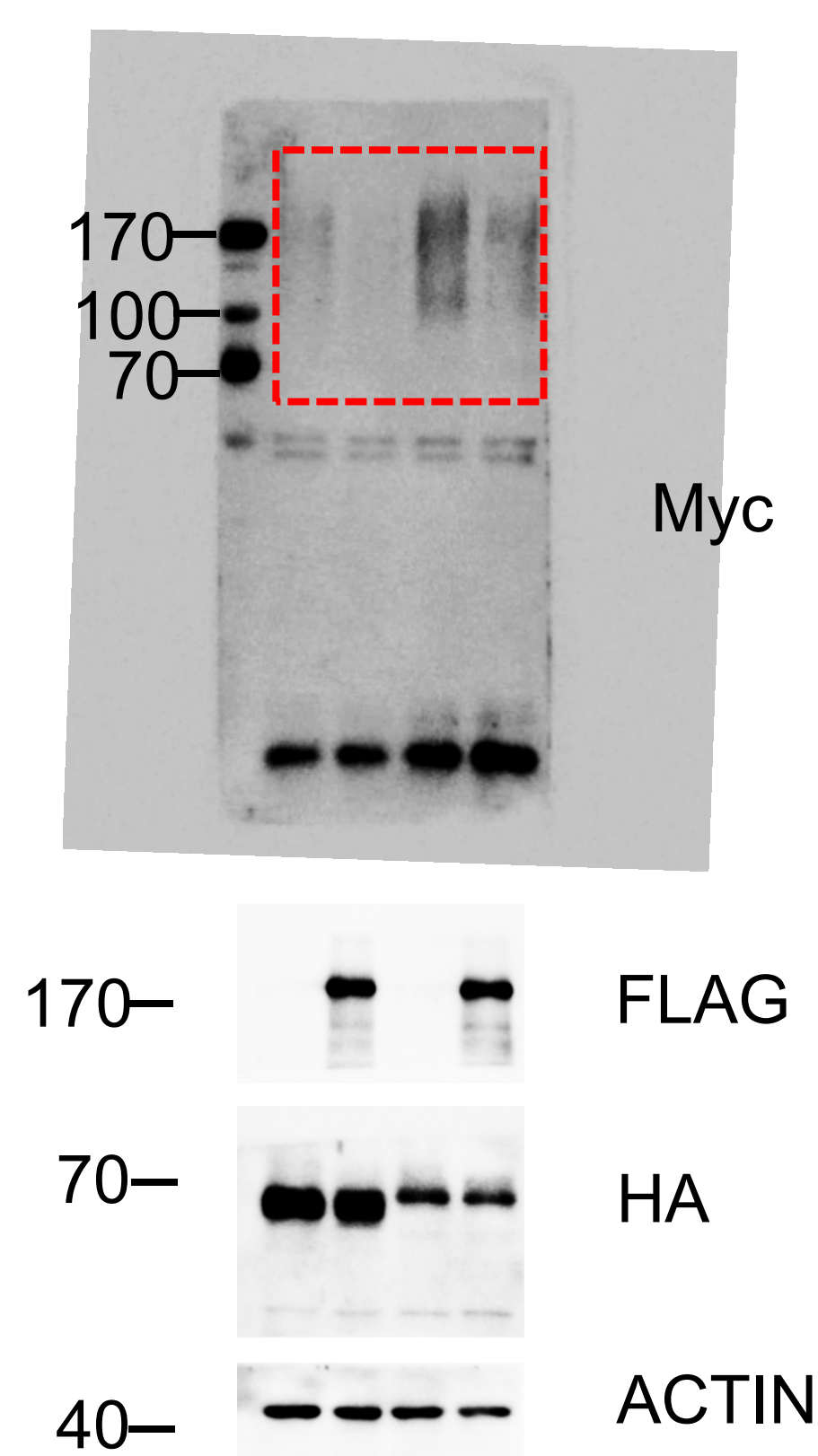

Figure 4I

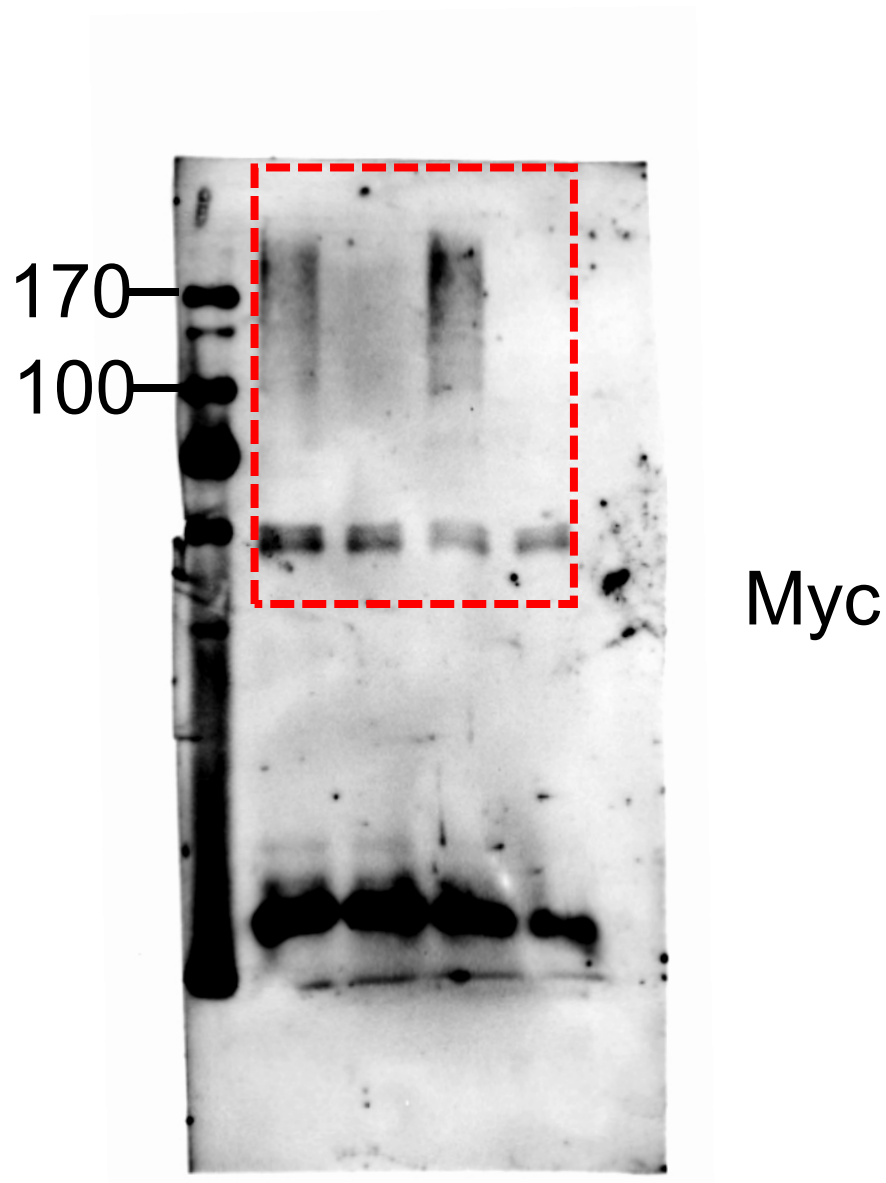

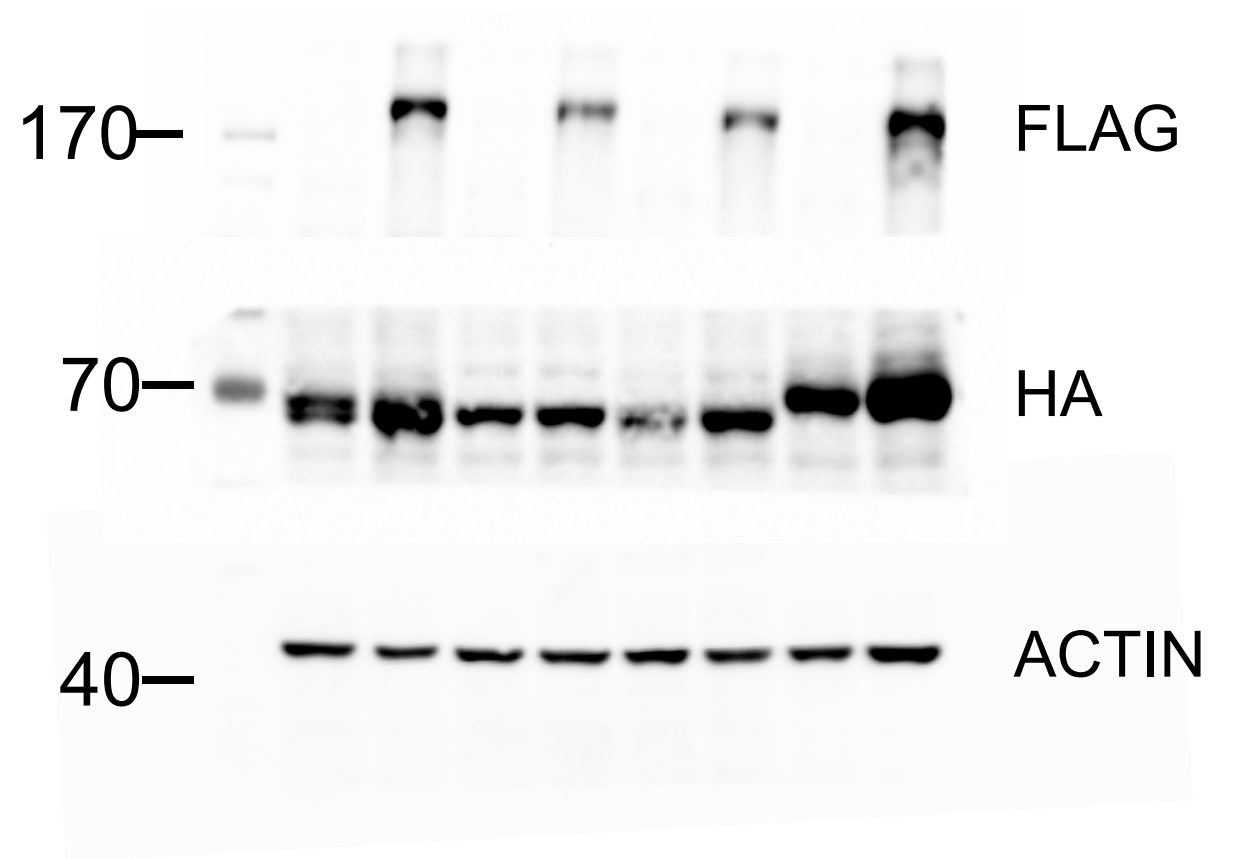

Figure 4K

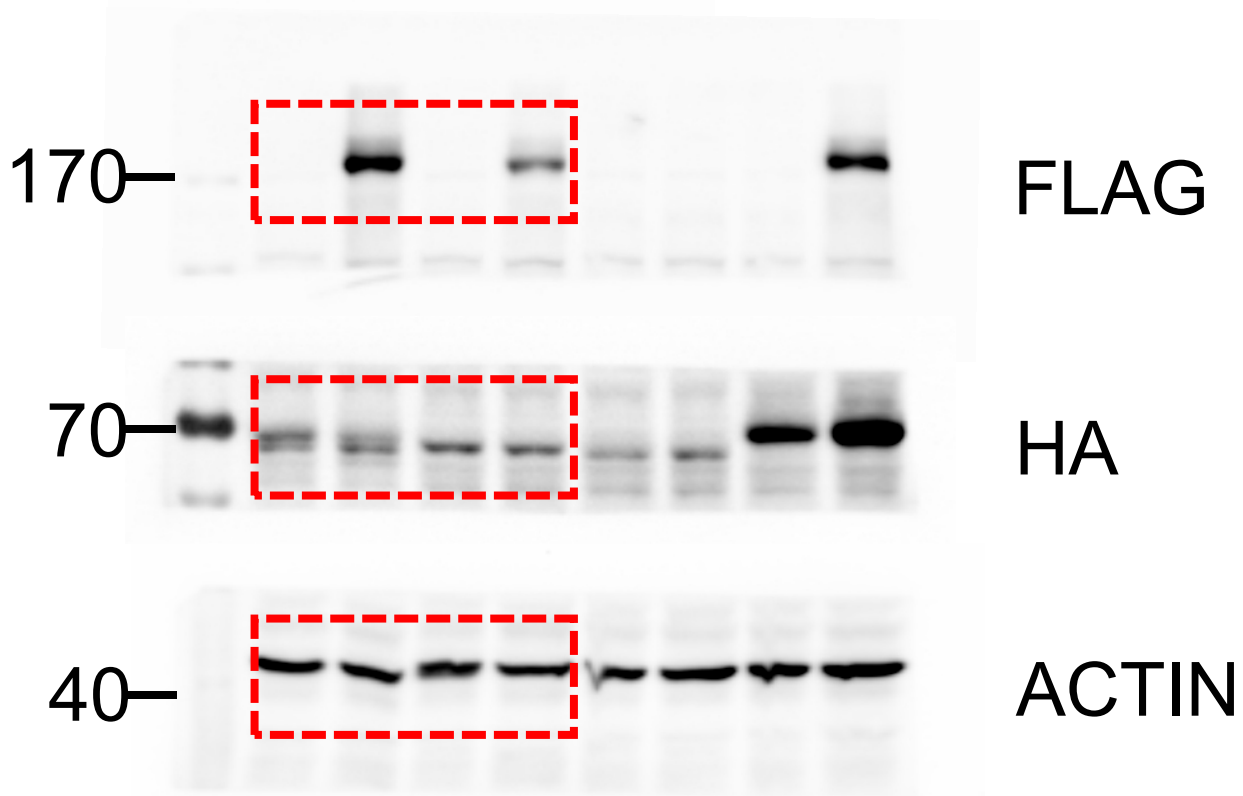

Figure 4J

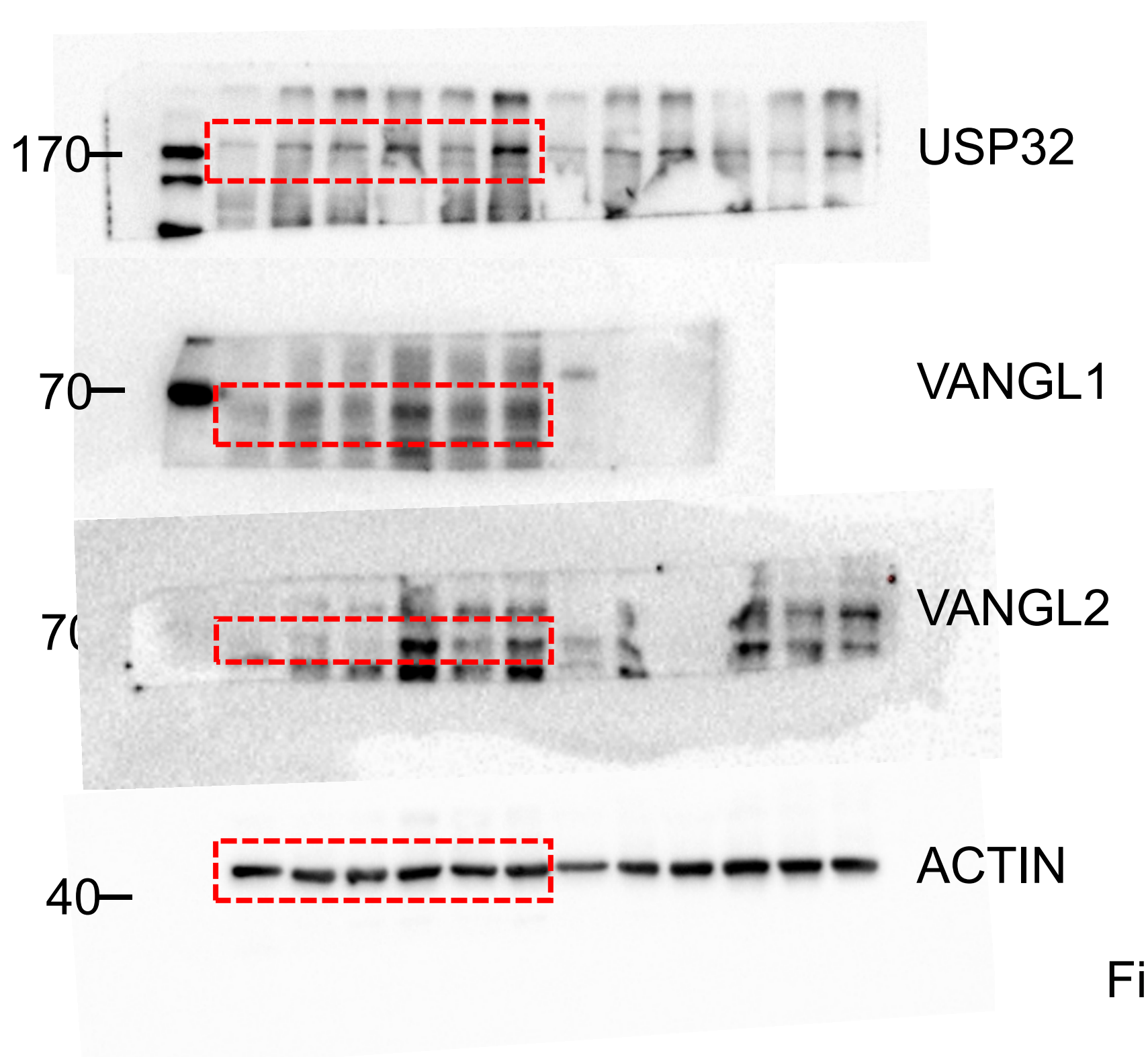

Figure 6D

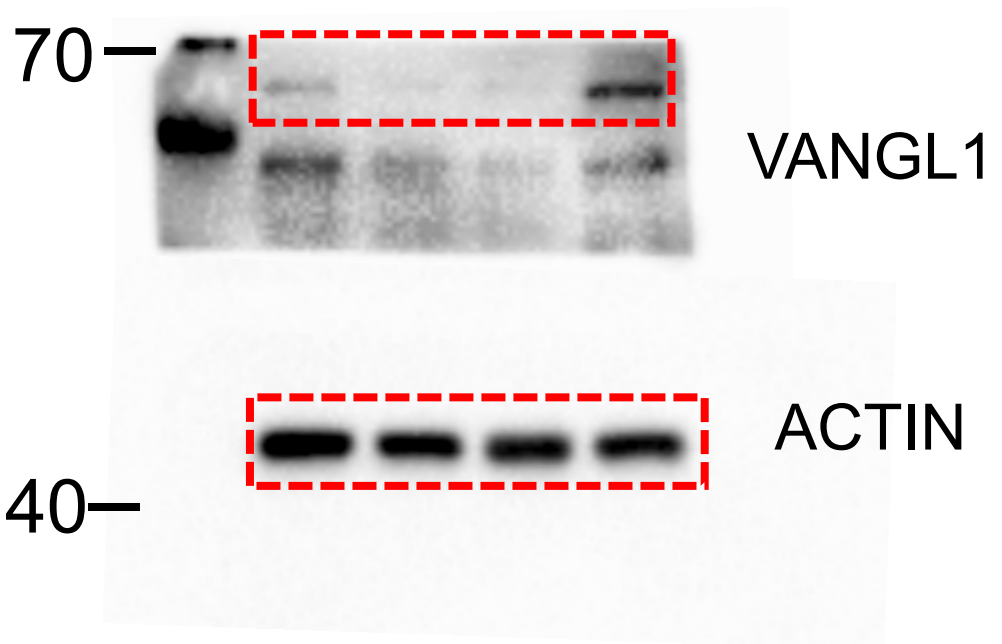

Figure 7A

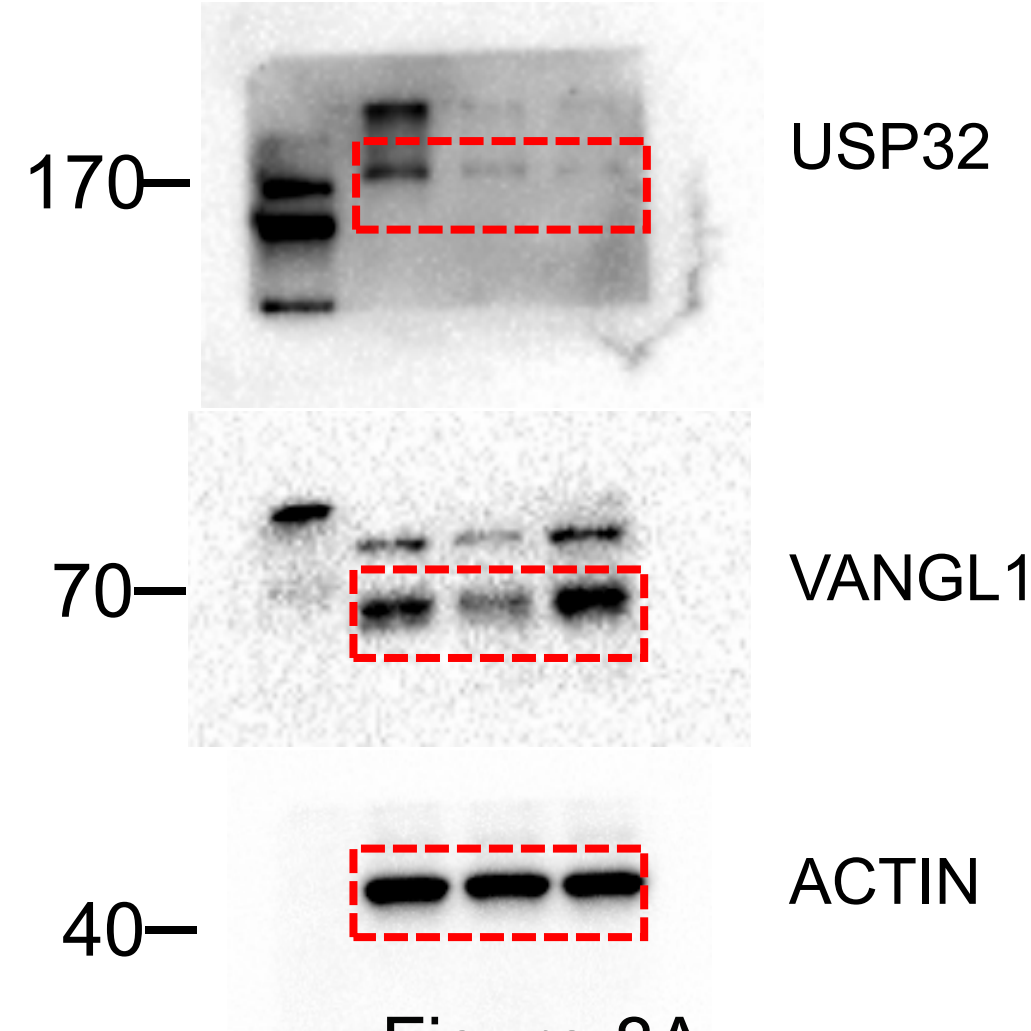

Figure 8A

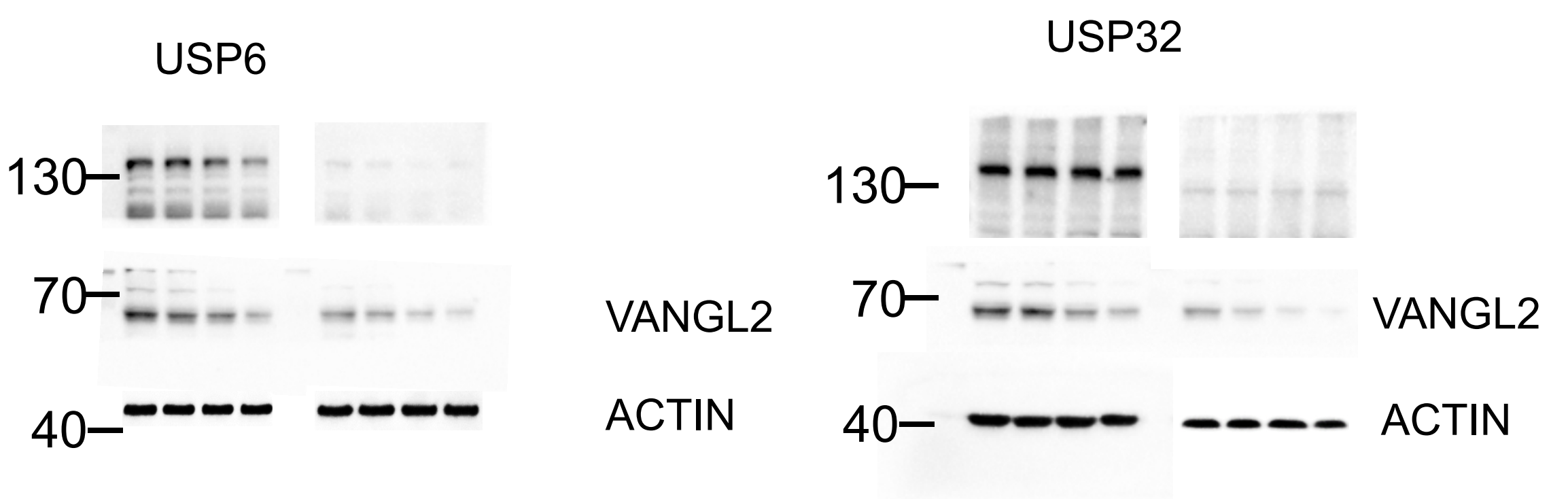

Figure S1A

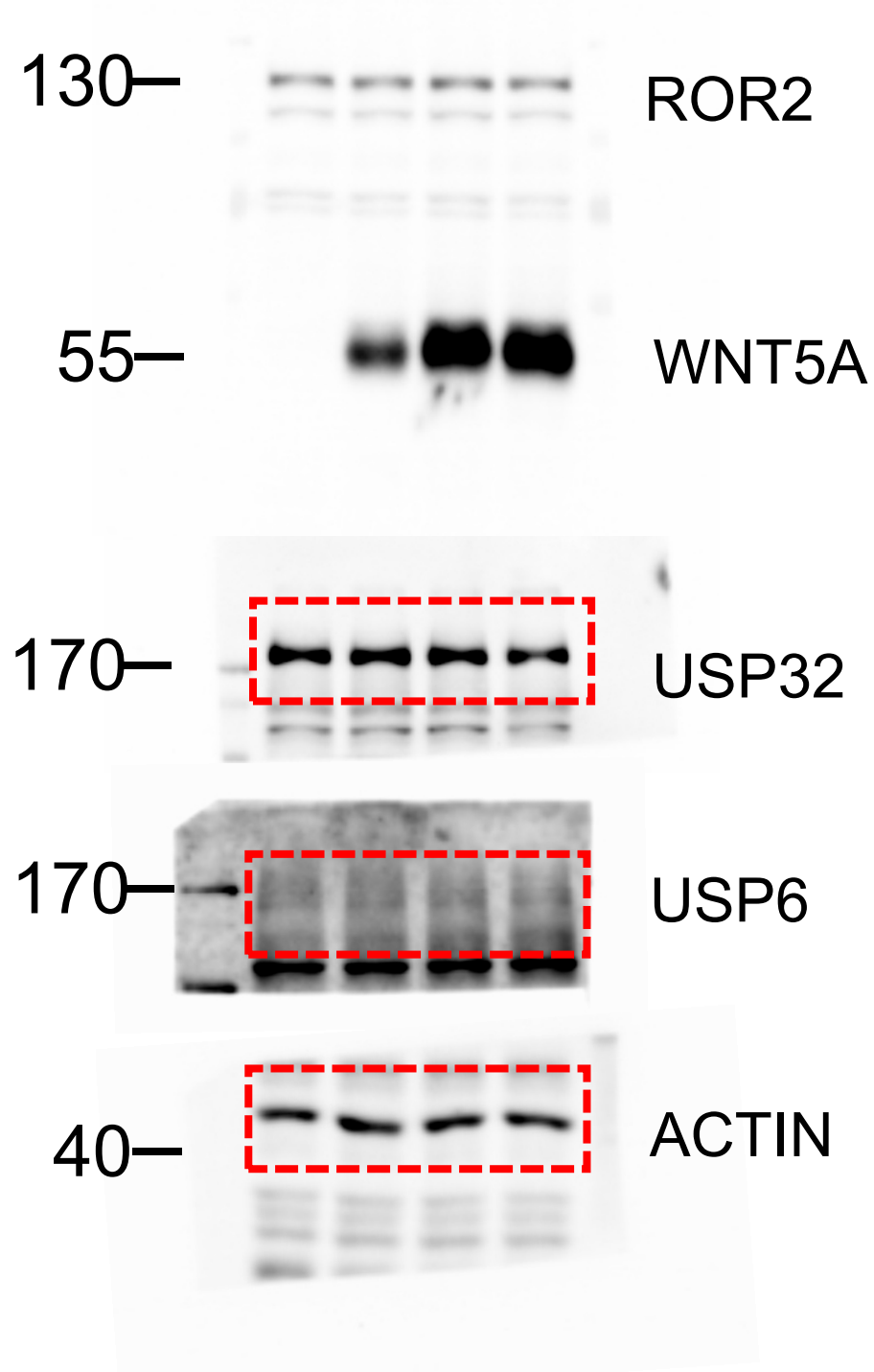

Figure S1B

Figure S2

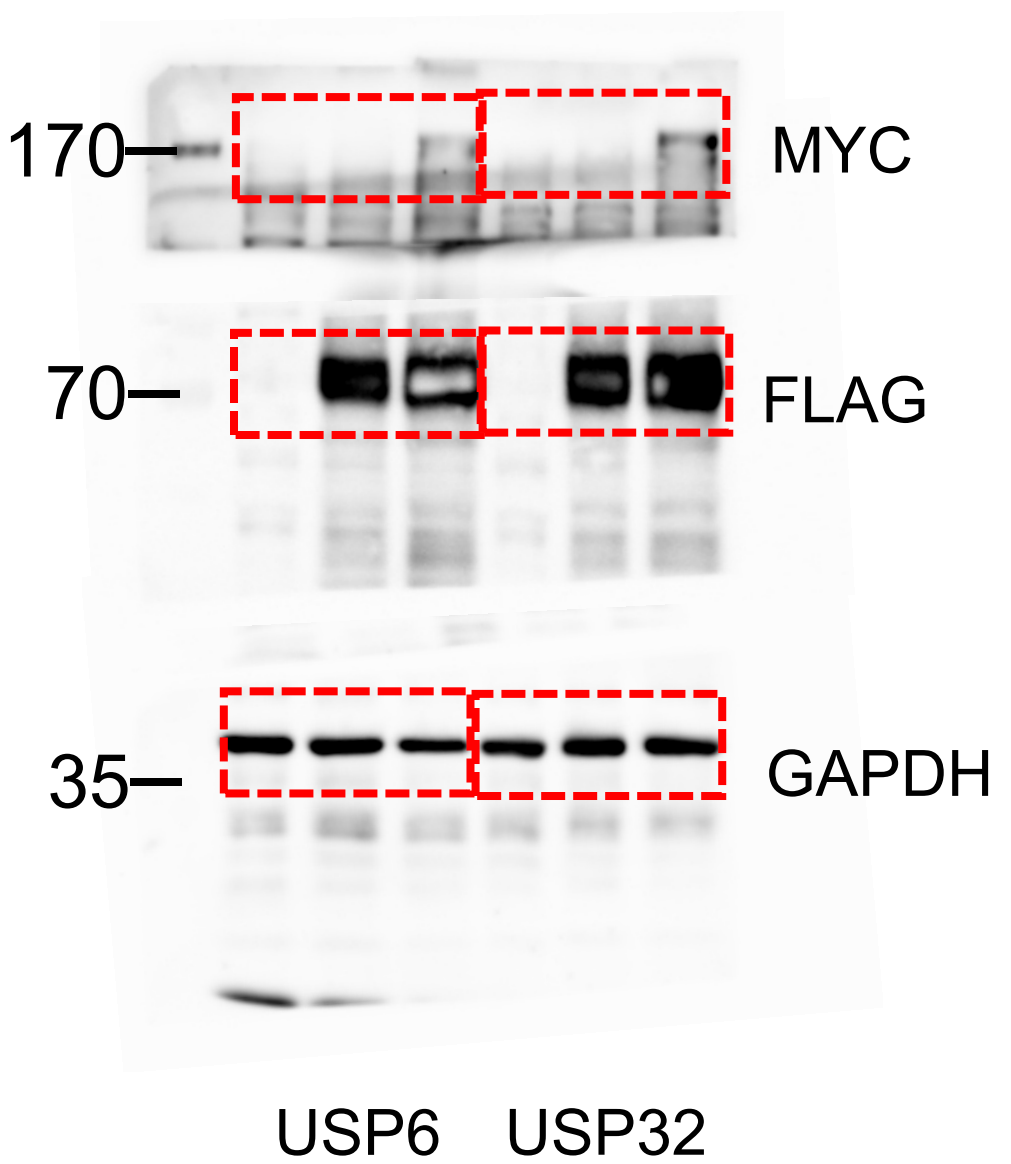
170
70
35
MYC
FLAG
GAPDH
USP6
USP32

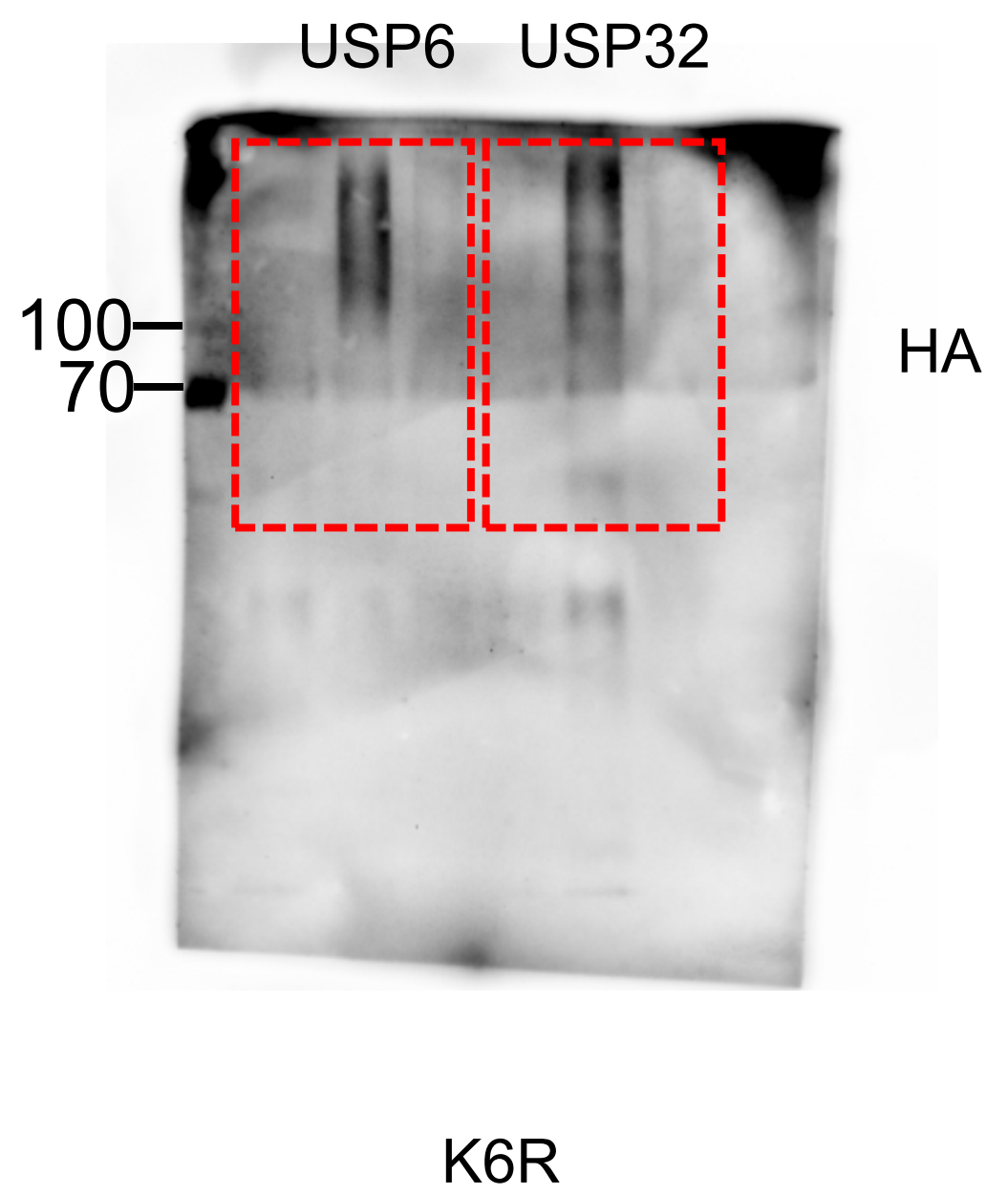
USP6
USP32
100
70
HA
K6R

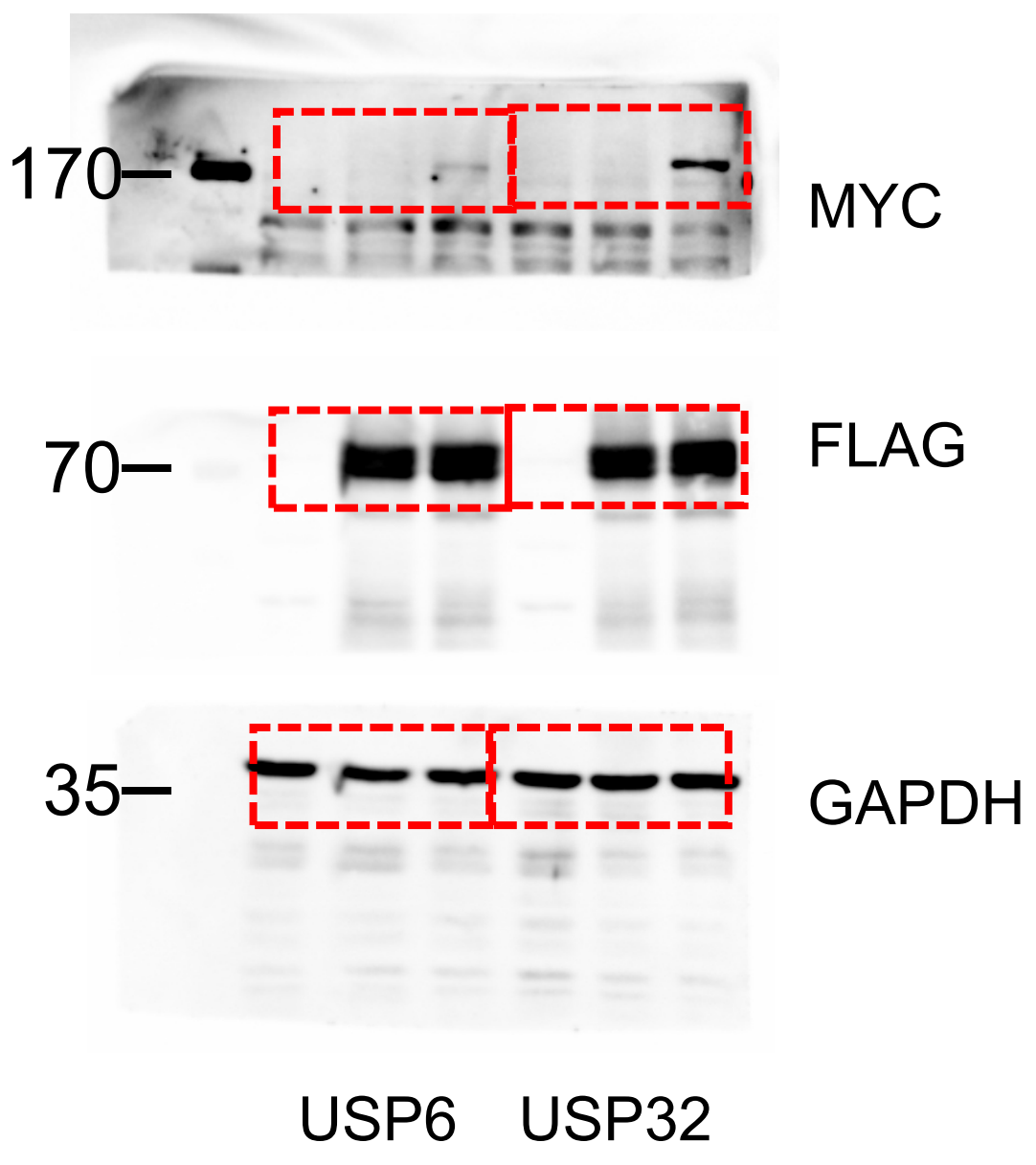
170
70
35
MYC
FLAG
GAPDH
USP6
USP32

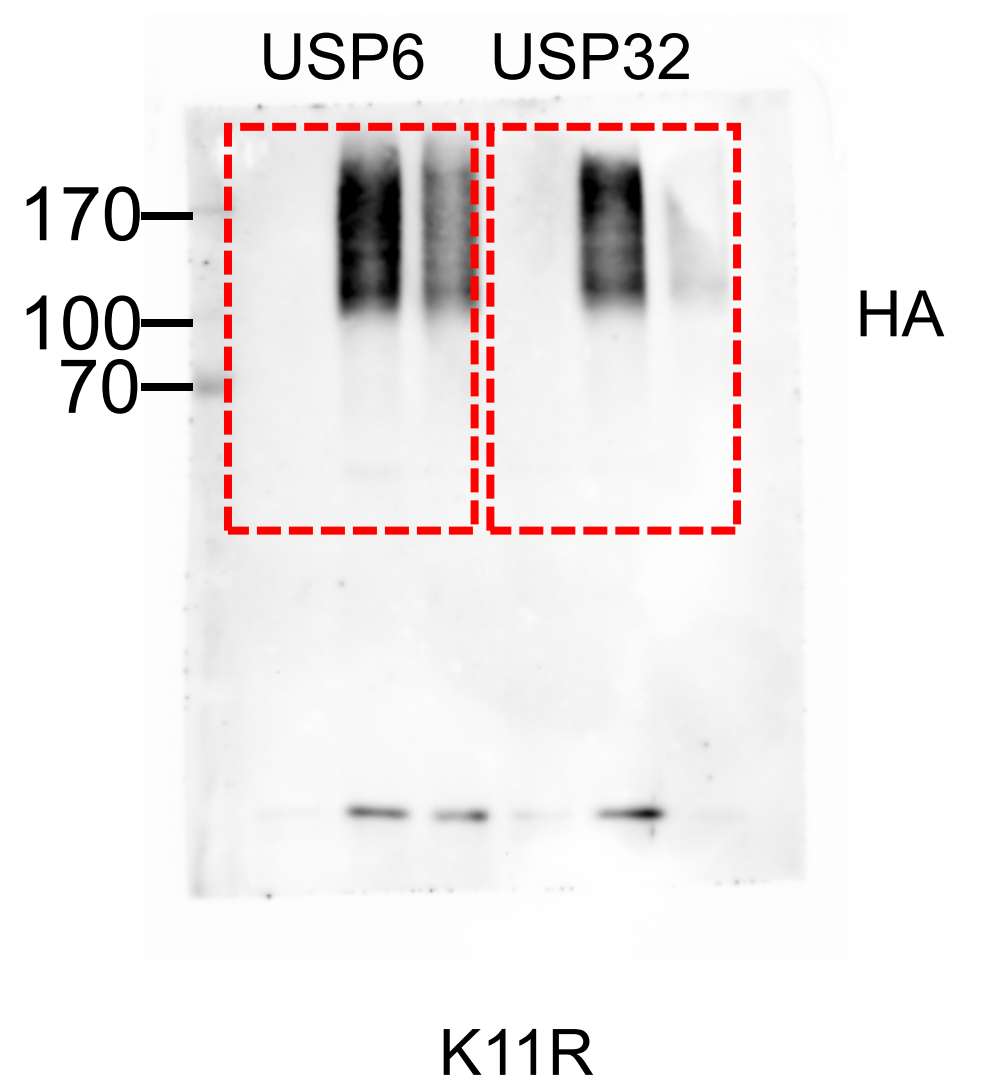
USP6
USP32
170
100
70
HA
K11R

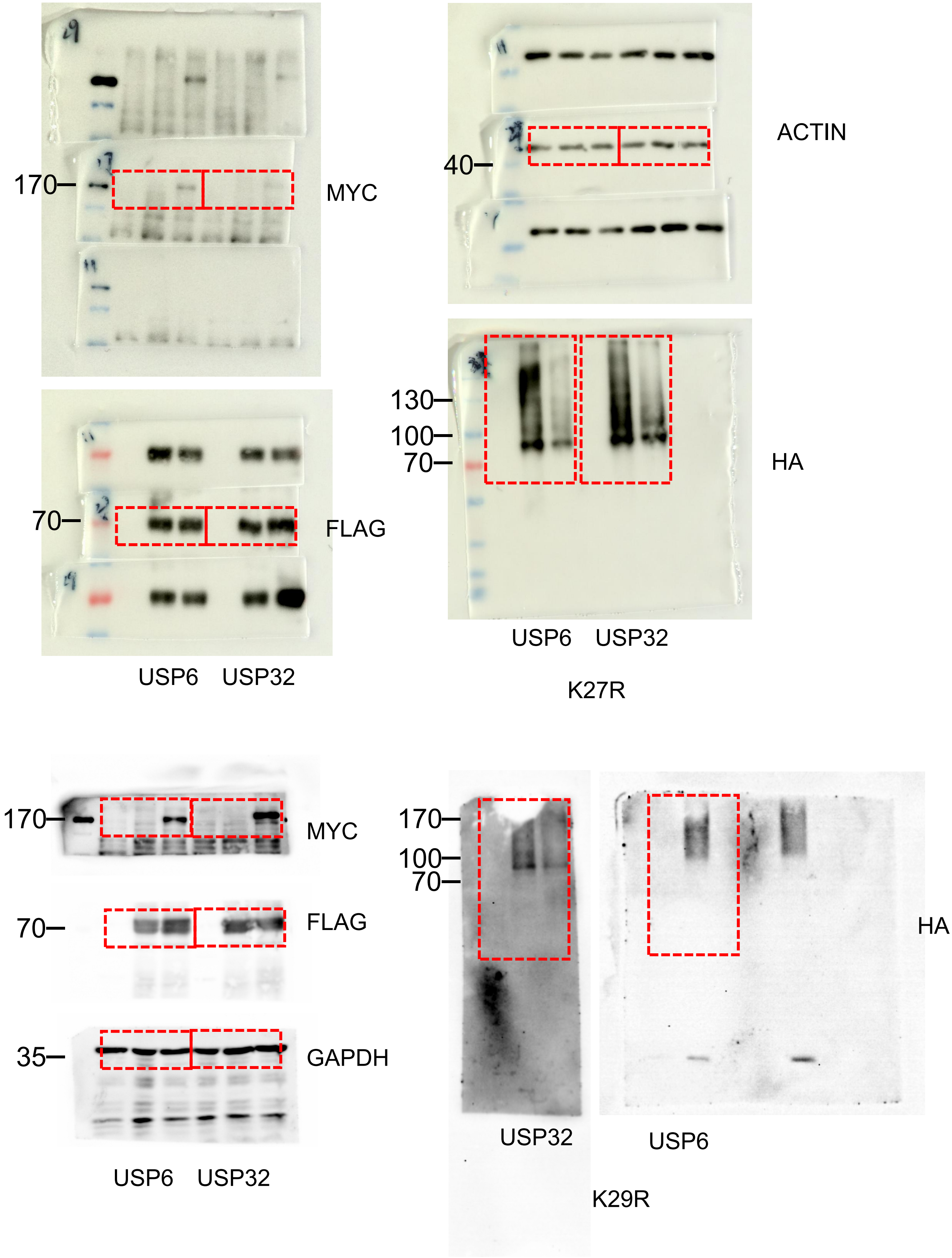
170
MYC
70
FLAG
USP6 USP32
ACTIN
40
130
100
70
HA
USP6 USP32
K27R
170
MYC
70
FLAG
35
GAPDH
USP6 USP32
170
100
70
HA
USP32
USP6
K29R

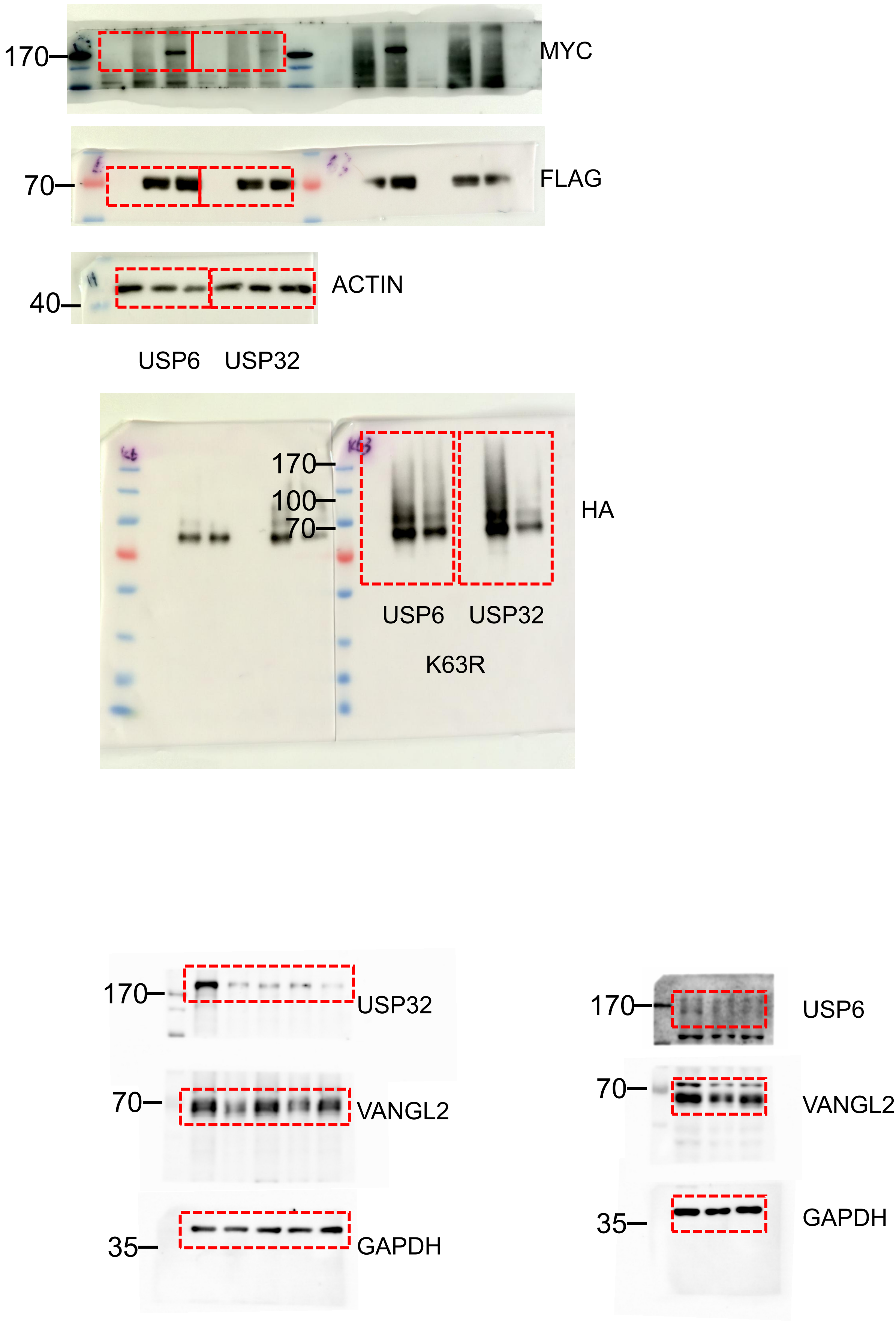
170
MYC
70
FLAG
40
ACTIN
USP6
USP32
170
100
70
HA
USP6
USP32
K63R
170
USP32
70
VANGL2
35
GAPDH
170
USP6
70
VANGL2
35
GAPDH

Figure S3

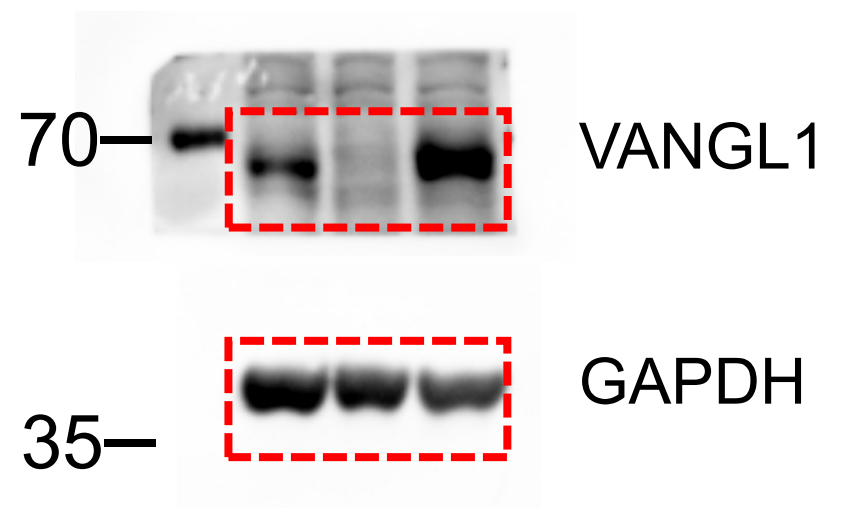

Figure S9A

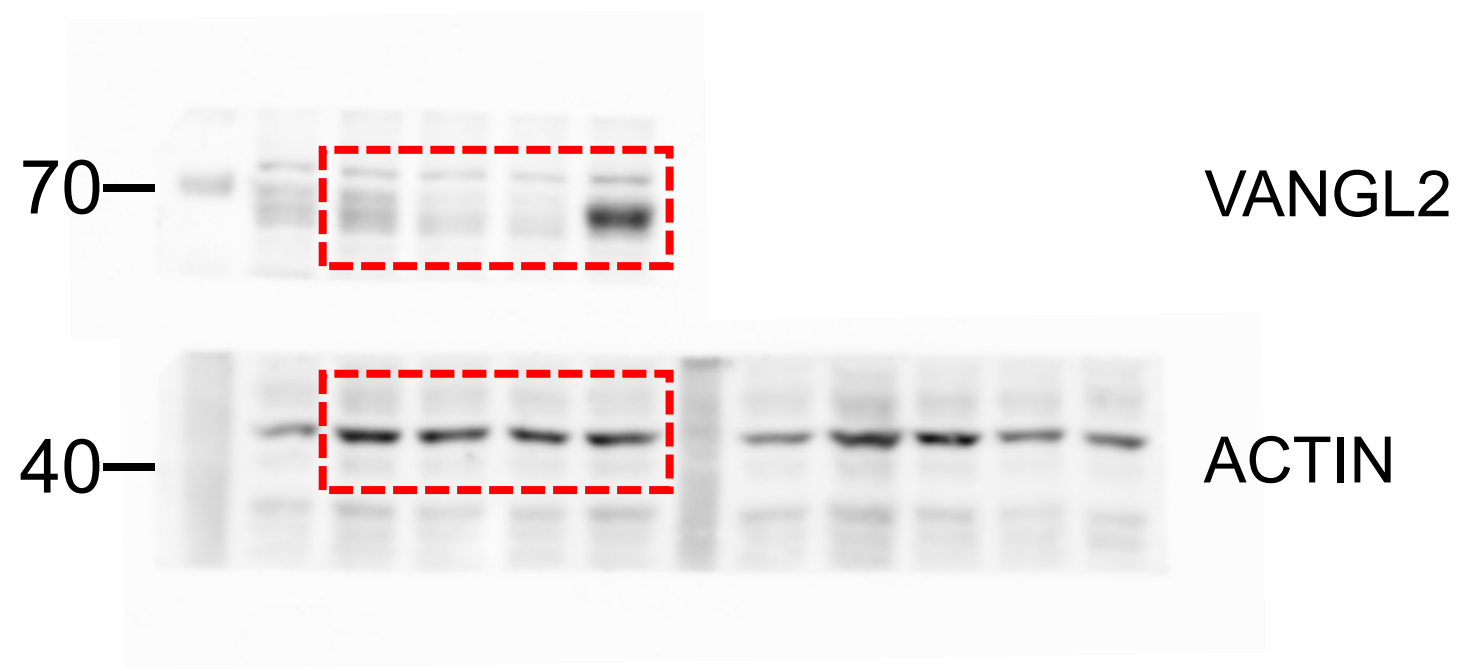

Figure S10A

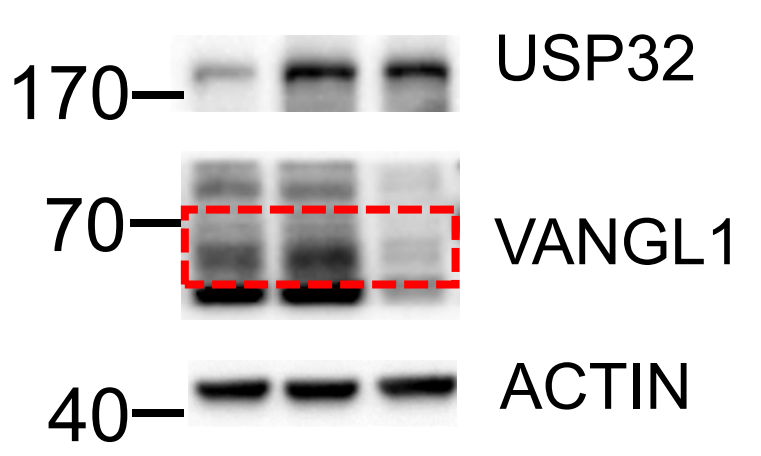

Figure S11

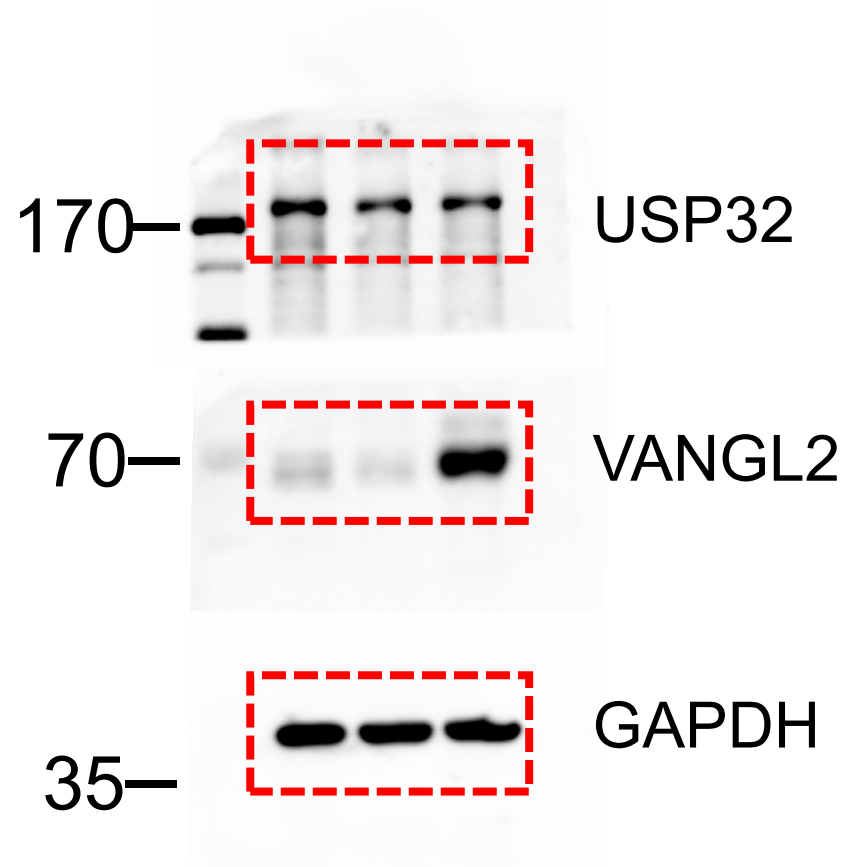

Figure S14A

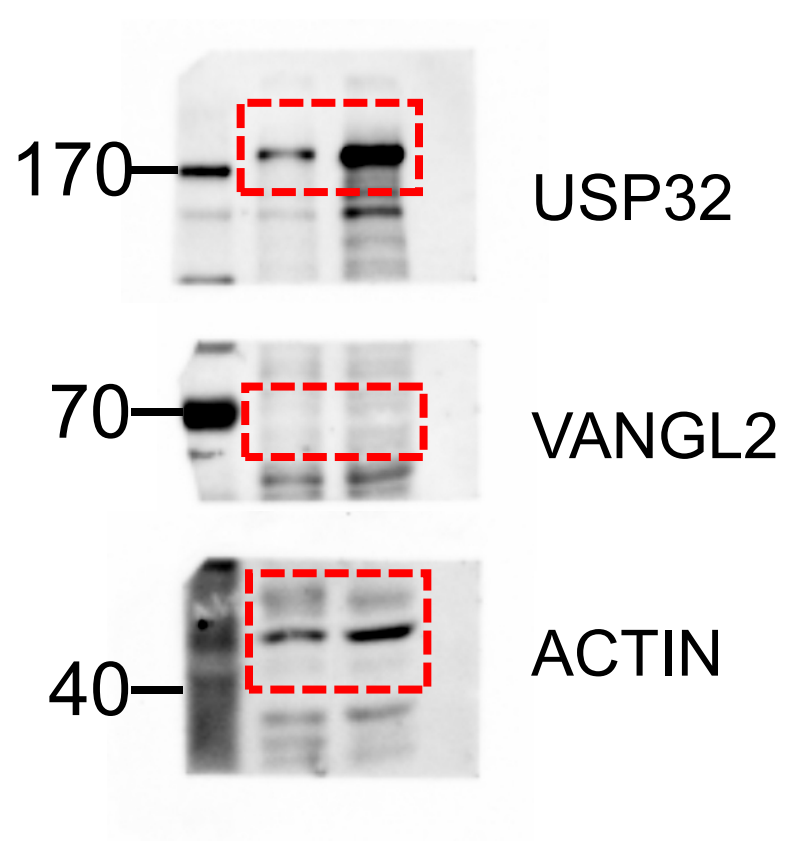

Figure S14F

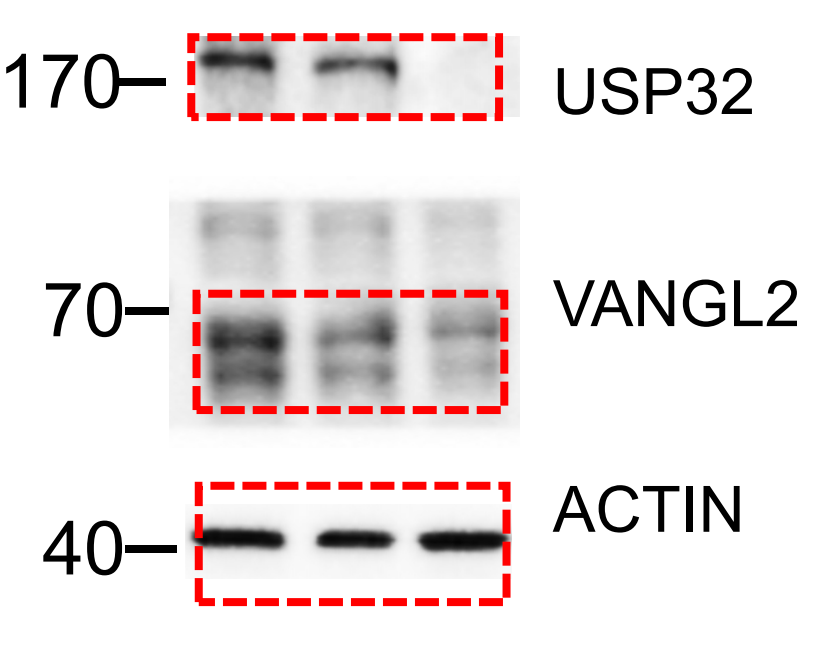

Figure S15B
